# Co-regulation of Iron Metabolism and Virulence Associated Functions by Iron and XibR, a Novel Iron Binding Transcription Factor, in the Plant Pathogen *Xanthomonas*

**DOI:** 10.1371/journal.ppat.1006019

**Published:** 2016-11-30

**Authors:** Sheo Shankar Pandey, Pradeep Kumar Patnana, Santosh Kumar Lomada, Archana Tomar, Subhadeep Chatterjee

**Affiliations:** 1 Centre for DNA Fingerprinting and Diagnostics, Nampally, India; 2 Graduate studies, Manipal University, Manipal, India; University of California Riverside, UNITED STATES

## Abstract

Abilities of bacterial pathogens to adapt to the iron limitation present in hosts is critical to their virulence. Bacterial pathogens have evolved diverse strategies to coordinately regulate iron metabolism and virulence associated functions to maintain iron homeostasis in response to changing iron availability in the environment. In many bacteria the ferric uptake regulator (Fur) functions as transcription factor that utilize ferrous form of iron as cofactor to regulate transcription of iron metabolism and many cellular functions. However, mechanisms of fine-tuning and coordinated regulation of virulence associated function beyond iron and Fur-Fe^2+^ remain undefined. In this study, we show that a novel transcriptional regulator XibR (named *X* *anthomonas* iron binding regulator) of the NtrC family, is required for fine-tuning and co-coordinately regulating the expression of several iron regulated genes and virulence associated functions in phytopathogen *Xanthomonas campestris* pv. *campestris* (Xcc). Genome wide expression analysis of iron-starvation stimulon and XibR regulon, GUS assays, genetic and functional studies of *xibR* mutant revealed that XibR positively regulates functions involved in iron storage and uptake, chemotaxis, motility and negatively regulates siderophore production, in response to iron. Furthermore, chromatin immunoprecipitation followed by quantitative real-time PCR indicated that iron promoted binding of the XibR to the upstream regulatory sequence of operon’s involved in chemotaxis and motility. Circular dichroism spectroscopy showed that purified XibR bound ferric form of iron. Electrophoretic mobility shift assay revealed that iron positively affected the binding of XibR to the upstream regulatory sequences of the target virulence genes, an effect that was reversed by ferric iron chelator deferoxamine. Taken together, these data revealed that how XibR coordinately regulates virulence associated and iron metabolism functions in Xanthomonads in response to iron availability. Our results provide insight of the complex regulatory mechanism of fine-tuning of virulence associated functions with iron availability in this important group of phytopathogen.

## Author Summary

Pathogenic bacteria exhibit tight regulation of iron homeostasis in order to meet iron requirements of living in different environmental conditions, including in the host. The ferric uptake regulator (Fur) regulates the expression of genes involved in iron metabolism in response to change in iron availability in several bacteria. However, beyond Fur, mechanisms of fine-tuning expression of iron regulated genes and virulence associated functions in response to iron availability is largely undefined. Here, we show that a novel ferric iron binding transcription factor, XibR, is required for optimum virulence in phytopathogen *Xanthomonas campestris* pv. *campestris* (Xcc) by coordinately regulating expression of genes involved in iron metabolism and several virulence associated function such as chemotaxis and motility. XibR directly binds to the upstream regulatory sequence of chemotaxis, and flagellar cluster in the presence of ferric form of iron. Furthermore, the *xibR* mutant exhibited reduced growth and intracellular iron content under low-iron condition, which were phenocopied by mutants in the iron storage and uptake genes. This study provides for the mechanistic insight for the first time into the involvement of a ferric binding transcription factor in the coordinated regulation of iron metabolism and virulence associated functions.

## Introduction

Iron homeostasis is vital for survival and cellular metabolism in many organisms. Bacteria maintain cellular iron homeostasis by coordinately regulating iron uptake, metabolism and storage, to achieve sufficient iron under iron-replete condition, and to store intracellular iron surplus for utilization under condition of iron limitation [1]. Iron is required for virulence of several animal and plant pathogenic bacteria [1–3]. The availability of iron within the host plays a critical role in the growth and survival of the pathogens. In animal hosts, iron-withholding strategies are employed to limit iron availability to infecting pathogens [1]. Similarly, in plants, several studies have shown that iron availability is likely to be a limiting factor for pathogen growth within host [2,3].

Bacteria employ a variety of strategies to sequester iron from the environment for survival. These include secretion and uptake of low molecular weight iron chelators called siderophores, transport of the ferrous form of iron by the ferrous iron transporter (Feo), several metal-type ABC transporters [1,4]. Certain pathogenic bacteria are also able to utilize host-iron complexes such as transferrin, lactoferrin and heme, when exogenous iron sources are restricted [1,5]. However, excess of free iron is toxic to the cell as it causes the production of Reactive Oxygen Species (ROS) by the Fenton reaction [4]. Hence, bacteria tightly coordinate the expression of the iron homeostasis machinery which includes iron uptake, storage and distribution in response to iron availability to ensure proper iron homeostasis. In addition, it has been shown that pathogenic bacteria utilizes iron as regulatory signal to coordinately regulate the expression of virulence genes such as toxins, hemolysins, and hydrolyzing enzymes, as low-iron conditions triggers the expression of iron uptake systems as well virulence associated factors, mimicking limited iron availability inside the host environment [6,7].

In many bacteria Ferric-uptake regulator (Fur) is involved in the coordinated regulation of gene expression in response to iron availability. Fur utilizes Fe^2+^ as a cofactor and represses the expression of iron uptake and metabolism genes under iron sufficiency, and causes de-repression in the absence of Fe^2+^ under conditions of iron restriction. Fur-Fe^2+^ also has been reported to be involved in the positive regulation of expression of genes involved in iron storage proteins, superoxide dismutase, and catalase. In addition to regulating genes involved in iron uptake and metabolism, Fur has been shown to regulate diverse cellular process such as respiration, TCA cycle, glycolysis, oxidative stress [7–9]. However, mechanism of fine-tuning iron metabolism and virulence associated functions beyond ferrous responsive Fur-like transcription factor (TF) remains undefined in pathogenic bacteria.

Bacteria belonging to the genus *Xanthomonas* causes diseases in several economically important plants [10,11]. Xanthomonads encodes an *xss* (*X* *anthomonas* siderophore synthesis) operon which is required for the production of siderophore vibrioferrin under iron-restricted conditions, and iron metabolism plays a critical role in their virulence [12–14]. These phytopathogen use cell-cell signaling mediated by diffusible quorum sensing signal molecule to regulate the expression of iron uptake and metabolism functions contributing to virulence and growth within host [13,15]. In *Xanthomonas campestris* pv. *campestris* (Xcc) and *Xanthomonas oryzae* pv. *oryzae* (Xoo), which are important models to study bacterial phytopathogenesis, it has been shown that Fur is involved in the suppression of siderophore production and *fur* mutants are deficient in virulence and hypersensitive to oxidative stress [16]. However, little is known about mechanisms of fine-tuning expression of iron regulated genes, beyond iron regulation mediated via cell-cell signaling and Fur in this important group of phytopathogens.

Xcc produces moderate amount of siderophore only in iron-limiting conditions. In order to gain insight into iron metabolism and regulatory functions involved in iron metabolism, we performed a genetic screen to identify mutants overproducing siderophores. Three mutants were identified that had transposon insertions in an *ntrC* family of transcription factor (*XC*_3760; named *xibR*; *X* *anthomonas* iron binding regulator), that significantly overproduce siderophore compared to the parental wild-type strain (Xcc 8004).

NtrC family of transcription factors has been shown to be involved in the regulation of diverse physiological process such as extracellular polysaccharide production, nitrogen metabolism, biofilm formation in diverse bacteria [17–20]. However, role of NtrC family of transcription factor in regulation of iron metabolism and sensing has not been identified.

In this study, we show that the Xcc XibR (an NtrC family of response regulator) is involved in global regulation of functions involved in iron uptake, storage and virulence in response to changes in iron availability. XibR positively regulates motility and chemotaxis in response to iron starvation and also contributes to biofilm formation. Genome wide transcriptional analysis of iron starvation stimulon and XibR regulon indicated that in Xcc, XibR regulates the production of virulence associated function in response to iron availability. Our results provide insight of the mechanism of fine-tuning of virulence associated functions with iron availability in this important phytopathogen.

## Results

### *xibR* regulates siderophores production in *Xanthomonas campestris* pv. *campestris*

**Fig 1:**
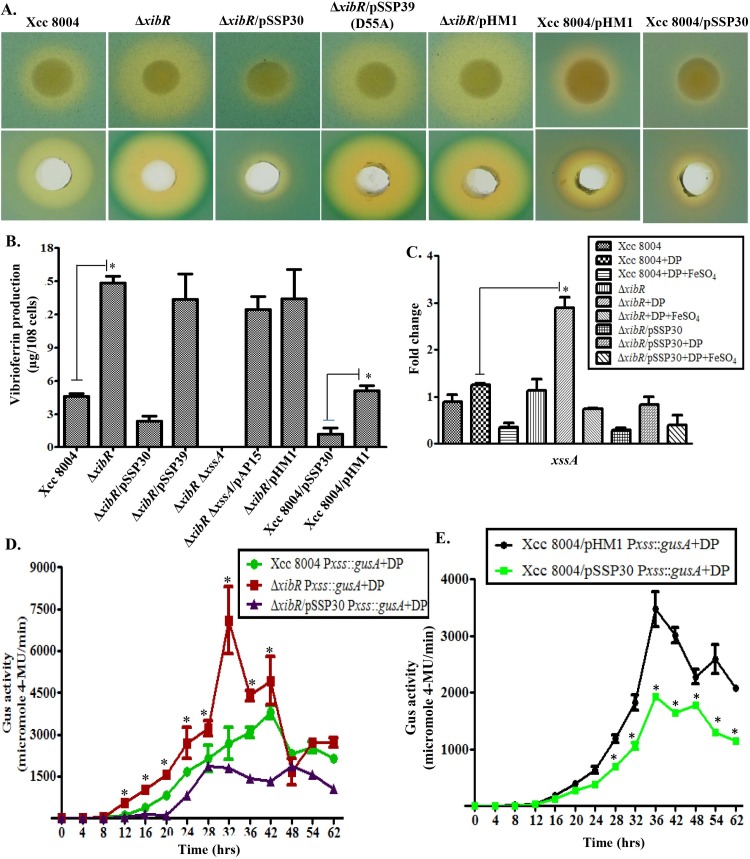
*xibR* suppresses siderophore production in Xcc. (A) Upper lane; siderophore production by Xcc strains on PSA-CAS plates containing 75 μM ferrous iron chelator 2,2′-Dipyridyl (DP) (PSA-CAS + DP). Lower lane; siderophore isolated from cell free culture supernatant of different Xcc strains grown under low-iron condition (PS + 100 μM DP using Amberlite XAD-16 resin column chromatography. Cell normalized siderophore fractions were loaded on PSA-CAS plate wells. Strains: Xcc 8004 (wild-type strain), Δ*xibR* (*xibR* deletion mutant), Δ*xibR*Δ*xssA* [*xibR* and *xssA* (*X* *anthomonas* siderophore synthesis A) double mutant], and strains harboring the plasmid containing either the wild-type *xibR* allele (pSSP30) or a point mutant of *xibR* in the putative conserved aspartate residue phosphorylation site (D55AXibR; pSSP39), *xssA* (pAP15; wild-type *xssA* allele) and pHM1. Here onward all the experiments repeated three times until not mentioned. (B) Quantification of purified vibrioferrin using high-performance liquid chromatography (HPLC). Different Xcc strains were grown up to 1.0 X 10^9^cells/ml under low-iron condition (PS + 100 μM DP). Siderophore was purified from cell free culture supernatants using Amberlite XAD-16 resin columns. Cell normalized active fraction of siderophore was analyzed by HPLC. The vibrioferrin was detected at 300 nm and the concentration was determined while comparing the peak area (mUA × min) with standard curves generated from a known concentration of pure standard vibrioferrin. * indicate P < 0.05 in student’s *t* test (T-test). Error bars represent SD of the mean (n = 3). (C) Relative quantification of expression of the siderophore biosynthesis gene (*xssA*) of Xcc by real-time qRT-PCR. Different strains of Xcc; Xcc 8004, Δ*xibR* and Δ*xibR* harboring the plasmid containing the wild-type *xibR* allele (pSSP30), were grown to OD600 1.2 in PS, PS + 100 μM DP (low–iron condition), and PS + 100 μM DP + 100 μM FeSO_4_. 16S ribosomal RNA was used as an endogenous control to normalize the RNA for cellular abundance. * indicates P < 0.01 in Student’s *t* test. Standard errors were calculated based on at least three independent experiments. (D) Transcriptional analysis of *xssA* gene in the *X* *anthomonas* siderophore synthesis cluster. Expression analysis of *xssA* gene in the *X* *anthomonas* siderophore synthesis cluster in the wild-type (Xcc 8004 P*xssA*::*gusA*), Δ*xibR* (Δ*xibR* P*xssA*::*gusA*), and Δ*xibR* P*xssA*::*gusA* mutant harboring the complementing plasmid pSSP30 grown in PS medium containing 100 μM DP (low-iron condition). Error bars represent SD of the mean (n = 3) cell normalized Glucuronidase (GUS) activity represented as nanomoles of 4-methyl-umbelliferone (4-MU) produced per minute. * indicates P < 0.05 in Student’s *t* test, significant difference between the data obtained for the Δ*xibR* P*xssA*::*gusA* strain compared to those obtained from the parental strain Xcc 8004 P*xssA*::*gusA* and the complemented strain (Δ*xibR*/pSSP30 P*xssA*::*gusA*). (E) Expression analysis of P*xssA*::*gusA* in the wild-type strain (Xcc 8004 P*xssA*::*gusA*) harboring either the plasmid containing the wild-type *xibR* allele (pSSP30) or the empty vector (pHM1). Error bars represent SD of the mean (n = 3) cell normalized Glucuronidase (GUS) activity. * indicates P < 0.01 in Student’s *t* test, significant difference between the data obtained for the Xcc 8004 P*xssA*::*gusA*/pSSP30 strain compared to those obtained from the Xcc 8004 P*xssA*::*gusA*/pHM1 (vector control).

A genetic screen was performed by genome-wide Tn5-transposon mediated random mutagenesis to identify mutants altered in siderophore production in Xcc (See Supporting Materials and Methods; S1 Table; S1 Fig). We isolated three siderophore overproducing mutants (*xibRM2*, *xibRM1* and *xibRB1*) on petone–sucrose agar-chrome azurol sulphonate (PSA-CAS) plates containing 2,2′-dipyridyl (DP) that carried three independent transposon insertions in *xibR* (a *ntrC* family of response regulator; *XC*_3760) (S1B and S1C Fig; S1 Table and S2 Table). In addition, we also made an in-frame deletion in the *xibR* gene, Δ*xibR*, which also exhibited siderophore overproduction, similar to the transposon induced mutant strains (Fig 1A and 1B; S1D Fig; S2 Table;). Quantification of vibrioferrin siderophore isolated form the cell free culture supernatant of different strains of Xcc by Amberlite XAD-16 column chromatography and high performance liquid chromatography (HPLC) indicated that the Δ*xibR* mutant produced at least 4-fold more vibrioferrin than that produced by the wild-type Xcc 8004 strain (Fig 1B; S2 Fig). Complementation of the *xibR* mutants Δ*xibR*, *xibRM2*, *xibRM1* and *xibRB1* by *in trans* expression of the plasmid borne wild-type *xibR* allele (pSSP30) restored the wild-type levels of siderophore (Fig 1A and 1B; S1C and S1D Fig). As a control, the Δ*xibR*Δ*xssA* double deletion mutant strain [*xibR* and *xssA* (*X* *anthomonas* siderophore synthesis A)] failed to produce any detectable level of siderophore (Fig 1B and S1D and S1E Fig).

It has been reported that the aspartate at 54^th^ or 55^th^ residue of NtrC receiver domain position gets phosphorylated by the cognate sensor kinase (SK), which is required for the regulation of transcription of downstream genes [21–24]. Interestingly, *in trans* expression of a point mutant of *xibR* in the putative conserved aspartate residue phosphorylation site (D55AXibR; pSSP39) failed to rescue the siderophore overproduction phenotype of Δ*xibR* mutant (Fig 1B and S1D Fig).

In an attempt to understand the role of iron and XibR mediated regulation of siderophore biosynthesis, we performed expression analysis of *xssA* by real-time qRT-PCR and chromosomal reporter fusion P*xssA*::*gusA* in the wild-type Xcc 8004, Δ*xibR* and Δ*xibR* mutant harboring the complementing plasmid (Δ*xibR*/pSSP30) strains grown in PS, low-iron (PS + DP) and low-iron medium supplemented with FeSO_4_ (Fig 1C and 1D; S3 Fig). Expression of *xssA* was approximately 3-fold higher in the Δ*xibR* mutant compared to the wild-type Xcc 8004 strain grown under low-iron condition. Complementation of the Δ*xibR* mutant by *in trans* expression of wild-type *xibR* or exogenous supplementation of FeSO_4_ suppressed the expression of *xssA* (Fig 1C and 1D; S3 Fig). Notably, *in trans* expression of the multicopy plasmid borne wild-type *xibR* allele suppressed the expression and production of siderophore in the wild-type strain compared to the empty vector pHM1 which had no effect on siderophore production (Fig 1B and 1E; S1D Fig).

Interestingly, expression analysis of *xibR* by real-time qRT-PCR and chromosomal reporter fusion P*xibR*::*gusA* indicated that the *xibR* is induced under iron-replete conditions in the wild-type Xcc 8004 (S4 Fig).

### The Xcc XibR and NtrC are two functionally distinct members of the NtrC family proteins

**Fig 2:**
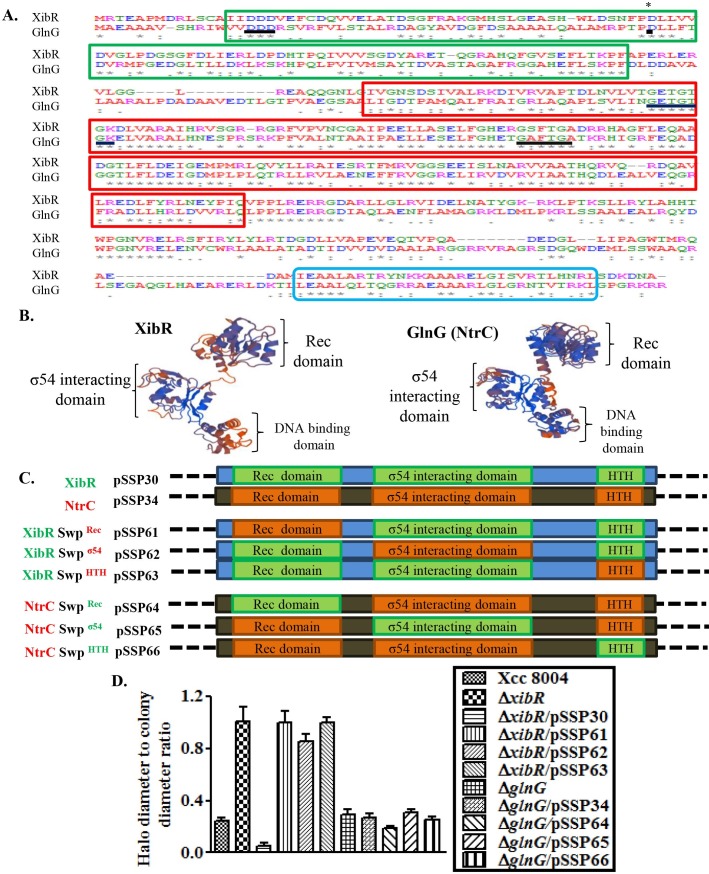
The Xcc XibR and NtrC are two functionally distinct homologs of NtrC family proteins. (A) Multiple sequence alignment of XibR and NtrC of Xcc. Sequence alignment was performed by using CLUSTALW. Asterisks indicate identical amino acids; (:) indicate highly conserved and (.) less conserved. (*) indicate the putative conserved aspartate residue phosphorylation site (D55). Region inside the green box indicate the N-terminal receiver (Rec) domain; region inside the red box indicate the central σ54 interacting domain or AAA+ domain; and the region inside the blue box indicate the C-terminal DNA binding domain or HTH domain Conserved motifs and amino acids are underlined. (B) Homology model of XibR and GlnG (NtrC) of Xcc 8004 showing structural similarity and both having N-terminal Rec domain, middle σ54 interacting domain and C-terminal DNA binding domain. Homology modeling was performed by using the SWISS-MODEL ProMod Version 3.70. (C) Schematic representation of XibR and GlnG (NtrC) domain swapped hybrid constructs in the plasmid vector pHM1. (1) pSSP30 (wild-type *xibR* allele in pHM1; XibR), (2) pSSP34 (wild-type *glnG* or *ntrC* allele; NtrC), (3) pSS61 (*xibR* with the swapped Rec domain from NtrC; XibR Swp ^Rec^), (4) pSS62 (*xibR* allele with the swapped σ54 interacting domain from NtrC; XibR Swp ^σ54^), (5) pSS63 (*xibR* with the swapped DNA binding HTH domain from NtrC; XibR Swp ^HTH^), (7) pSS64 (*ntrC* with the swapped Rec domain from XibR; NtrC Swp ^Rec^), (8) pSS65 (*ntrC* with the swapped σ54 interacting domain from XibR; NtrC Swp ^σ54^), and (9) pSS66 (*ntrC* with the swapped DNA binding HTH domain from XibR; NtrCSwp ^HTH^). (D) Quantification of siderophore production. Average ratio of siderophore halo to colony diameter for different strains of Xcc grown on PSA-CAS-DP plate. Xcc strains: Xcc 8004 (wild-type), Δ*xibR* (*xibR* deletion mutant), Δ*glnG* (*glnG* deletion mutant), Δ*xibR*/pSSP30 (Δ*xibR* mutant harboring the plasmid containing the wild-type *xibR* allele; XibR), Δ*glnG*/pSSP34 (Δ*glnG* mutant harboring the plasmid containing wild-type *glnG* or *ntrC* allele; NtrC), Δ*xibR* (pSS61; XibR Swp ^Rec^), Δ*xibR* (pSS62; XibR Swp ^σ54^), Δ*xibR* (pSS63; XibR Swp^HTH^), Δ*glnG* (pSS64; NtrC Swp ^Rec^), Δ*glnG* (pSS65; NtrC Swp ^σ54^) and Δ*glnG* (pSS66; NtrC Swp^HTH^). Error bars represent SD of the mean (n = 3).

The XibR shares 32% identity and 47% similarity with NtrC from Xcc (*ntrC*; XC_0198) (Fig 2A). In Xcc the *ntrC* (XC_0198), also known as *glnG*, is present in the *gln* cluster along with the cognate sensor kinase NtrB or GlnL which are involved in the transcriptional regulation of genes involved in nitrogen metabolism [17]. In contrast, the XibR is an orphan regulator as it is not linked with a sensor protein. XibR and GlnG are members of the NtrC family proteins with both containing three characteristic functional domains; the N-terminal receiver (Rec) domain, the central σ54 interacting domain or AAA+ domain and the C-terminal DNA binding domain or HTH domain (Fig 2A and 2B). Sequence analysis and homology modeling of XibR of Xcc indicated that the Rec domain, σ54 interacting domain, and DNA binding domain exhibited 32%, 55% and 35% identities with the GlnG respectively. In addition, the aspartate at 15^th^, 16^th^, 17^th^ and 55^th^ positions in the Rec domain; GETGTGK and GAFTGA motifs of the σ54 interacting domain are well conserved between GlnG and XibR (Fig 2A and 2B). In order to address whether XibR and GlnG exhibit functional similarity, we made an in-frame deletion mutant of the Xcc *glnG* gene (Δ*glnG*) (S2 Table). GlnG act as a positive regulator of glutamine synthetase which enables bacteria to survive in the medium with arginine as a sole nitrogen source [25,26]. The Δ*glnG* mutant of Xcc did not over produce siderophore, however, exhibited growth deficiency in minimal medium containing arginine as a sole nitrogen source, a phenotype, that can be rescued by *in trans* expression of the wild-type *glnG* allele (Fig 2D; S5 Fig). In contrast, the Δ*xibR* mutant did not exhibited growth deficiency on minimal medium containing arginine as a sole nitrogen source (S5B Fig). Further to investigate the functional overlap between the conserved domains in XibR and NtrC, we made domain swapped variants of XibR and NtrC (GlnG) (Fig 2C). All three strains of Δ*xibR* harboring the swapped domain from GlnG failed to complement for siderophore overproduction (Fig 2D and S5A Fig). Similarly, all three strains of Δ*glnG* harboring the swapped domain from XibR failed to complement the growth deficiency in minimal media having arginine as sole nitrogen source (S5B Fig). *In trans* expression of domain swapped variants of XibR and NtrC allele failed to rescue the Δ*xibR* and Δ*glnG* mutant phenotypes (Fig 2D; S5 Fig).

Furthermore, phylogenetic analysis at NCBI database using the UPGMA method after amino acid sequence alignment of XibR homologs with ClustalW and phylip 3.67 (mobyle.pasteur.fr/cgi-bin/portal) suggested that XibR is conserved among several members of the *Xanthomonas* group of phytopathogens. In addition, homologs of XibR are present in the other bacteria such as *Pseudoxanthomonas dokdonensis* (Psedo; KRG68042), *Lysobacter* sp. URHA0019 (Lyso; WP_027082117); *Bordetella bronchiseptica* (Bor; WP_003811339) (S6 Fig).

### Genome-wide expression analysis of the iron-starvation stimulon and XibR regulon in Xcc

To gain insight into the role of iron and/or XibR in regulating global gene expression in Xcc, we performed microarray-based gene expression analysis using the Agilent 8x15k array based on the sequenced strain of Xcc 8004 (See Materials and Methods). Genes regulated by iron-starvation and/or XibR were identified by comparing the gene expression in the wild-type Xcc 8004 strain grown under iron-replete and low-iron condition and comparing the Δ*xibR* mutant with the parental strain. Differentially expressed genes showing a log_2_ fold change of 1.5 in the two biological replicates were considered for further analysis. T-test p-value was calculated using volcano Plot (See Materials and Methods)).

**Fig 3:**
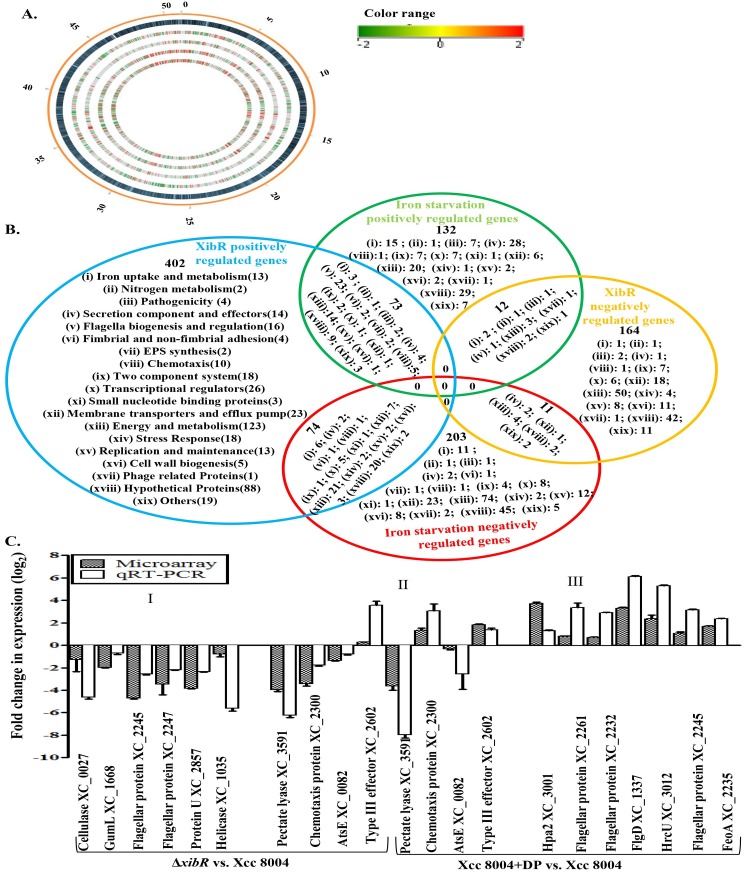
Genome-wide expression analysis of the iron-starvation and/or XibR regulon in Xcc. (A) The map of differentially expressed genes in response to iron limitation and/or *xibR* mutation in Xcc represented by using circos plot. From the outer to the inner circle, Track 1 shows circular genome of Xcc 8004 (~5.15Mb) with scale in Mb; Track 2, loci presentation of Xcc 8004 circular genome; Track 3, differentially expressed genes in Δ*xibR* mutant versus wild-type Xcc 8004 strain grown under iron-replete condition (PS medium); Track 4, differentially expressed genes in Δ*xibR* mutant grown under low-iron condition (PS + DP) versus iron-replete condition; Track 5, differentially expressed genes in Δ*xibR* mutant versus wild-type Xcc 8004 strain, both grown under low-iron condition; and Track 6, differentially expressed genes in the wild-type strain grown under low-iron condition versus iron-replete condition. Color scale indicates log_2_ –fold change of expression (from green for downregulated to red for upregulated). Low-iron was made by addition of 100 μM DP to PS medium. (B) Venn diagram showing the overlap and unique subset of genes belonging to different functional groups of Xcc whose expression is upregulated or downregulated under low-iron condition and/or XibR. For detail list of genes please see S3 to S10 Tables. (C) Expression analysis by microarray and real-time qRT-PCR indicating *xibR* and/or iron limitation regulated genes involved in flagellar biogenesis and regulation, metabolism, chemotaxis, and virulence. The y-axis represents log_2-_fold change in expression. For RT-PCR, data were normalized to an internal 16S rRNA control, and the relative changes in the transcriptional level were calculated as a ratio of transcript levels of Δ*xibR* versus wild-type Xcc 8004 strain grown in PS medium (iron-replete condition), and Xcc 8004 grown under low-iron condition (PS + DP) versus that grown in PS medium (iron-replete condition) using log_2_ of fold difference method. I, II and III represent set of genes which are affected by *xibR* only (I), influenced by both *xibR* and iron limitation (II) and genes affected by iron limitation only (III). Data represents the means ± S.E. (n = 3).

Analysis of transcriptional changes in response to low-iron condition in the wild-type Xcc 8004 and in the Δ*xibR* mutant strain indicated that both low-iron condition and/or *xibR* mutation had significant effect on genes expression in Xcc (Fig 3; S7 Fig). Differentially regulated genes were grouped under 19 major functional categories (see Materials and Methods; Fig 3B; S3–S10 Tables). The putative operons regulated by low-iron condition and/or XibR were predicted based on the Xcc 8004 genome sequence [27] and expression patterns seen in microarray analysis (S8 Fig).

Transcriptional response to low-iron condition indicated that iron starvation affected the expression of broad spectrum of genes in Xcc with a variety of functions [505 genes; 217 upregulated and 288 downregulated under low -iron condition; (Fig 3B)]. We identified 73 genes which were positively regulated by both XibR and low-iron condition (Fig 3B; S9 Table). Interestingly, many of these differentially regulated genes belonging to this group encode virulence associated functions such as flagellar biogenesis and regulation (23 genes), secretion components and effectors (4 genes), and chemotaxis (5 genes) (S9 Table). We also identified 12 genes which were repressed by both XibR and low-iron (Fig 3B; S10 Table). 132 genes were induced under low-iron condition but were not affected by the *xibR* mutation (Fig 3B; S5 Table). This group of genes included 15 iron uptake and metabolism related genes, 7 pathogenicity associated genes, 28 secretion components and effectors, and 7 two component systems associated genes (Fig 3B; S5 Table). Similarly, 203 genes, which included several bacterioferritins (iron storage proteins), were repressed under low-iron condition but not in the Δ*xibR* mutant (Fig 3B; S6 Table). Notably, 74 genes, which includes iron storage (bacterioferritins like proteins) and iron receptor encoding genes were positively regulated by XibR but repressed under low-iron condition (Fig 3B. S7 Table). Similarly, 11 genes were negatively regulated by XibR but activated by low-iron condition (Fig 3B. S8 Table). 402 genes were positively regulated by XibR but were not affected by low-iron condition (Fig 3B; S3 Table). Several of these genes which were positively regulated by XibR are involved in iron uptake/metabolism and virulence associated functions. These included 13 iron uptake and metabolism related genes, 4 pathogenicity associated genes, 14 secretion components and effectors, 14 flagellar and motility related genes, 4 attachment associated genes, 10 chemotaxis related genes and 18 two component systems associated genes, respectively (Fig 3B; S3 Table). Similarly, we also identified 164 genes which were repressed by XibR but not influenced by low-iron condition (Fig 3B; S4 Table).

We also performed quantitative real-time Reverse Transcriptase-Polymerase Chain Reaction (qRT-PCR) to confirm the pattern of expression of low-iron condition and/or XibR regulated genes, representing the set of genes which are affected by *xibR* only (Class I), influenced by both *xibR* and iron limitation (Class II) and genes affected by iron limitation only (Class III) performed real-time qRT-PCR for the low-iron condition and/or XibR regulated virulence associated factors to validate the microarray results. Real-time qRT-PCR of differentially expressed class I-III genes corroborated the microarray results (Fig 3C).

### Δ*xibR* mutant of Xcc exhibit altered ferric iron uptake and storage

**Fig 4:**
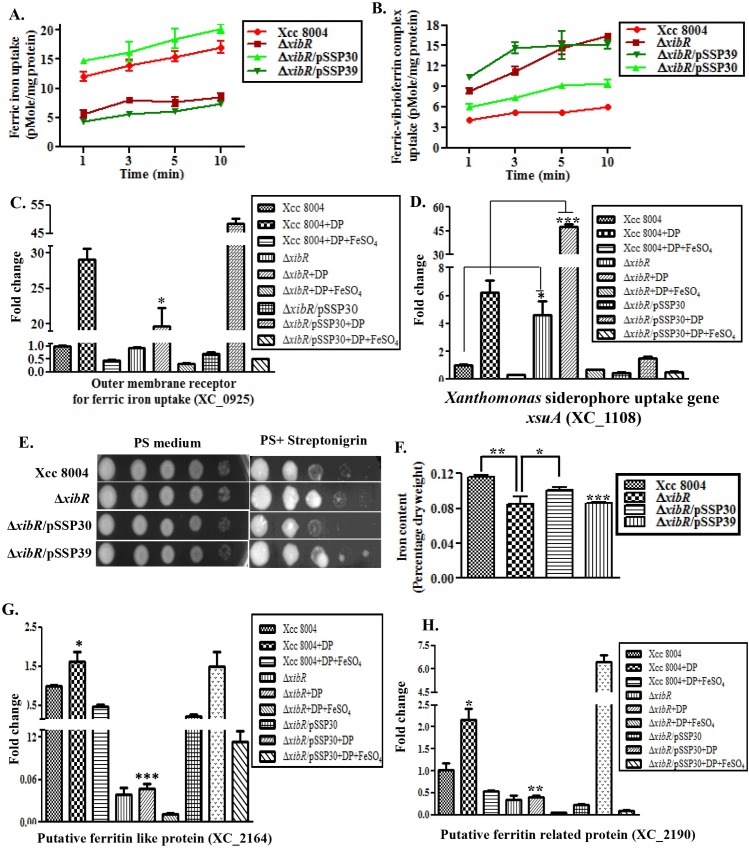
Δ*xibR* mutant of Xcc exhibit altered ferric iron uptake and defect in iron storage. (A) Δ*xibR* mutant exhibits defect in ferric iron uptake. Transport was initiated by addition of 0.5 μM ^55^FeCl_3_ to cell suspensions of Xcc 8004, Δ*xibR*, Δ*xibR*/pSSP30 and Δ*xibR*/pSSP39 grown under low-iron condition. Low-iron was made by addition of 150 μM DP to PS medium. Incorporation of radiolabelled Fe^3+^ was detected by scintillation counter. (B) Δ*xibR* mutant exhibits enhanced uptake of ferric iron-vibrioferrin complex. Transport was initiated by the addition of 0.5 μM 1:1 ratios of ^55^FeCl_3_ and vibrioferrin to the cell suspensions of different Xcc strains grown under low-iron condition. (C and D) Relative quantification of the expression of *Xanthomonas* siderophore uptake gene (*xsuA*) and outer membrane receptor for ferric iron (XC_0925) of Xcc grown under PS, PS + 100 μM DP, and PS + 100 μM DP + 100 μM FeSO_4_ by real-time qRT-PCR. 16S ribosomal RNA was used as an endogenous control to normalize the RNA for cellular abundance. (E) Streptonigrin (SNG) sensitivity plate assay. Different Xcc strains were grown in PS media at a density of 1 × 10^9^ cells/ml. 4 μL of cultures from each serial dilution was spotted on PSA plates containing 1μg/ml SNG and 0.01 M sodium citrate. Plates were incubated for 72 h at 28°C to observe bacterial growth. (F) Intracellular iron content quantification determined by atomic absorption spectrophotometry. Different Xcc strains were grown at a density of 1.2 OD_600_ in PS medium. Cells were harvested, freeze dried, and determined the iron content by Inductively Coupled Plasma-Optical Emission Spectrometry (ICP-OES). (G and H) Relative quantification of the expression of *Xanthomonas* putative ferritin-like protein (XC_2164) and putative ferritin related protein (XC_2190) of Xcc grown under PS, PS + 100 μM DP, and PS + 100 μM DP + 100 μM FeSO_4_ by real-time qRT-PCR. Data shown in the graphs are mean ± S.E. (n = 3). * Indicating p-value < 0.05, **indicating p-value < 0.01 and *** indicating p-value < 0.001 statistically significance by paired student t-test.

Analysis of our microarray data indicated that several genes related to iron storage (ferritin-like proteins), putative outer membrane receptor for ferric iron uptake (*fhuE*) and putative TonB dependent receptors were positively regulated by XibR (S3 Table; S9 Table). To understand the role of *xibR* in iron uptake and storage, we performed *in vitro* ^55^Fe^3*+*^, ^55^Fe^2+^ and ^55^Fe^3*+*^-vibrioferrin complex uptake assays with wild-type Xcc 8004, the *ΔxibR* mutant, *ΔxibR* mutant harboring the plasmid-borne wild-type *xibR* allele (*ΔxibR*/pSSP30), and the *ΔxibR* harboring the plasmid-borne point mutant D55AXibR allele (*ΔxibR*/pSSP39) as described previously [13,28,29] with few modifications (see Materials and Methods). The total amount of ^55^Fe^3+^ incorporated into the Δ*xibR* mutant or Δ*xibR*/pSSP39 was significantly less than that incorporated into the wild-type Xcc 8004 and the Δ*xibR*/pSSP30 over the 10 min time-course of the experiment (Fig 4A). Interestingly, uptake assay in the presence of vibrioferrin (1:1 ratio of ^55^Fe^3+^ and vibrioferrin) indicated that the total amount of ^55^Fe^3+^ incorporated was significantly higher in the Δ*xibR* or Δ*xibR*/pSSP39 strains compared to that incorporated into either the wild-type Xcc 8004 or Δ*xibR*/pSSP30 (Fig 4B). In contrast, there was no significant difference in the amount of radiolabelled Fe^2+^ incorporated into four of these strains (S9A Fig).

Reduced incorporation of radiolabelled ferric iron in the Δ*xibR* mutant compared to the wild-type strain indicated that XibR may be involved in the regulation of expression of low-affinity or non-vibrioferrin mediated ferric iron uptake or storage system/s under iron-deplete condition. Our microarray based expression analysis indicated that an outer membrane receptor for ferric iron uptake (*fhuE*; *XC*_0924), was positively regulated by low-iron and XibR (S9 Table). XC_0924 appears to be arranged in an operon with XC_0925, an outer membrane receptor for ferric iron uptake), which exhibit 49% identity and 67% similarity with previously reported FauA of human pathogenic bacteria *Bordetella pertussis* [30] and 47% identity and 68% similarity with FpvA of *Pseudomonas aeruginosa* [31], respectively. Real-time qRT-PCR analysis indicated that XC_0925 is also induced under low-iron condition and Δ*xibR* mutant exhibited reduced expression compared to the Xcc 8004 and Δ*xibR*/pSSP30 under iron-deplete condition (Fig 4C). In contrast, the expression of *xsuA* (*X* *anthomonas* siderophore uptake) was approximately 6-fold higher in the Δ*xibR* mutant compared to the wild-type Xcc 8004 and Δ*xibR*/pSSP30 strains grown either in iron-replete or under low-iron conditions, respectively (Fig 4D). We did not observe any significant difference in the expression of *feoB* (ferrous iron transporter) and ferrous uptake regulator *(fur*) in the Δ*xibR* mutant compared to the wild-type Xcc 8004 and Δ*xibR*/pSSP30 strains by real-time qRT-PCR (S9B and S9C Fig).

We next performed streptonigrin sensitivity assay, which depends on the intracellular iron levels, to assess intracellular iron content in different strains of Xcc [13,32]. Streptonigrin sensitivity assay indicated that the wild-type Xcc 8004 and Δ*xibR*/pSSP30 strains were hypersensitive to streptonigrin compared to the Δ*xibR* and Δ*xibR*/pSSP39 (Fig 4E; S9D Fig), indicative of low intracellular iron level in the Δ*xibR* mutant. In addition, measurement of intracellular iron content in different strains of Xcc by Inductively Coupled Plasma-Optical Emission Spectrometry (ICP-OES) indicated that the Δ*xibR* and Δ*xibR*/pSSP39 strains contained less intracellular iron (approximately 26%) compared to either the wild-type Xcc 8004 or Δ*xibR*/pSSP30, respectively (Fig 4F). Expression analysis by real-time qRT-PCR indicated that iron storage-related putative ferritin genes *XC_2164*, *XC_2190* and *XC_3752* were down regulated in the Δ*xibR* mutant compared to the in wild-type Xcc 8004 and Δ*xibR*/pSSP30 under low-iron condition (Fig 4G and 4H; S9H Fig). Growth assays under low-iron conditions indicated that the Δ*xibR* and Δ*xibR*/pSSP39 strains exhibited reduced growth compared to either the wild-type Xcc 8004 or Δ*xibR*/pSSP30, which could be rescued by exogenous iron supplementation (S9E–S9G Fig; S11 Table).

Since Δ*xibR* mutant exhibited lower level of intracellular iron content and growth deficiency under low-iron condition, it follows that some of the genes identified in our expression analysis by microarray and real-time qRT-PCR may have a role in iron metabolism. In order to examine this, we made three deletion strains; 1. Δ*yciE* Δ*yciF* Δ*XC_*3754, triple deletion mutant strain with the deletion of entire cluster of bacterioferritin-related protein encoding genes *XC_3752*, *XC_3753* and *XC_3754*); 2. Δ*fhuE* Δ*XC_0925*, double deletion mutant in the receptors for non-vibrioferrin mediated ferric iron uptake encoding genes (*XC_0924* and *XC_0925*); and 3. Δ*fecR* (*XC*_0057) encoding the periplasmic iron dicitrate sensor [33]. The bacterioferritin-related protein encoding genes (*XC_3752*, *XC_3753* and *XC_3754*) and the genes encoding the ferric iron uptake proteins (*XC_0924* and *XC_*0925) are regulated by both *xibR* and low-iron condition, whereas, the expression of *fecR* is only influence by low-iron condition (S5 Table. S9I Fig). Streptonigrin sensitivity and growth assays under low-iron condition indicated that the triple deletion strain Δ*yciE* Δ*yciF* Δ*XC_3754* and the Δ*fecR* mutant exhibited lower intracellular iron levels compared to the wild-type strain, whereas there was not much significant difference in the streptonigrin sensitivity and growth under low-iron condition in the Δ*fhuE* Δ*XC_0925* double mutant compared to the wild-type Xcc 8004 strain (S9J–S9N Fig; S11 Table)

### XibR and low-iron condition induces chemotaxis and motility in Xcc

**Fig 5:**
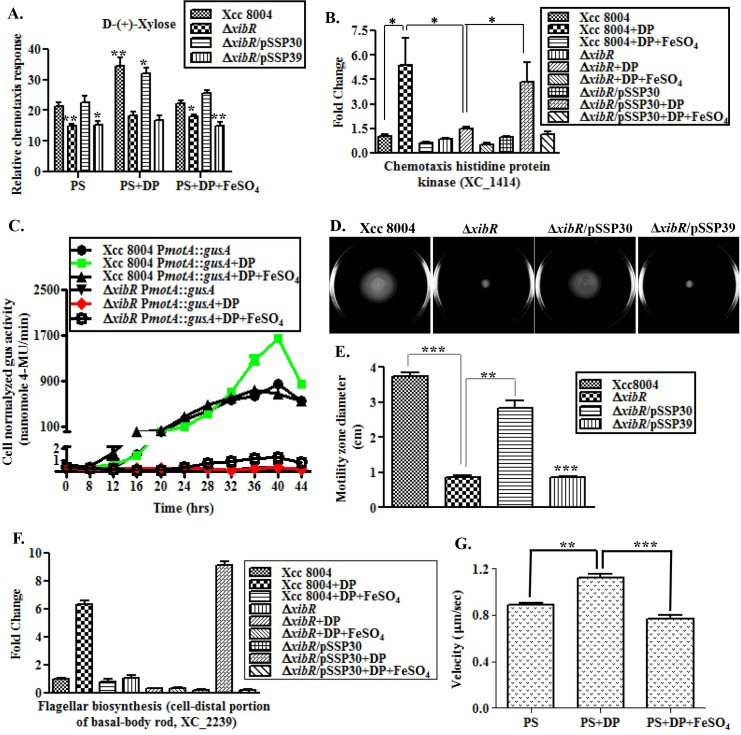
Chemotaxis and motility are regulated by *xibR* and induced under iron limitation. (A) Quantitative chemotaxis capillary assay in response to D-(+)-Xylose with different Xcc strains grown under PS, PS + 100 μM DP and PS + 100 μM DP + 100 μM FeSO_4_. Cells were incubated at 28°C with capillaries containing D-(+)-Xylose (1.2 mM) and PBS. Relative chemotaxis response was determined by migrated bacterial cells in capillary containing D-(+)-Xylose over the migrated bacterial cells in capillary containing PBS. Data are shown as mean ± S.E. (n = 3). The experiment was repeated two times. (B) Relative quantification of the expression of chemotaxis histidine protein kinase (cheA3) of Xcc grown under PS, PS + 100 μM DP, and PS + 100 μM DP + 100 μM FeSO_4_ by real-time qRT-PCR. 16S ribosomal RNA was used as an endogenous control to normalize the RNA for cellular abundance. (C) Expression analysis of *motA* operon in wild-type Xcc 8004 and Δ*xibR* mutant grown under PS, PS + 100 μM DP, and PS + 100 μM DP + 100 μM FeSO_4_ by monitoring the β-glucuronidase (GUS) activity. (D) Swim plate motility assay for different Xcc strains; Xcc 8004, Δ*xibR*, Δ*xibR*/pSSP30 and Δ*xibR*/pSSP39. (E) Motility zone diameter quantification from semisolid swim plate motility assay. (F) Relative quantification of the expression of flagellar biosynthesis gene for a cell-distal portion of basal-body rod (XC_2239) of Xcc grown under PS, PS + 100 μM DP, and PS + 100 μM DP + 100 μM FeSO_4_ by real-time qRT-PCR. (G) Bacterial velocity measurement from the live cell imaging of bacterial movement at single cell level by using manual tracking and chemotaxis tools with ImageJ software. Cells were grown in PS, low-iron (PS + 100 μM 2,2′-dipyridyl) and PS + 100 μM 2,2′-dipyridyl + 100 μM FeSO_4_ at 28°C up to mid-exponential phase, stained with Syto9 and incubated for 10 min at 28°C. The stained cells were loaded into a chamber of sterile glass bottom plates containing PS medium with 0.3% agar and visualized on epifluorescence microscope. Values are mean of at least 25 bacteria up to 20 frames. The experiment was repeated three times. Error bars are SEM. Data shown in the graphs are mean ± S.E. (n = 3).* Indicating p-value < 0.05, **indicating p-value < 0.01 and *** indicating p-value < 0.001 statistically significance by paired student t-test.

Expression analysis by microarray and real-time qRT-PCR indicated that several chemotaxis and motility-related genes are positively regulated by XibR and low-iron condition (Fig 3; S10A Fig; S3 and S9 Tables). It has been reported that *Xanthomonas oryzae* pv. *oryzae*, a member of the *Xanthomonas* group of phytopathogen, exhibits chemotaxis towards chemo attractant xylose and glutamic acid [34]. We performed quantitative chemotaxis capillary assay with Xcc 8004, Δ*xibR*, Δ*xibR*/pSSP30 and Δ*xibR*/pSSP39 strains grown either in PS medium or under low-iron condition. Analysis of relative chemotaxis response (RCR), which corresponds to the ratio of the number of bacteria in the test capillary over bacteria in the buffer control for each respective strain indicated that the Δ*xibR* and Δ*xibR*/pSSP39 strains exhibited significantly less chemotactic movement towards D-(+)-xylose and potassium glutamate compared to the Xcc 8004 and Δ*xibR*/pSSP30 strain (Fig 5A; S10B Fig). Interestingly, the wild-type Xcc 8004 strain grown under low-iron condition exhibited significantly higher RCR in response to xylose and glutamic acid, compared to those grown in PS medium. In contrast, the Δ*xibR* mutant did not exhibit induced chemotactic response when grown under low-iron condition (Fig 5A; S10B Fig). Real-time qRT-PCR revealed that the expression of chemotaxis histidine protein kinase (XC_1414), a homolog of CheA of *E*. *coli* [35], is induced under low-iron in the wild-type Xcc 8004 and Δ*xibR*/pSSP30, whereas there was no induction in the Δ*xibR* background (Fig 5B). Expression analysis using the chromosomal reporter fusion (P*motA*::*gusA*) harboring transcriptional fusion of *gusA* reporter gene downstream to the putative promoter of chemotaxis and motility-cluster (Fig 7A and S10A Fig) containing *motA*, *motB*, *cheW*, *cheY1* and *cheA1* genes, indicated that the expression of *motA* cluster was drastically reduced in the Δ*xibR* strain compared to the wild-type Xcc 8004 strain, wherein, the expression was further induced under low-iron condition (Fig 5C). Swimming motility assay and expression analysis of flagellar genes (*flgD*, *flgG*, XC_2239; S9 Table) indicated that XibR is required for motility and induced expression of flagellar component under low-iron condition in Xcc (Fig 5D–5F; S10C and S10D Fig). Next we wanted to examine whether low-iron condition promotes motility in Xcc. Since addition of iron specific chelator 2,2′-dipyridyl caused reduced growth in the swim plates, we performed live cell imaging with the wild-type Xcc 8004 cells grown in liquid culture under iron-replete and iron-deplete condition and stained with Syto9 (see Materials and Methods; [36]). We observed approximately 21% induction in Xcc 8004 movement under iron-deplete condition which was suppressed (approximately 32%) after supplementation of FeSO_4_ [Fig 5G; Supplementary videos SV1 (Xcc + PS), SV2 (Xcc + DP) and SV3 (Xcc + DP + FeSO_4_)].

### XibR is required for attachment and biofilm formation in Xcc

**Fig 6:**
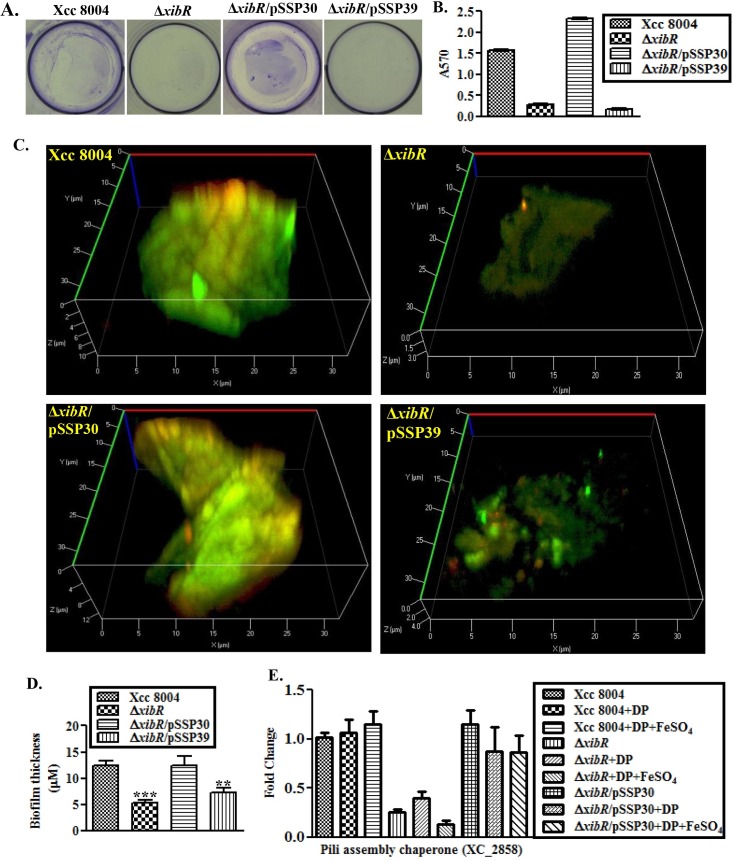
*xibR* promotes Biofilm formation. (A) Biofilm formation by Xcc 8004, Δ*xibR*, Δ*xibR*/pSSP30 and Δ*xibR*/pSSP39 strains in the static biofilm after 24 hrs of growth and staining with 0.1% Crystal Violet. (B) Quantification of attached cells of different Xcc strains in the static biofilm after 24 hours of growth. Attached cells were stained with Crystal Violet (CV), dissolved in ethanol and quantified by measuring absorbance at 570 nm. Data are shown as mean ± S.E. (n = 3). (C) Representative confocal laser-scanning microscopy (CLSM) images of biofilms formed on glass slides at the air–media interface by different Xcc strains grown in PS medium for 24h, and stained with BacLight LIVE/DEAD stain. Each 3D image represents the layer in the Z-stack. (D) Average biofilm thickness of different strains of Xcc formed on the glass slide at the air-media interphase. For quantification of the thickness, five independent biofilms were scanned with CLSM at ten randomly selected positions and thickness was determined through height of the biofilm. Data are shown as mean ± S.E. (n = 3). ** Indicating p-value < 0.01 and *** indicating p-value < 0.001 statistically significance by paired student t-test. (E) Relative quantification of the expression of pili assembly chaperone of Xcc grown under PS, PS + 100 μM DP, and PS + 100 μM DP + 100 μM FeSO_4_ by real-time qRT-PCR. 16S ribosomal RNA was used as an endogenous control to normalize the RNA for cellular abundance. Data are shown as mean ± S.E. (n = 3).

We observed that the Δ*xibR* mutant and Δ*xibR*/pSSP39 (D55AXibR) exhibited a more dispersed phenotype in broth culture compared to either the wild-type Xcc 8004 or Δ*xibR*/pSSP30 (S11A Fig). Further, we performed quantification of bacterial cells attached to polystyrene culture plates after 24 hours of inoculation by crystal violet (CV) staining, as described previously [34]. Quantification of biofilm formation by CV staining indicated that the Δ*xibR* strain exhibited significant defect in biofilm formation/attachment (Fig 6A and 6B). We also analyzed biofilm formation by different strains of Xcc by confocal laser-scanning microscopy using BacLight LIVE/DEAD bacterial viability staining, as described previously [37]. Analysis of the thickness of the biofilm formed using Z-projection of x-y stacks (optical sections) indicated that the Δ*xibR* and Δ*xibR*/pSSP39 formed biofilm with a considerable reduced thickness (approximately 2-fold less) compared to the wild-type Xcc 8004 strain or Δ*xibR*/pSSP30 (Fig 6C and 6D). However, we did not observe any significant difference in biofilm formation by Xcc 8004 and Δ*xibR* strains grown either in iron-deplete condition or in rich PS medium (S11B Fig). We identified a homolog of previously reported *E*. *coli* pili assembly chaperone PapD [38] in Xcc 8004 (XC_2858). Real-time qRT-PCR data indicated that XC_2858 was down-regulated in Δ*xibR* mutant compared to the wild-type Xcc 8004 and complemented strain Δ*xibR*/pSSP30. However, there was no significant difference under low-iron condition compared to the PS medium (Fig 6E).

### XibR regulates transcription of *mot*, *flg* and *xsuA* operons by binding to the promoters

**Fig 7:**
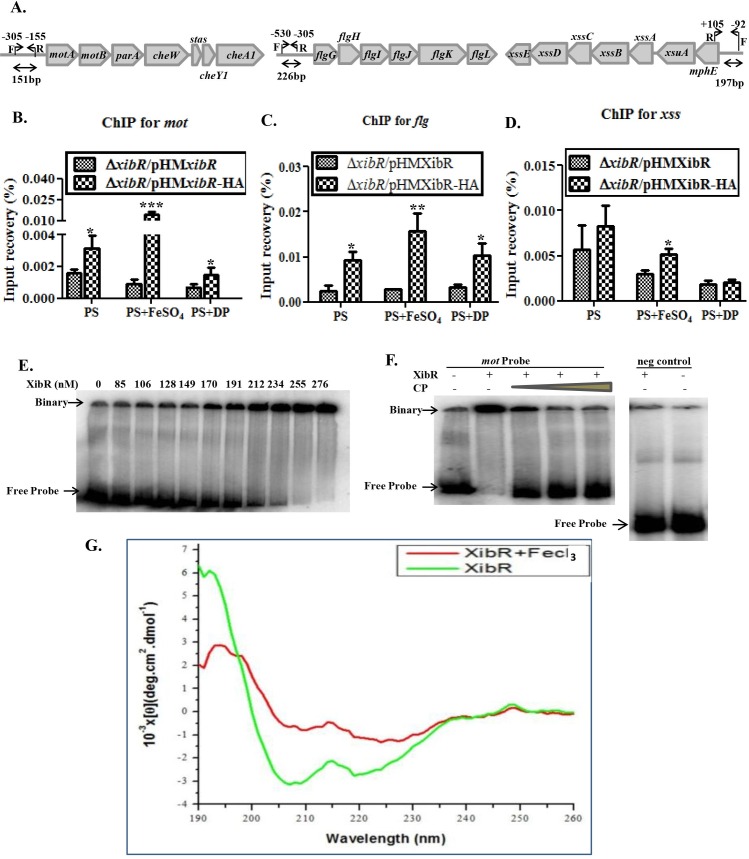
XibR binds to the upstream promoter region of *mot*, *flg* and xss operons. (A) Schematic representation of ChIP-qPCR primer locations (indicated by arrows) relative to the transcriptional start sites of *mot*, *flg*, and *xss* operons. (B, C, and D) ChIP-qPCR was performed to assess XibR occupancy on the upstream promoter region of *mot*, *flg*, and *xss* operons. Δ*xibR*/pSSP80 encodes full-length XibR with C-terminal HA-tag as a test and Δ*xibR*/pSSP30 encodes full-length XibR without tag as ChIP control grown in rich PS medium, iron-replete (PS + 100 μM FeSO_4_) and iron-deplete (PS + 100 μM DP) media and then immunoprecipitated with anti-HA antibodies. Data are shown as mean ± S.E. (n = 3). *p-value < 0.05, **p-value < 0.01 an ***p-value < 0.001 indicated statistically significant difference than ChIP control strain by paired student t-test. (E) Electrophoretic mobility shift assay (EMSA) showing binding of XibR to a ^32^P-labeled *motA* (-525 to +28) probe. More DNA-protein binary complex was observed while increasing the concentration of XibR. (F) Cold probe competition with unlabelled *motA* and non-specific DNA probe. Specific binding is indicated by a loss of XibR binding to the radiolabelled probe in the presence of excess of cold probe (indicated by CP). In the presence of a nonspecific probe (negative control), XibR did not exhibit binding. (G) Circular dichroism spectrum of XibR in absence or presence of 250 μM FeCl_3_. Y-axis indicates molar ellipticity.

Our expression analysis indicated that XibR is involved in the regulation of *mot*, *flg*, and *xss* operons. To determine whether XibR is capable of binding to the upstream region of *mot*, *flg*, and *xss* operons (Fig 7A), we performed chromatin immunoprecipitation (ChIP) using epitope-tagged XibR expressed in the Δ*xibR* strain and tagless XibR as ChIP control. Complementation experiment established that the HA-tag did not affect the function of the XibR protein (S12A Fig). The Δ*xibR*/pSSP80 (HA-tagged XibR) and Δ*xibR*/pSSP30 (tagless-XibR) strains were grown under iron-replete and iron-deplete conditions. DNA-protein cross-linking was done by formaldehyde treatment to bacterial cells followed by immunoprecipitation of XibR from lysates using anti-HA antibodies by a sandwich technique (see Materials and Methods). ChIP followed by quantitative real-time PCR (ChIP-qPCR) of captured DNA fragments indicated binding of XibR to the *motA* and *flg* upstream sequence (Fig 7B and 7C). Interestingly, XibR exhibited significant binding to *xss* promoter only under iron-replete condition (Fig 7D).

We also performed electrophoretic mobility shift assay (EMSA) to study the *in vitro* binding of XibR to *motA* promoter using the purified C-terminal His-tagged XibR (S2 Table; S12B–S12D Fig) and 553 bp upstream region of *motA* comparising the sequence from -525 to +28 (see Materials and Methods). EMSA analysis indicated that XibR specifically bind the radiolabelled *motA* fragment (Fig 7E and 7F). We also observed binding of XibR to the 393 bp upstream region of *xss* cluster comprising the sequence from -188 to +205 (S13A Fig). To rule out the possibility of probe shift due to DNA-protein aggregation, we performed EMSA with a non-specific ^32^P-labelled probe, which did not exhibit any shift (Fig 7F). Interestingly, we observed that the presence of ferric form of iron in the EMSA binding buffer promoted *in vitro* binding of XibR to DNA (S13B Fig). However, except for ferrous (Fe^2+^), presence of other metal ions did not affect the binding of XibR to the target DNA (S13C and S13D Fig). Since under aerobic conditions, ferrous iron is oxidized to ferric form of iron, and to rule out the possibility that the increased binding of XibR exhibited in the presence of ferrous iron may be due to conversion to ferric form, we added ferric specific chelator deferoxamine mesylate in the binding buffer. Addition of deferoxamine mesylate with ferrous form of iron drastically reduced the binding of XibR to the target DNA (S13C Fig).

In an attempt to identify putative consensus motif in the *xibR* regulated upstream regulatory sequence of *mot*, *flg* and *xss*, we performed consensus sequences search using the MEME (Multiple Em for Motif Elicitation; at http://meme-suite.org/tools/meme), which predicted five consensus sequence (S14 Fig; S12 Table). Sequence analysis of 200 bp upstream region of all the XibR regulated genes identified in the microarray experiment indicated that 19.29% of them harbor either of the consensus motif (motif 1 = 1.09%; 2 = 1.29%; 3 = 3.96%; 4 = 2.73%; and 5 = 10.23%) (S12 Table). It is possible that the consensus motif 5 (CAGAACGACAAC), which constitute 10.23% of the XibR regulated genes, could be the potential direct target of XibR.

Furthermore, we performed circular dichroism (CD) spectroscopy to detect possible structural changes in XibR in the presence of ferric iron. CD spectroscopy measurements suggested that iron elicited conformational changes upon binding to XibR that result in reduced content of α-helix in the protein (Fig 7G).

### *xibR* is required for optimum virulence

**Fig 8:**
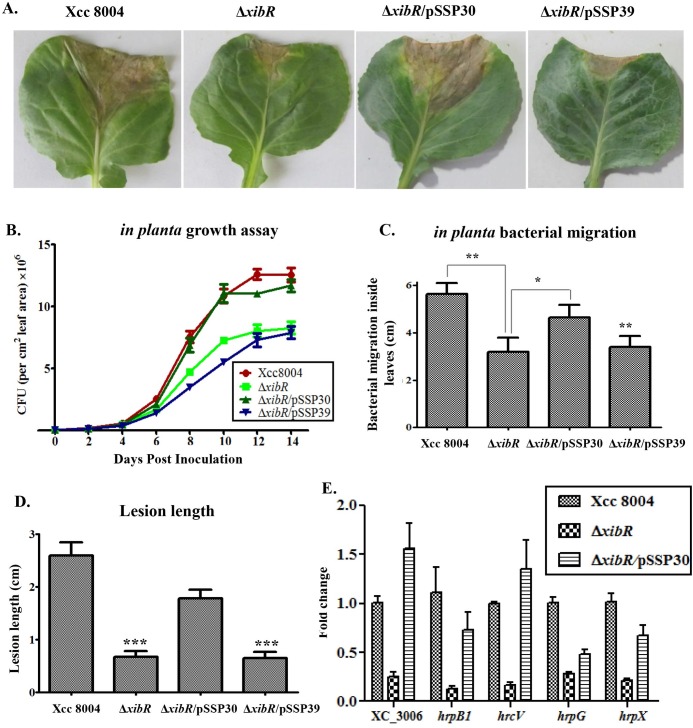
*xibR* is required for optimal virulence. (A) Infected cabbage leaves with different Xcc strain showing symptoms as a lesion at 15 days postinoculation. 30 days old plants were inoculated with bacterial cultures (1 X 10^9^ cells/ml suspension) of different Xcc strains by clip method. (B) *In planta* growth assays of Xcc 8004, Δ*xibR*, Δ*xibR*/pSSP30 and Δ*xibR*/pSSP39 strains. Bacterial populations were measured by crushing the leaves of 1cm^2^ areas for each and serial dilution plating at the indicated post inoculation days. Data are shown as mean ± S.E. (n = 3). (C) *In planta* bacterial migration assay was performed by inoculating 1cm pieces of infected leave, cut from base to tip with sterile scissors on rich PS medium with respective antibiotics. Migration was estimated by observing colonies formed after 1 to 3 days by the bacterial ooze from the cut ends of cabbage leaf pieces. (D) Quantification of lesion length at 15 days post inoculation. Data shown as mean ± S.E. (n = 25). (E) Relative quantification of the expression of different Type III secretion system *hrp* genes of Xcc 8004, Δ*xibR*, and Δ*xibR*/pSSP30 strains by real-time qRT-PCR. * Indicating p-value < 0.05, **indicating p-value < 0.01 and *** indicating p-value < 0.001 statistically significance by paired student t-test.

To understand the role of *xibR* in the virulence of Xcc, we performed infection studies with wild-type Xcc 8004, Δ*xibR*, Δ*xibR*/pSSP30 and Δ*xibR*/pSSP39 strains on cabbage plant. For infection studies, 30-day old cabbage leaves were inoculated with bacterial cell suspension by leaf clip method and monitored the lesion development, bacterial growth and migration inside the leaves (Fig 8). Infection studies suggested that the Δ*xibR* and Δ*xibR*/pSSP39 strains exhibited significant reduction in lesion development, growth and migration inside the leaves compared to either the wild-type Xcc 8004 or Δ*xibR*/pSSP30 (Fig 8A–8D) strain. Expression analysis by real-time qRT-PCR indicated that virulence associated functions such as Type III secretion protein ATPase encoding gene (*XC_3006*), *hrpB1*, *hrcV*, *hrpG* and *hrpX*, were downregulated in the Δ*xibR* mutant compared to either the wild-type Xcc 8004 or the Δ*xibR*/pSSP30, respectively (Fig 8E).

## Discussion

Ability of the bacterial pathogen to respond and adapt to iron limiting condition inside host is essential to their virulence. Pathogenic bacteria utilize diverse and efficient iron uptake systems that enable them to scavenge various forms of iron from the environment under iron-restricted conditions. Due to essentiality of iron in bacterial growth and survival particularly in iron limiting environment and also the potential toxic effect due to iron overload, bacteria tightly regulate iron uptake, metabolism and distribution in response to environmental cues mediated by iron dependent regulators such as Fur and DtxR [4,7,9].

Fur and Fur-like homologs play an important role in regulating iron metabolism and virulence in many Gram-negative bacteria. Fur binds to ferrous (Fe^**2+**^) form of iron which acts as a co-repressor, and suppresses the expression of high affinity iron uptake system under iron-replete condition [7,9]. Fur also has been shown to act as an activator of virulence associated factors in bacterial pathogens such as *Neisseria meningitidis*, *Salmonella typhimurium* and *Helicobacter pylori* [9]. However, apart from Fur and DtxR type ferrous binding regulators, little is known about how iron regulated genes and virulence associated functions are fine-tuned and coordinately regulated by other iron responsive regulatory proteins.

In this study we showed that a novel NtrC family of response regulator, XibR binds to ferric form of iron and regulates the expression of several iron metabolism and virulence associated functions in important phytopathogen *Xanthomonas campestris* pv. *campestris* (Xcc). To our knowledge iron-responsive ferric binding regulator has not been reported in any bacteria.

Many bacterial pathogens sense iron depletion as a signal that they are inside the host that enhance the expression of a wide variety of virulence associated functions such as toxins [6,7]. Our genome-wide expression analysis of iron starvation stimulon in Xcc revealed that low- iron condition affects the expression of broad spectrum of genes with diverse functions [approximately 12% of the 4249 coding DNA sequence]. Apart from genes which generally encode functions involved in iron acquisition, iron storage and metabolism, a large set of genes were also regulated by low-iron condition which are involved in pathogenicity and metabolism. Interestingly, several Type III secretion components and effectors, Type II effectors (cellulase, lipase, Polygalacturonase) were induced under low-iron condition (Fig 3; S5 Table). Type III secretion system which is required for the delivery of effector proteins inside the host cell is important for pathogenicity of several plant and animal pathogenic bacteria [39,40].

*Xanthomonas* group of phytopathogens produces vibrioferrin type of siderophore (xanthoferrin) [12–14]. However, the contribution of vibrioferrin siderophore in virulence appears to be variable among xanthomonads. For example, it has been shown that siderophore production is not essential for virulence in *Xanthomonas oryzae* pv. *oryzae* (a xylem vessel colonizing pathogen of rice). In contrast, the vibrioferrin production is required for optimum virulence of *Xanthomonas oryzae* pv. *oryzicola* (a parenchyma colonizing rice pathogen). It has been shown in *Xanthomonas* that under iron-replete condition, Fur suppresses the production of siderophore [16]. Our expression analysis of siderophore biosynthetic cluster (*xss*), siderophore production and ChIP assays strongly suggest that XibR repressed the expression of Xcc siderophore biosynthetic cluster (*xss*). It is possible that both iron bound form of XibR along with Fur mediates a tight regulation of high affinity siderophore mediated iron uptake under iron-replete conditions. Interestingly, supplementation of exogenous iron could abrogate the siderophore overproduction phenotype of the *xibR* mutant, which is in contrast to the Fur mutant of *Xanthomonas*, wherein, the addition of surplus exogenous iron could not abrogate the siderophore overproduction defect (S15 Fig) [16]. These results, and in addition to the fact that the *xibR* mutants exhibit growth deficiency under low-iron condition and deficient in production of iron storage proteins, suggest the dual role of XibR in regulating iron metabolism. Similar to Fur, XibR, act as a suppressor or negative regulator for the expression of siderophore biosynthetic genes under iron-replete condition, and act as a positive regulator of iron storage and ferric iron uptake genes such as ferritin, outer membrane receptor for ferric iron uptake (XC_0924, 0925), and several TonB dependent iron receptors (Fig 3; Fig 4; S9 Fig). This dual mode of regulation of iron uptake and metabolism genes by XibR might enable the cells to fine tune components of iron metabolism to changing iron availability in the environment. It is pertinent to note that recent studies in other pathogenic bacteria indicate that apo-Fur can act as an activator for certain iron transporters, in addition to its known role as a general repressor for other iron uptake and utilization functions [41–43]. Recent studies of Fur mediated regulation of iron uptake and metabolism in several bacteria including *E*. *coli* has shown complex regulatory roles beyond iron metabolism to coordinate complex cellular process. Fur bound with or without iron can act as a repressor as well as activator for different set of genes, which is in contrast to the classical repressor role reported for iron uptake and metabolism [9,44]. In our study, we have shown that low-iron condition induces motility and chemotaxis functions in Xcc, which are positively regulated by XibR (Fig 5; S10 Fig). It has been shown that motility and chemotaxis plays an important role in the host-pathogen interaction in several animal and plant pathogenic bacteria, and in many pathogens, motility is essential in some phases of their life style and that virulence and motility are often closely linked by complex regulatory networks [45–49]. In *Xanthomonas* group of phytopathogens, chemotaxis driven motility plays an important role in the virulence and has been implicated for the entry of the pathogen inside the host through openings known as hydathodes [34,50]. Furthermore, in *Xanthomonas campestris* pv. *campestris*, it has been shown that genes involved in flagellar biogenesis, chemotaxis and iron uptake and metabolism (ferric iron uptake) are required for optimum virulence [14,50]. Interestingly, (XC_2234) *flgB*, XC_2298 (*motD*), XC_2241 (*flgI*), XC_2260 (*fliF*) genes, which are regulated by XibR (S3 Table; S9 Table), has been shown to be required for the virulence of Xcc [50].

**Fig 9:**
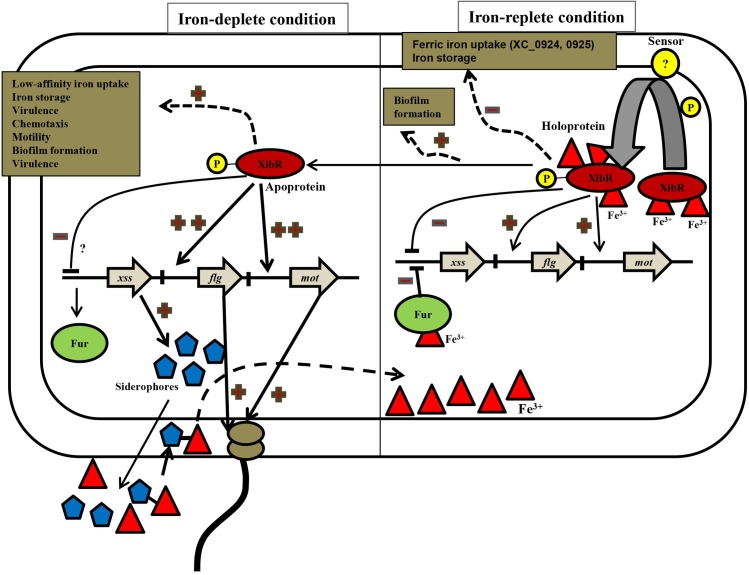
A proposed model for the role XibR in the regulation of iron homeostasis, chemotaxis, motility, biofilm formation, and virulence in Xcc. XibR is phosphorylated by a yet-unknown sensor kinase in response to change in environmental condition such as iron availability or host environment. Under iron-replete condition, holo-XibR (XibR-Fe^3+^) represses expression of *Xanthomonas* siderophore synthesis (*xss*) cluster along with Fur-Fe^2+^. XibR positively regulates chemotaxis and motility in Xcc. Under iron-deplete condition, wherein, the apo-XibR may be the predominant form in the cell, may act as a strong activator of motility and chemotaxis genes. The apo-XibR positively regulates expression of outer membrane receptors for ferric iron uptake, iron storage proteins (ferritin). XibR regulates the expression of several cellular functions such as biofilm formation and production of virulence associated functions (Type III effectors and regulators).

Notwithstanding to our *in vitro* electrophoretic mobility shift assay, which indicated requirement of ferric form of iron for binding of XibR to the *motA* upstream regulatory sequence, the *in vivo* ChIP experiments indicated binding of XibR to the *motA* and *flgG* upstream regulatory sequence either under iron-replete or iron-deplete condition. It may be possible that under *in vivo* condition, XibR bound with or without iron (apo or the holo form of XibR) can act as an activator of *motA* and *flgG* cluster. Under iron starvation condition, wherein, the apo-XibR may be the predominant form in the cell, it may act as a strong activator of motility and chemotaxis genes (Fig 9).

In this study, we have shown that iron starvation induces the expression of several genes involved in motility, chemotaxis and functions involved in iron metabolism. Importantly, XibR is involved in the regulation of several of these iron responsive genes. Furthermore, we have shown by EMSA and *in vivo* ChIP experiments that binding of XibR to the regulatory sequences of these virulence associated locus is affected by iron availability. These results strongly suggest the co-regulatory role of both iron and XibR in regulating virulence associated functions. XibR suppresses expression of siderophore biosynthesis and uptake genes under iron-replete condition and positively regulate several iron storage and putative low-affinity iron uptake genes under iron-deplete condition (Fig 1; Fig 4; S9 Fig). This indicates that XibR is involved in the fine-tuning the expression of components of iron metabolism in response to exogenous iron availability. We have proposed a model which describes the role of XibR in the regulation of iron metabolism and virulence associated function in Xcc (Fig 9). Under iron-replete condition, holo-XibR (XibR-Fe^3+^) represses expression of *Xanthomonas* siderophore synthesis (*xss*) cluster along with Fur-Fe^2+^. XibR positively regulates chemotaxis and motility in Xcc. Under iron-deplete condition, wherein, the apo-XibR may be the predominant form in the cell, may act as a strong activator of motility and chemotaxis genes. The apo-XibR positively regulates expression of outer membrane receptors for ferric iron uptake, iron storage proteins (bacterioferritin). XibR regulates the expression of several cellular functions such as biofilm formation and production of virulence associated functions (Type III effectors and regulators).

Recent studies of Fur regulon in *E*. *coli* and other pathogenic bacteria has indicated complex regulatory mode of Fur and iron in the regulation of diverse cellular functions, wherein, Fur can serve as a dual role of activator and repressor either in the presence or absence of iron [9,44]. In addition, Fur has been shown to have a dual regulatory role in the expression of common target gene. For example, in *E*. *coli* Fur has been shown to indirectly regulate the expression of aconitase (*acnA*) by suppressing the expression of small RNA RhyB which can lead to degradation of *acnA* under iron-replete condition in addition to its direct activation under iron-replete condition.

Comparison of iron starvation stimulon and XibR regulon indicated that although several functions are coordinately regulated by both XibR and low-iron condition, however, many of the functions are also uniquely regulated by both these factors. For example only 23% (170 of 736 genes) of the XibR-dependent genes are also influenced by iron starvation condition. Similarly, 33% of differentially expressed genes under low-iron condition are also regulated by XibR (Fig 3). This indicate that apart from iron and XibR, other environmental signals, or other iron-responsive transcription factors or additional transcription factors may play a role in the regulation of XibR and low-iron condition-dependent genes. Interestingly, several transcription factors (TFs) were differentially regulated by both XibR and under low-iron condition such as several LysR, AraC and TetR family of TFs. It is possible that these TF may be also involved in the regulation of expression of XibR and low-iron condition dependent genes to provide additional fine tuning of coordinated regulation of various cellular processes.

Interestingly, it has been reported that majority of the Fur-dependent genes were not directly regulated by Fur and has been proposed to be targets of indirect *fur* regulation, or other transcription factors responsive to variety of environmental condition including iron, as variation in iron availability can lead to diverse physiological changes [9,44]. This indicates that regulation of iron metabolism and coordination of different cellular functions associated with iron availability is mediated by complex regulatory network, which varies substantially in different bacterial species and may serve as an adaptation to suite different lifestyle. For example, it has been shown that under low-iron condition, apo-Fur and apo-IscR suppresses biofilm formation in *E*. *coli* [44,51]. In contrast, biofilm formation assay and expression analysis of pili assembly chaperon indicated that XibR positively regulates biofilm formation in Xcc (Fig 6).

Our *in vitro* EMSA assay and circular dichroism (CD) spectroscopy analysis suggest that XibR binds with ferric form of iron. Although ferrous form of iron is the predominant form which is involved in cellular metabolism, iron is mostly stored in the ferric form in storage protein such as ferritin, bacterioferritin [7]. It has been proposed that Fur, in addition to its role as an iron-responsive transcriptional regulator can have an additional function as ferrous iron buffer-storage protein [7]. It is possible that XibR which binds to ferric form of iron has an additional role of ferric iron storage, in addition to other ferric iron storage proteins and serve as an iron source under iron-replete condition.

In summary, we have identified a novel ferric iron binding transcription factor XibR, which belong to the NtrC family of protein in Xcc. XibR plays a dual role in iron metabolism, suppression of siderophore expression under iron-replete condition and positively regulates the expression of outer membrane receptors for iron/iron complex uptake. XibR and low-iron condition coordinately regulates several virulence associated functions such as motility, chemotaxis. We have shown that XibR binds to ferric form of iron which is in contrast to other iron responsive transcription factor which binds to ferrous form of iron. Our results reveal complex regulatory roles of iron and XibR beyond iron metabolism to coordinate complex cellular process.

## Materials and Methods

### Bacterial strains, plasmids and culture conditions

The bacterial strains and plasmids used in this study are listed in S2 Table. *Xanthomonas campestris* pv. *campestris* 8004 and *Xanthomonas oryzae* pv. *oryzae* strains were grown in 28°C in PS medium [52] and 200 rpm (New Brunswick Scientific, Innova 43, Edison, NJ, USA). The *E*. *coli* strains were grown in *Luria-Bertani* medium [53] at 37°C and 200 rpm. The concentrations of antibiotics were used were rifampicin (Rif; 50 μg/ml), spectinomycin (Spec; 50 μg/ml), kanamycin (Kan; 50 μg/ml), gentamycin (Gent; 5 μg/ml), tetracycline (Tet; 5 μg/ml) and nalidixic acid (Nal; 50 μg/ml). 2, 2′-dipyridyl (Fluka Analytical, Steinheim, Westphalia, Germany) was used as an iron chelator in low-iron medium.

### Molecular biology and microbiology techniques

Standard molecular biology and genetics related techniques including genomic DNA isolation, plasmid isolation and gel extraction were done as described previously [54] or by using kits provided by Qiagen (QIAGEN Inc., Valencia, CA, USA). PCR amplifications were performed with high-fidelity accutaq polymerase (Sigma-Aldrich, St. Louis, MO, USA) and Taq polymerase (Thermo Fisher Scientific, Waltham, MA, USA) as per manufacturer’s instructions. Restriction digestions and ligations were carried out with enzymes provided by New England Biolabs (Ipswich, MA, USA) as per manufacturer's instructions. Transformations were done by conjugation, electroporation or heat shock method. The oligos used in this study are listed in S13 Table.

### Generation of deletion strains

*In frame* deletion constructs were made as described previously [55] with few modifications. The 5’ and 3’ flanking regions of the gene to be deleted (approximately 300bp) were amplified, digested with one common inward restriction enzyme, ligated and cloned in suicidal vector pK18mobsacB. Subsequently, the deletion was accomplished by allelic exchange and homologous recombination while utilizing the suicide vector pK18mobsacB harbouring deletion construct of the gene of interest (see Supporting Materials and Methods).

### CAS plate assays for siderophore production

The siderophore production assays were done on CAS agar plates [56] with certain modifications [15]. Individual colonies of different *Xanthomonas* strains were spotted on the PSA-CAS plates, and were incubated at 28°C. CAS production assays under the low-iron condition was done by adding the ferrous iron chelator 75 μM 2, 2′-bipyridyl in PSA-CAS plates.

### Screen for isolating siderophore over-production mutants of Xcc

For screening siderophore overproducing mutants, 12,000 colonies from a transposon (Tn5; [57]) induced mutant library were grown on peptone sucrose agar (PSA)-chrome azurol sulfonate (CAS) siderophore indicator plates [56] containing 75 μM 2,2′-dipyridyl. Plates were incubated at 28°C for 24 h. Appearance of orange halo indicative of secreted siderophore production was scored by measuring the halo diameter (See supporting Materials and Methods for detail).

### Siderophore estimation from the cell-free culture supernatant of different strains of Xcc by HPLC

Siderophores estimation of different Xcc strains was done by the HPLC quantification of the purified vibrioferrin from cell-free culture supernatant as described previously [13,14] with few modifications. Briefly, different Xcc strains were grown up to the density of 1.0 X 10^9^cells/ml in rich PS medium with respective antibiotics. 0.2% of inoculum transferred to fresh PS medium (1 litre) supplemented with 100 μM 2, 2’-dipyridyl, grown to the density of 1.0 X 10^9^cells/ml. The bacterial cell cultures were centrifuged at 17000g for 50 min. Siderophore from the cell-free supernatants from different cultures were isolated by using Amberlite XAD-16 resin columns as described previously [58,59] with few modifications. Acidified supernatant with HCl up to pH 2 was allowed to pass through the prepared column (2.4 X 30 cm) and eluted with 200 ml of methanol. Flow-through was collected into 80 fractions and each fraction was tested for siderophores using CAS plate assay. Estimation of siderophores from concentrated positive fractions were carried out as described previously [59] with few modifications. We passed the samples through Agilent 1100 series HPLC system (Agilent, Santa Clara, CA, USA) and further data were recorded and processed by using chemstation software (Agilent 1100). The siderophore concentration was determined while comparing the peak area with the standard curves generated using a known concentration of a pure standard vibrioferrin (a kind gift provided by Masaki J Fujita, Hokkaido University, Japan).

### Gene expression profiling

Agilent 8x15K gene expression microarray was designed with probes having 60-mer oligonucleotides from NCBI transcript sequences of *Xanthomonas campestris* pv. *campestris* str. 8004 which comprised of a single circular chromosome comprising 5,148,708 bp nucleotides with 4332 predicted protein coding gene [27]. The 15K array comprised of a total number of 15,744 features including 15,208 probes, 536 Agilent controls. All the oligonucleotides were designed and synthesized in situ as per the standard algorithms and methodologies used by Agilent Technologies for 60-mer in situ oligonucleotide DNA microarray. Out of 4332 number of transcripts, 4234 probes were designed from NCBI Transcript sequences with an average of 1 probe per sequence. For 150 transcripts, additionally 2 probes were designed. The blast search analysis was performed against the respective sequence databases to check the specificity of the probes. Finally, 4560 probes were designed for NCBI Transcript sequences and specific probes were replicated to fill the blank spots. Total RNA was extracted from Xcc 8004 wild-type and Δ*xibR* mutant cultures at mid-exponential phase grown in either rich PS or low-iron media. Labelling, microarray hybridization, scanning and data analysis were performed as per manufacturer’s instruction (See supporting Materials and Methods for detail). Data extraction from Images was done using Agilent Feature Extraction software. Feature extracted data was analyzed using GeneSpring GX version 11software from Agilent. Normalization of the data was done in GeneSpring GX using the 75^th^ percentile shift and Normalization to Specific Samples. Samples were grouped based on the replicates. Genes that were significantly up regulated by 0.6 or more or down regulated by -0.6 or less fold (log_2_–fold change) were identified.T-test p-value was calculated using volcano Plot. Differentially regulated genes were clustered using hierarchical clustering to identify significant gene expression patterns (see supporting Materials and Methods). Differentially regulated genes were grouped under following 19 major functional categories: (i) iron metabolism, (ii) nitrogen metabolism, (iii) pathogenesis-related genes, (iv) secretion components, (v) flagella biogenesis and regulation, (vi) fimbrial and non-fimbrial adhesions, (vii) extracellular polysaccharides, (viii) chemotaxis, (ix) two-component system, (x) transcriptional regulators, (xi) small nucleotide binding protein, (xii) membrane protein transporters and efflux proteins, (xiii) energy and metabolism, (xiv) stress response, (xv) replication and maintenance, (xvi) cell wall biogenesis (xvii) phage-related genes (xviii) hypothetical proteins and (xix) others (Fig 3B; S3–S10 Tables). The annotation of the genes for functional categorization was done by conserved domain database of NCBI and protein data bank of RCSB. The un-annotated genes and the genes out of above functional categories were kept under others category.

### Expression analysis by real-time qRT-PCR

For total RNA isolation, different Xcc strains were grown in PS, low-iron (PS + 100 μM 2,2′-dipyridyl) and FeSO_4_ supplemented low-iron media (PS + 100 μM 2,2′-dipyridyl + 100 μM FeSO_4_) at 28°C and 200 rpm (New Brunswick Scientific) up to mid-exponential phase. Total RNA of cells was isolated by Trizol (Invitrogen, CA, U.S.A.) method as per the manufacturer’s instructions. Real-time qRT-PCR was done in 7500 Real-time PCR system (Applied Biosystems) and analyzed by SDS relative quantification software (Applied Biosystems, USA) as described previously [34,14]. Primers used for the real-time qRT-PCR are listed in supporting S13 Table.

### β-Glucuronidase reporter assays

Different strains of Xcc harboring the chromosomal transcriptional fusions with GUS reporter (S2 Table; see Supporting Materials and Methods) were grown in rich PS medium with appropriate antibiotics at 28°C and 200 rpm for overnight. 0.2% primary inoculum was transferred to the PS, low-iron (PS + 100 μM 2, 2′-dipyridyl) and FeSO_4_ supplemented low-iron (PS + 100 μM 2, 2′-dipyridyl + 100 μM FeSO_4_) media and grown at 28°C and 200 rpm in shaking incubator (New Brunswick Scientific, Innova 43, Edison, NJ, USA). At regular time intervals, absorbance was measured at 600 nm, cells were harvested from 1ml culture, washed with sterile miliQ water, resuspended in 250 μl of 1 mM MUG (4-methylumbelliferyl β-d-glucuronide) extraction buffer (50mM sodium dihydrogen phosphate [pH 7.0], 10 mM EDTA, 0.1% sodium lauryl sarcosine, 0.1% Triton X-100, and 10 mM β-mercaptoethanol) (Jefferson et al., 1987) and incubated at 37°C. Subsequently, reactions were terminated after addition of 675 μl of 0.2 M Na_2_CO_3_ into 75μl of reaction mixture and fluorescence was measured with 4-methyl-umbelliferone (MU; Sigma) as the standard at excitation 365nm and emission 455 nm of wavelength. β-Glucuronidase activity for GUS assays was expressed as nanomoles of MU produced/minute.

### GFP (Green Fluorescence Protein) reporter assays

For GFP activity, different strains of Xcc harboring the plasmid born GFP transcriptional reporter plasmids (S2 Table; Supporting Materials and Methods) were grown similar to the above mentioned β-Glucuronidase reporter assays. At different time points, 200 μl of culture was directly taken to measure GFP at excitation and emission wavelength of 472 nm and 512 nm respectively. Fluorescence was measured using hybrid microplate reader (Synergy H1 Hybrid Reader, BioTek, Winooski, VT, U.S.A).

### Chemotaxis assay by capillary method

We performed chemotaxis assay by the capillary method as described previously [34,60,61] with few modifications. Briefly, different Xcc strains were grown up to a density of 1.0 X 10^9^cells/ml in rich PS, low-iron (PS + 100 μM DP) and iron supplemented (PS + 100 μM DP + 100 μM FeSO_4_) media with appropriate antibiotics. Cells were harvested by centrifugation at 4000g (Sorvall RC-5B, Dupont) for 10 min, washed twice with sterile water and resuspended in PBS. Capillary tubes containing 200 μl of PBS, potassium glutamate (4.9 mM) and D-(+)-Xylose (1.2 mM) were incubated with cell suspension of different strains of Xcc at 28° C for 6 hours and then the content of the capillary was serially diluted in sterile miliQ water. The migrated bacterial cells in the capillary was determined by serial dilution and plating. The RCR (Relative Chemotaxis Response) was measured as the ratio of the bacteria that entered the test capillary over the number of bacteria entered the control capillary (PBS) for each respective Xcc strain.

### Motility assays

Swim plate assay was performed as described previously [34],. Briefly, Xcc strains were grown overnight in PS medium with appropriate antibiotics at 28°C, washed, centrifuged and resuspended in 1/10^th^ volume autoclaved miliQ water. 4μl of suspension was inoculated at the centre of swim plates (0.1% Agar containing PS medium) and incubated at 28°C for 2 days. The motility was quantified by measuring the diameter of swimming motility zone after 40 h.

### Microscopy assay for bacterial movement

Velocity measurement of moving bacterial cells was done as described previously [36] with modifications. Briefly, bacterial cells were grown in PS, low-iron (PS + 100 μM 2,2′-dipyridyl) and PS + 100 μM 2,2′-dipyridyl + 100 μM FeSO_4_) at 28°C and 200 rpm up to mid-exponential phase and stained with Syto9 [BacLight; Bacterial Viability Kit L7012; Invitrogen, Eugene, OR, U.S.A.). Syto9 (1:1000 v/v) was added into the culture, and incubated for 10 min at 28°C. The stained cells were loaded into a chamber of sterile glass bottom plates (Greiner Bio-one, Frickenhausen, Germany) having PS medium with 0.3% agar (iron-depleted and iron supplemented), for visualization. Live cell images were captured with a Nikon Inverted Microscope (Eclipse Model No. TI-DH; Tokyo, Japan) equipped with CCD camera mounted on an Axiophot epifluorescence microscope with a mercury excitation lamp. Green fluorescence of Syto9 stained bacterial cells were detected with an Endow filter set fitted with 488 nm excitation and 520 nm emission filters through a 100X oil objective. The captured digital images were saved in ND format and videos were made using NIS Elements AR 3.1 software. Videos were analyzed in ImageJ (https://imagej.nih.gov/ij/index.html) and velocities of the bacterial movement were calculated using the manual tracking plug-in and chemotaxis tools (https://imagej.nih.gov/ij/plugins/index.html).

### Iron uptake assay

^55^Fe uptake assay performed as described previously [28,29] with few modifications. Bacterial strains were grown overnight in PS medium with respective antibiotics and then 0.2% inoculum transferred in fresh PS medium containing 150 μM 2’2’-bipyridyl and grown for 24 h in 28°C shaking incubator at 200 rpm (New Brunswick Scientific). Further bacterial cells were harvested by centrifugation at 6,000 rpm for 5 min at 4°C and pellets were washed twice in 50 mM sodium phosphate buffer (pH-7.4). Bacterial cells were then resuspended in sodium 50 mM sodium phosphate buffer (pH-7.4). The bacterial suspensions were diluted with chelex-100 (Sigma) treated PS to an OD_600_ of 1.0 and incubated at 28°C for 5 minutes. Iron uptake was initiated by addition of 0.5 μM of ^55^FeCl_3_ of specific activity 10.18mCi/mg (American radiolabelled chemicals, Inc., St. Louis, USA). The stock solution was diluted ten times to with either water for the ^55^Fe^+3^ uptake studies or in 1M sodium ascorbate for ^55^Fe^+2^ uptake studies. Uptake assays of ferric iron-vibrioferrin complex were performed by using vibrioferrin (7.6 mM stock) and ^55^FeCl_3_ in 1:1 ratio. To stop the iron uptake, 200 μl of the cell suspensions were layered onto the 300 μl of di-butyl phthalate and di-octyl phthalate (1:1) mixture at different time points, immediately centrifuged at 14,000 rpm for 1.30 min and the pellets were resuspended in 100 μl of 1% (v/v) Triton X-100. The suspensions were transferred in scintillation vials having 5 ml of scintillation cocktail and counted in the ^3^H channel of scintillation counter (Perkin Elmer, Liquid Scintillation analyzer, Tri- Carb 2910 TR, USA).

### Streptonigrin sensitivity assay

For indirect quantification of intracellular level of iron, we performed streptonigrin sensitivity assay in broth culture as described previously [62]. Briefly, different strains of Xcc were grown to OD_600_ 1.0 in PS medium with respective antibiotics at 28°C and 200 rpm in shaking incubator. Cells were harvested by centrifugation at 4000 g for 6 min and pellets were diluted with fresh PS medium up to OD_600_ of 0.6. 100 μl of culture was then inoculated in 4 ml PS medium with streptonigrin (Sigma-Aldrich, St. Louis, USA) and sodium citrate and incubated in the static incubator at 28°C. OD_600_ was measured after 16 and 40 hrs of incubation.

### Estimation of intracellular iron

The intracellular iron content of different Xcc strains were measured by atomic absorption spectroscopy as described previously [29]. Briefly, different strains of Xcc were grown overnight up to a density of 1.0 X 10^9^ cells/ml in PS medium with respective antibiotic. Fresh PS medium (250 ml) was then inoculated with the overnight grown culture to a density of 1.0 X 10^4^ cells/ml. Cultures were grown to a density of 1.0 X 10^9^ cells/ml and cells were harvested by centrifugation, pellets were washed twice with phosphate buffer saline (PBS), lyophilized and dry weight was measured. Further, the lyophilized cells were dissolved in 30% HNO_3_ at 80°C for overnight and then diluted 10-fold with sterile miliQ water. The cellular iron content was determined by using ICP-OES (JY 2000 sequential ICP-OES spectrometer, Jobin Yvon, Horiba, France). Quantification of iron was performed against an aqueous standard of iron traceable to the NIST (National Institute of Standards and Technology, India).

### Biofilm assay

Different Xcc strains were grown in rich PS medium with required antibiotics at 28°C at 200 rpm (New Brunswick Scientific, Innova 43, Edison, NJ, USA) up to the 1.0 X 10^9^ cells/ml. Bacterial cells were harvested by centrifugation at 4000 g (Sorvall RC-5B, DuPont) and resuspended in fresh PS, low-iron (PS + 100 μM DP) and iron supplemented (PS + 100 μM DP + 100 μM FeSO_4_) media. Approximately 1.0 X 10^6^ cells/ml of secondary inoculum was transferred in 4 ml of fresh PS media in 12-well polystyrene culture plates. After 16 hours of static incubation at 28°C, the media was decanted gently and the wells were washed with autoclaved miliQ water to remove planktonic and loosely attached cells and adherence was monitored by staining the remaining firmly attached cells in biofilm with 1% crystal violet for 10 min at room temperature. Excess crystal violet stain was removed by washing the wells with autoclaved miliQ water. The attached cells bound crystal was solubilised with 1 ml of 90% ethanol and quantified by measuring the absorbance at 570 nm as described previously [63]. For visualization of biofilms, different Xcc strains were inoculated at PS, low-iron (PS + 100 μM DP) and iron supplemented (PS + 100 μM DP + 100 μM FeSO_4_) media at a concentration of 10^6^ cells/ml in 50-ml falcon (Tarsons) tubes containing a sterile glass slide half dipped in the medium. Tubes were incubated in shaking conditions at 28°C and 200 rpm for 24 h. Biofilms formed at the media–glass slide interface were washed with autoclaved miliQ water and stained with Syto9 and propidium iodide using the BacLight LIVE/DEAD Bacterial Viability Kit (L7012; Invitrogen, Eugene, OR, U.S.A.) as per the manufacturer’s instructions. Slides were mounted with 25% glycerol and analyzed by using a confocal laser-scanning microscope (LSM700; Carl Zeiss, Germany). 3D images were reconstructed using Zen 2012 software of Carl Zeiss. Biofilm thickness was quantified by measuring each biofilm (minimum 5 for each strain) at 10 randomly selected positions with taken series of horizontal (*xz*) optical sections at 0.39 μm intervals.

### Chromatin Immunoprecipitation (ChIP)-qPCR

ChIP assays were done as previously described [64] and Abcam X-ChIP protocol (http://www.abcam.com/protocols/cross-linking-chromatin-immunoprecipitation-x-chip-protocol) with modifications. Briefly, cultured bacterial cells were cross-linked with 0.75% formaldehyde for 10 minutes at room temperature. Cross-linking was stopped by the incubation with 0.125 M glycine for 5min. Fixed cells were washed with cold PBS twice and sonicated in the FA lysis buffer (50 mM HEPES-KOH pH7.5, 1 mM EDTA pH 8, 140 mM NaCl, 1% Sodium Deoxycholate, 1% Triton X-1000, 0.1% SDS and protease inhibitors (cOmplete Tablets, Mini, EDTA-free, EASYpack, Roche, Basel, Switzerland) using Bioruptor (Diagenode, Liège, Belgium). For immunoprecipitation, 50 μg of protein supernatant was diluted 10 times with RIPA buffer (50 mM Tris-HCl pH 8, 2 mM EDTA pH 8, 0.5% Sodium Deoxycholate, 150 mM NaCl, 1% NP-40, 0.1% SDS and Protease Inhibitors) and added 2 μg anti-HA antibody for overnight at 4°C. Immune complexes were precipitated with 20 μL of protein A/G beads (Santa Cruz) and washed sequentially 3 times with wash buffer (20 mM Tris-HCl pH 8, 2 mM EDTA pH 8, 150 mM NaCl, 0.1% SDS and 1% Triton X-100) and once with final wash buffer (20 mM Tris-HCl pH 8, 2 mM EDTA pH 8, 500 mM NaCl, 0.1% SDS and 1% Triton X-100). The chromatin was eluted in 150 μl of elution buffer (100 mM NaHCO_3_ and 1% SDS). All samples, including inputs, were de-cross linked using proteinase K at 65°C for 4 hrs and DNAs were extracted by Phenol: Chloroform and Ethanol precipitation followed by Real-time qPCR with the primers listed in S13 Table.

### Gel shift assay

EMSA was carried out as described previously [65] with few modifications. Briefly, 500 bp (approximately) DNA from the promoter of flagellar motor protein operon labelled with ATP γ-^32^P using Klenow enzyme. Reactions for gel shift assay contained 0.031pM of ^32^P -labelled DNA and 500 μM FeCl_3_ in the binding buffer (10 mM Tris–acetate, pH 8.0,8 mM MgCl_2_, 10 mM potassium acetate, 1 mM DTT, 110 μg poly dI-dC and 3.5% PEG 8000) in final volume of 20 μl. Protein concentrations used were 85 nM, 106 nM, 128 nM, 149 nM, 170 nM, 191 nM, 212 nM, 234 nM, 255 nM and 276 nM for binding to the *mot* promoter and 112 nM, 263 nM and 360 nM for binding to the *xss* promoter. For cold competition, unlabelled DNA fragments were added to the reactions. The reactions were incubated at 28°C for 30 min. Reaction were loaded onto a 4% non-denaturing polyacrylamide gel in TBE buffer at 200V for 3h in the cold room and were visualized by Fujifilm’s Multi Gauge software 3.0. Primers used for amplification of probes are listed in Supporting S13 Table.

### Circular dichroism (CD) spectroscopy

The protein samples with the concentrations of 0.2 mg/ml were dialyzed against a buffer (5 mM Tris-HCl, pH 7.0; 5 mM NaCl). 400 μl (1600 fmole) of the dialyzed protein sample was used in a 1mm cell length quartz cuvette for the spectral analysis in Jasco J-810 Spectropolarimeter (Mary's Court, Easton, MD, USA) using a data pitch of 1 nm and with a scanning speed, 50 nm/sec. We performed scanning at the wavelength range of 190–260 nm to study protein secondary structure. Data processing and spectral analysis was done using Spectra Manager version 1.53.01provided by Jasco (Mary's Court, Easton, MD, USA). Graph was plotted using Origin 8.1 provided by OriginLab (Northampton, MA, USA).

### Virulence assays on cabbage plants

***Xanthomonas campestris* pv.** *campestris* strains were grown to a density of 1.0 X 10^9^ cells/ml in PS medium with required antibiotics. Cells were pelleted down at 6,000 rpm for 10 min and resuspended in sterile miliQ water. Sterile scissors were dipped in bacterial cultures and 30-day old cabbage leaves (Indian Super Hybrid variety) were gently incised at the apex. Lesion length, CFU and migration of bacteria inside host leaves were recorded. Plant inoculated with sterile miliQ water was used as a control [66].

## Supporting Information

S1 Text Materials and Methods. Genetic screen by transposon mutagenesis and mapping of mutants; Generation of Δ*xibR*, Δ*glnG*, Δ*xssA*, Δ*xibR*Δ*glnG*, Δ*xibR*Δ*xssA*, Δ*fhuE*Δ*XC_0925*, Δ*fecR* and Δ*yciE*Δ*yciF*Δ*XC_3754* deletion mutants in wild-type Xcc 8004 background; Complementation analysis, generation of XibR point mutant and domain-swapped XibR and GlnG (NtrC) constructs; Generation of promoter fusions with GUS and GFP reporters; RNA Extraction, labeling, microarray hybridization, scanning and data analysis; *In planta* GUS expression assay for siderophore cluster and *xibR*; Protein expression and purification; Analysis of the upstream regulatory consensus sequence in *xibR* regulated genes; supporting references.
(DOC)

S1 Table Insertional mutants of *Xanthomonas campestris* pv. *campestris* with altered siderophore production. (DOC)

S2 Table Strains and plasmids used in this study. (DOC)

S3 Table *xibR* positively regulated genes but not influenced by iron starvation. (DOC)

S4 Table *xibR* negatively regulated genes but not influenced by iron starvation. (DOC)

S5 Table List of the genes positively regulated by iron starvation but not regulated by Xib*R*. (DOC)

S6 Table List of the genes negatively regulated by iron starvation but not influenced by *xibR*. (DOC)

S7 Table List of the genes negatively regulated by iron starvation and positively regulated by *xibR*. (DOC)

S8 Table List of the genes positively regulated by iron starvation and negatively regulated by *xibR*. (DOC)

S9 Table List of the genes positively regulated by both iron starvation and XibR. (DOC)

S10 Table List of the genes negatively regulated by both iron starvation and *xibR*. (DOC)

S11 Table Generations time of Xcc strains. (DOC)

S12 Table Analysis of the consensus sequence motif in the upstream regulatory sequences of XibR regulated genes. (XLS)

S13 Table The primers used in this study. (DOC)

S1 Video Time lapse live cell imaging of the wild-type Xcc 8004 in a chamber of sterile glass bottom cell culture dish containing PS medium with 0.3% agar. Each frame is of 300 millisecond interval at a speed of hundred frames per second for a total recording time of 4 min. Movie size = 11.3 MB; 12 sec.
(AVI)

S2 Video Time lapse live cell imaging of the wild-type Xcc 8004 in a chamber of sterile glass bottom cell culture dish containing low-iron PS medium with 0.3% agar (PS + 100 μM 2,2′-dipyridyl). Each frame is of 300 millisecond interval at a speed of hundred frames per second for a total recording time of 4 min. Movie size = 9.45 MB; 12 sec.
(AVI)

S3 Video Time lapse live cell imaging of the wild-type Xcc 8004 in a chamber of sterile glass bottom cell culture dish containing FeSO_4_ supplemented low-iron PS medium with 0.3% agar (PS + 100 μM 2,2′-dipyridyl + 100 μM FeSO_4_). Each frame is of 300 millisecond interval at a speed of hundred frames per second for a total recording time of 4 min. Movie size = 6.96 MB; 12 sec.
(AVI)

S1 Fig Siderophore overproduction phenotype of *xibR* mutants of Xcc. (A) Siderophore production by the wild-type *Xanthomonas campestris* pv. *campestris* 8004 after 48 h of growth on PSA-CAS plate supplemented without or with different concentration of iron specific chelator 2,2'-dipyridyl (DP).
(B) Location of the mTn*5* insertions and gene organization in the *Xanthomonas campestris* pv. *campestris* (Xcc 8004) genomic region containing the *xibR* gene. The arrows indicate transcriptional orientations of the genes. The *xibR* encodes a NtrC family of transcriptional regulator of 433 aa. The *xibRM2*, *xibRM1* and *xibRB1* mutants carry the mTn*5* insertions at 9^th^, 79^th^ and 425^th^ codon of *xibR*, indicated by inverted triangles.
(C) The transposon induced mutants *xibRM1*, *xibRM2*, and *xibRB1*, and a non-polar insertional mutant *xibRNPI* overproduce siderophore, indicated by the presence of an extended halo around the colony grown on peptone-sucrose agar plates containing chrome azurol sulphonate (CAS) + 75 μM 2,2’ dipyridyl (PSA-CAS + DP). Wild-type level of siderophore are restored by the addition of plasmid pSSP30 (wild-type *xibR* allele cloned in pHM1), indicated by + sign.
(D) Quantification of siderophore production. Average ratio of siderophore halo to colony diameter for different strains of Xcc grown on PSA-CAS-DP plate. Strains: Xcc 8004 (wild-type strain), Δ*xibR* (*xibR* deletion mutant), Δ*xibR*Δ*xssA* [*xibR* and *xssA* (*X* *anthomonas* siderophore synthesis A) double mutant], and strains harboring the plasmid containing either the wild-type *xibR* allele (pSSP30) or a point mutant of *xibR* in the putative conserved aspartate residue phosphorylation site (D55AXibR; pSSP39), *xssA* (pAP15; wild-type *xssA* allele) and pHM1 (vector). * indicate P < 0.05 in student’s t test (T-test) significant difference in the siderophore production between the wild-type Xcc 8004 harboring the plasmid containing the wild-type *xibR* allele (pSSP30) compared to the strain harboring the vector control (pHM1). Error bars represent SD of the mean (n = 3).
(E) Siderophore production phenotype of Δ*xibR*Δ*xssA* double deletion mutant [*xibR* and *xssA* (*X* *anthomonas* siderophore synthesis A)] or Δ*xibR*Δ*xssA* strain harboring the plasmid containing wild-type *xssA* allele (pAP15). Top: colony grown on PSA-CAS + DP plates. Bottom: wells on PSA-CAS plate containing siderophore isolated from cell free culture supernatant of different strains of Xcc grown under low-iron condition (PS + 100 μM) using Amberlite XAD-16 resin column chromatography. Cell normalized siderophore fractions were loaded in the wells made on PSA-CAS indicator plate.
(TIF)

S2 Fig Representative HPLC chromatogram. Representative HPLC chromatogram of siderophore isolated from the cell-free culture supernatants of wild-type Xcc 8004, Δ*xssA* (*Xanthomonas* siderophore synthesis A), Δ*xibR*, Δ*xibR* Δ*xssA* (*xibR* and *xssA* double deletion mutant), and strains harboring either the plasmid containing the wild-type *xibR* allele (pSSP30), the vector control (pHM1) or wild-type *xssA* allele (pAP15). Siderophore was isolated by Amberlite XAD-16 resin column chromatography and analyzed by HPLC (see supporting experimental procedures). Vibrioferrin peak was detected at 300 nm. Red color inset indicate the vibrioferrin peak corresponding to the standard purified vibrioferrin.
(TIF)

S3 Fig Transcriptional analysis of the *X* *anthomonas* siderophore synthesis (*xss*) cluster. Expression analysis was carried out with the β-glucuronidase (GUS) chromosomal reporter fusions (P*xssA*:: *gusA*) in the wild-type (Xcc 8004 P*xssA*::*gusA*) (A), Δ*xibR* (Δ*xibR* P*xssA*::*gusA*) (B), Δ*xibR* mutant harboring the complementing plasmid pSSP30 (Δ*xibR*/pSSP30 P*xssA*::*gusA*) (C). Strains were grown either in rich PS medium or PS medium supplemented with 100 μM DP (low-iron condition), 50 μM FeSO_4_ (iron-replete condition), and 100 μM DP + 100 μM FeSO_4_. β-Glucuronidase (GUS) activity was measured at 365/455 nm excitation/emission wavelength respectively and represented as cell normalized nanomoles of 4-methyl-umbelliferone (4-MU) produced per minute. Data are shown as mean ± S.D. (n = 3).
(D) Transcriptional analysis of plasmid borne P*xssA*::*gfp* expression in wild-type Xcc 8004 and Δ*xibR* strain. Relative GFP fluorescence of wild-type Xcc 8004 and Δ*xibR* strain harboring the GFP reporter plasmid pPROBE-GT (P*xssA*:: pPROBE-GT). Strains were grown either in rich PS medium or PS medium supplemented with 100 μM DP (low-iron condition), 50 μM FeSO_4_ (iron-replete condition), and 100 μM DP + 100 μM FeSO_4_. The error bars represent the standard deviations of the mean cell-normalized GFP fluorescence. Data are shown as mean ± S.E. (n = 3).
(E) Relative quantification of expression of the siderophore biosynthesis gene (*xssA*) of Xcc by real-time qRT-PCR. The wild-type Xcc 8004 and Δ*xibR* strains harboring either the plasmid containing the wild-type *xibR* allele (pSSP30) or the vector pHM1 (control), were grown to OD_600_ 1.2 in PS medium containing 100 μM 2′2,dipyridyl (DP). 16S ribosomal RNA was used as an endogenous control to normalize the RNA for cellular abundance. Data are shown as mean ± S.E. (n = 3).
(TIF)

S4 Fig Transcriptional analysis of *xibR* gene. (A) Relative quantification of expression of the *xibR* in the wild-type Xcc 8004 strain grown in PS (rich medium), PS + 100 μM FeSO_4_ (iron-replete), PS + 100 μM DP (low-iron), and PS + 100 μM DP + 100 μM FeSO_4_ by real-time qRT-PCR. ** P < 0.01 and * P < 0.05 in Student’s *t* test. Data shown in the graphs as mean ± S.E. (n = 3)
(B) Transcriptional analysis of *xibR* gene in Xcc. Expression analysis was carried out with the β-glucuronidase (GUS) chromosomal reporter fusion (P*xibR*:: *gusA*) in the wild-type (Xcc 8004 P*xibR*::*gusA*) strain grown either in PS medium or supplemented with 50 μM FeSO_4_. Error bars represent SD of the mean (n = 3) cell normalized Glucuronidase (GUS) activity represented as nanomoles of 4-methyl-umbelliferone (4-MU) produced per minute. * indicates P < 0.01 in Student’s *t* test, significant difference between the data obtained for the wild-type Xcc 8004 P*xibR*:: *gusA* strain grown in PS medium compared to those obtained from growth under iron-replete condition (PS + 50 μM FeSO_4_).
(TIF)

S5 Fig XibR and NtrC are two functionally distinct members of the NtrC family proteins. (A) Siderophore production on PSA-CAS-DP plates by different Xcc strains: Xcc strains: Xcc 8004 (wild-type), Δ*xibR* (*xibR* deletion mutant), Δ*glnG* (*glnG* deletion mutant), Δ*xibR*/pSSP30 (Δ*xibR* mutant harboring the plasmid containing the wild-type *xibR* allele; XibR), Δ*glnG*/pSSP34 (Δ*glnG* mutant harboring the plasmid containing wild-type *glnG* or *ntrC* allele; NtrC), Δ*xibR* (pSS61; XibR Swp ^Rec^), Δ*xibR* (pSS62; XibR Swp ^σ54^), Δ*xibR* (pSS63; XibR Swp^HTH^), Δ*glnG* (pSS64; NtrC Swp ^Rec^), Δ*glnG* (pSS65; NtrC Swp ^σ54^) and Δ*glnG* (pSS66; NtrC Swp^HTH^).
(B) Serial dilution spotting assay of different Xcc strains on modified MM9 minimal medium plates containing arginine as a sole nitrogen source.
(TIF)

S6 Fig Phylogenetic analysis of XibR sequence homologs. Phylogenetic dendrogram of XibR homolog’s in NCBI database was constructed by using the UPGMA method after amino acid sequence alignment with ClustalW and phylip 3.67 (mobyle.pasteur.fr/cgi-bin/portal.). *Xanthomonas campestris* pv. *campestris* str. 8004 (Xcc; AAY50800); *Xanthomonas fuscans* subsp. *fuscans* (Xff; CDF63051); *Xanthomonas axonopodis* pv. *citri* str. 306 (Xac; AAM38576); *Xanthomonas gardneri* (Xga; WP_046933196); *Xanthomonas arboricola* pv. *pruni* MAFF 301420 (Xap; GAE55687); *Xanthomonas oryzae* pv. *oryzae* KACC 10331 (Xoo; AAW73895); *Xanthomonas vesicatoria* (Xcv; WP_005988114); *Xanthomonas maliensis* (Xma; WP_022971710); *Pseudoxanthomonas dokdonensis* (Psedo; KRG68042); *Lysobacter* sp. URHA0019 (Lyso; WP_027082117); *Bordetella bronchiseptica* (Bor; WP_003811339). Scale 0.1 represents 10% differences between two sequences.
(TIF)

S7 Fig Heat map of differentially expressed genes. Heat map was generated using GeneSpringGX Software using the geomean fold (Log_2_) expression values of (A) Δ*xibR* mutant versus wild-type Xcc 8004 grown in PS medium (iron-replete condition)under rich medium; (B) Δ*xibR* mutant versus wild-type Xcc 8004 under low-iron condition (PS + DP); (C) wild-type Xcc 8004 grown under low-iron condition versus that grown under iron-replete condition; and (D) Δ*xibR* mutant grown under low-iron condition versus that grown under iron-replete condition. Color scale indicates log_2_ –fold change of expression (from green for downregulated to red for upregulated).
(TIF)

S8 Fig Schematic representation of the predicted low-iron condition and/or XibR regulated Xcc operon’s on the basis of micro array and sequence analysis. Predicted operon's which are either positively regulated (A) or repressed (B) by XibR but are not affected by low-iron condition. Operons which are either up-regulated (C) or down-regulated (D) under low-iron condition but are not affected by XibR. Operons which are either positively (E) or negatively (F) regulated by both XibR and low-iron. (G) Operons which are positively regulated by XibR and repressed by low-iron. (H) Operons which are repressed by XibR and induced under low-iron condition. Arrow indicates the direction of transcription of each predicted operon. The genes not differentially expressed in microarray were depicted as black boxes.
(TIF)

S9 Fig Role of XibR in iron uptake and storage. (A) Δ*xibR* mutant do not exhibit any defect in Fe^2+^ uptake. Incorporation of radiolabelled Fe^2+^ by Xcc 8004, Δ*xibR*, and strains harbouring the plasmid containing either the wild-type *xibR* allele (pSSP30) or a point mutant of *xibR* in the putative conserved aspartate residue phosphorylation site (D55AXibR; pSSP39). ^55^FeCl_3_ was reduced to ^55^Fe^2+^ in 1M sodium ascorbate. Uptake assay was performed in the presence of sodium ascorbate to maintain the FeCl_3_ in the reduced form. Data are shown as mean ± S.E. (n = 3).
(B and C) Relative quantification of expression of the ferrous iron transporter (*feoB*) and ferric uptake regulator (*fur*) of Xcc by real-time qRT-PCR. RNA was isolated from Xcc 8004, Δ*xibR* and strain harboring the plasmid containing the wild-type *xibR* allele (pSSP30) grown under PS, PS + 100 μM DP and PS + 100 μM DP + 100 μM FeSO_4_. 16S ribosomal RNA was used as an endogenous control to normalize the RNA for cellular abundance. Data are shown as mean ± S.E. (n = 3), ns = not significant.
(D) Absorbance at 600 nm of Xcc strains grown in PS broth with or without 0.5 μg/ml SNG and 0.01M sodium citrate after 16 and 42 h of growth are shown. Data are shown as mean ± S.E. (n = 3).
(E-G). The growth of Xcc 8004, Δ*xibR*, Δ*xibR*/pSSP30 and Δ*xibR*/pSSP39 strains in rich PS medium (E), low-iron medium (PS + intracellular ferrous iron chelator 150 μM 2′2′-bipyridyl) (F), and low-iron medium supplemented with iron (PS + 150 μM 2′2′-bipyridyl + 100 μM FeSO_4_) (G). Growth was monitored by determining the OD_600_. Data are shown as mean ± S.E. (n = 3).
(H and I) Relative quantification of expression of the putative ferritin-like protein (XC_3752) and periplasmic iron dicitrate sensor (XC_0557) of Xcc by real-time qRT-PCR. RNA was isolated from Xcc 8004, Δ*xibR* and strain harboring the plasmid containing the wild-type *xibR* allele (pSSP30) grown in rich PS media, PS + 100 μM DP and PS + 100 μM DP + 100 μM FeSO_4_. 16S ribosomal RNA was used as an endogenous control to normalize the RNA for cellular abundance. Data are shown as mean ± S.E. (n = 3). ** Indicating p-value < 0.01 statistical significance by paired student t-test. ns = not significant.
(J-L) The growth of Xcc 8004, Δ*fhuE* Δ*XC_0925*, Δ*fecR* and Δ*yciE* Δ*yciF* Δ*XC_3754* in rich PS medium (J), low-iron medium (PS + intracellular ferrous iron chelator 150 μM 2′2′-dipyridyl) (K), and low-iron medium supplemented with iron (PS + 150 μM 2′2′-dipyridyl + 100 μM FeSO_4_) (L). Growth was monitored by determining the OD_600_. Data is mean of three biological replicates. Error bars are showing SEM.
(M) SNG sensitivity plate assay. Xcc 8004, Δ*fhuE* Δ*XC_0925*, Δ*fecR* and Δ*yciE* Δ*yciF* Δ*XC_3754* were grown in PS media at a density of 1 × 10^9^ cells/ml. 4μL of cultures from each serial dilution was spotted on PSA plates containing 1μg/ml SNG and 0.01M sodium citrate. Plates were incubated for 72 h at 28°C to observe bacterial growth.
(N) Absorbance at 600 nm of Xcc 8004, Δ*fhuE* Δ*XC_0925*, Δ*fecR* and Δ*yciE* Δ*yciF* Δ*XC_3754* grown in PS broth with or without 0.5 μg/ml SNG and 0.01M sodium citrate after 16 and 42 h of growth are shown. Data are shown as mean ± S.E. (n = 3).
(TIF)

S10 Fig Role of XibR and low-iron condition on the expression of flagellar genes involved in regulation and assembly of different flagellar component. (A) Schematic representation of the model of the flagellar transcriptional cascade in Xcc. Expression and assembly of flagellar components takes place in a temporal fashion, in which the Class I protein σ54 and FleQ regulates the expression of class II genes, which are required for site selection and basal body formation. Class III genes encode proteins required for flagellar filament, cap proteins and motility regulatory proteins. Locus tags of flagellar genes encoding proteins are shown in bracket. Based on expression analysis by microarray, genes (locus tags) which are positively regulated by XibR are shown in red color. Genes (locus tags) which are positively regulated by both XibR and low-iron condition are shown as underline. Genes which are not differentially expressed in microarray were depicted in black color.
(B) Quantitative chemotaxis capillary assay with different Xcc strains grown under PS, PS + 100 μM DP and PS + 100 μM DP + 100 μM FeSO_4_. Cells were incubated at 28°C with capillaries containing potassium glutamate (4.9 mM) and PBS. Relative chemotaxis response was determined by migrated bacterial cells in capillary containing potassium glutamate over the capillary containing PBS. Data are shown as mean ± S.E. (n = 3). The experiment was repeated two times.
(C) Relative quantification of the expression of *flgD* by real-time qRT-PCR. Different strains of Xcc; Xcc 8004, Δ*xibR* and strain harbouring the plasmid containing the wild-type *xibR* allele (pSSP30), were grown to OD_600_ 1.2 in PS, PS + 100 μM DP and PS + 100 μM DP + 100 μM FeSO_4_. 16S ribosomal RNA was used as an endogenous control to normalize the RNA for cellular abundance. Data are shown as mean ± S.E. (n = 3).
(D) Expression analysis of *flgG* operon in wild-type (Xcc 8004 P_*flgG*::*gusA*_) and Δ*xibR* (Δ*xibR*P_*flgG*::*gusA*_) grown under PS, PS + 100 μM DP and PS + 100 μM DP + 100 μM FeSO_4_ while monitoring the β-glucuronidase (GUS) activity. Data are shown as mean ± S.D. (n = 3).
* Indicating p-value < 0.05, ** Indicating p-value < 0.01 and *** indicating p-value < 0.001 statistically significance by paired student t-test.
(TIF)

S11 Fig Cell aggregation phenotype of different strains of Xcc. (A) Saturated cultures were grown in rich PS medium and the tubes were kept at room temperature for 4 hours for the observation of aggregation phenotype. Wild-type Xcc 8004 and Δ*xibR*/pSSP30 exhibit disperse phenotype than Δ*xibR* and Δ*xibR*/pSSP39.
(B) Average biofilm thickness of different strains of Xcc formed on the glass slide at the air-media interphase. Different Xcc strains were inoculated in PS, low-iron (PS + 100 μM DP) and iron supplemented (PS + 100 μM DP + 100 μM FeSO_4_) media at a concentration of 10^6^ cells/ml and grown for 24 h. For quantification of the thickness, five independent biofilms were scanned with CLSM at ten randomly selected positions and thickness was determined through height of the biofilm. Data are shown as mean ± S.E. (n = 3).
(TIF)

S12 Fig Siderophore production by Xcc strains on PSA-CAS plate with 75 μM DP. (A) wild-type Xcc 8004, Δ*xibR*, Δ*xibR*/pSSP30 and Δ*xibR*/pSSP80
(B) Strains wild-type Xcc 8004, Δ*xibR*, Δ*xibR*/pSSP30 and Δ*xibR*/pSSP81
(C) SDS-PAGE for purified XibR with C-terminal His-tag; lane 1 is Unstained Protein MW Marker (ThermoFisher Scientific, Waltham, MA, USA), lane 2, 3, and 4 are different fractions of purified XibR.
(D) Western blot for XibR with C-terminal His-tag using anti-His antibody. Lane 1 = Prestained Protein MW Marker (ThermoFisher Scientific, Waltham, MA, USA); lane 2 = un-induced XibR in bl21 (DE3); and lane 3 = induced XibR in bl21 (DE3).
(TIF)

S13 Fig **Electrophoretic mobility shift assay (EMSA) showing binding of XibR to a 32P-labeled motA probe with increasing concentration of either ferric (B) or ferrous (D) form of iron.** (A) EMSA showing binding of XibR to a ^32^P-labeled *xss* (-188 to +205) probe. More DNA-protein binary complex was observed with the increase in the concentration of XibR protein. (C) EMSA showing binding of XibR to a ^32^P-labeled *motA* probe in the presence of other divalent metal ions and ferric iron. Presence of deferoxamine mesylate with FeSO_4_ decreased binding of XibR to the *motA* probe (lane 3).
(TIF)

S14 Fig *In silico* analysis of consensus sequence motifs in promoter of *flg*, *mot* and *xss* operons. (A) Sequence logos for the five consensus motifs identified by MEME. (B) Schematic representation of relative position of conserved motifs (shown in red, blue, green, magenta and orange color boxes) on the *flg*, *mot* and *xss* promoter sequences.
(TIF)

S15 Fig Exogenous iron supplementation rescued the siderophore overproduction phenotype of the Δ*xibR* mutant. Different strains of Xcc were grown on PSA-CAS medium containing without or with 50 μM 2,2′-dipyridyl (DP). For iron supplementation, either FeCl_3_ or FeSO_4_ were added in PSA-CAS + DP medium at a concentration of 10 and 20 μM.
(TIF)
