# Supplementary material for: Co-regulation of Iron Metabolism and Virulence Associated Functions by Iron and XibR, a Novel Iron Binding Transcription Factor, in the Plant Pathogen Xanthomonas
Source: PLoS Pathog. 2016 Nov 30;12(11):e1006019. doi: 10.1371/journal.ppat.1006019 (PMC5130282; doi:10.1371/journal.ppat.1006019)
Supplement: S1 Text — Genetic screen by transposon mutagenesis and mapping of mutants; Generation of ΔxibR, ΔglnG, ΔxssA, ΔxibRΔglnG, ΔxibRΔxssA, ΔfhuEΔXC_0925, ΔfecR and ΔyciEΔyciFΔXC_3754 deletion mutants in wild-type Xcc 8004 background; Complementation analysis, generation of XibR point mutant and domain-swapped XibR and GlnG (NtrC) constructs; Generation of promoter fusions with GUS and GFP reporters; RNA Extraction, labeling, microarray hybridization, scanning and data analysis; In planta GUS expression assay for siderophore cluster and xibR; Protein expression and purification; Analysis of the upstream regulatory consensus sequence in xibR regulated genes; supporting references. (DOC) [file ppat.1006019.s001.doc]

**Supporting text**

**Supporting Materials and Methods**

**Genetic screen by transposon mutagenesis and mapping of mutants**

We used pRL27::Tn*5* construct containing kanamycin encoding gene *aph*, hyperactive Tn*5* transposase (*tnp*) under the control of the *tetA* promoter (*tetA*p) and λ*pir* dependent origin of replication *ori*R6K, for transposon-mediated random mutagenesis which is 1000 fold more efficient than wild type Tn5 [1]. Transformation of pRL27::Tn5 construct in Xcc 8004 was done by electroporation and conjugation. Transposon inserted mutant strains were assayed for siderophore production on CAS agar plate. A Tn*5* inserted library of around 12000 colonies was screened on CAS agar plates and identified 35 strains which were overproducing siderophores. Single pRL27::tn5 insertion in each mutant strain was verified by marker exchange mutagenesis. Locations of transposon insertion in the siderophores overproducing mutants were mapped by plasmid rescue [1] and TAIL-PCR (Thermal asymmetric interlaced PCR; [2]). For the plasmid rescue, genomic DNA was isolated, digested with BamH1, one of the restriction enzymes which cut outside the transposon, ligated and transformed in DH5α/ λ*pir* [1]. Further plasmids were isolated and sequenced at National Genomics and Transcriptomics Facility, Hyderabad, India. TAIL-PCR mediated mapping of transposon insertion was done by amplifying genomic sequences flanking the insertion sites [2]. The used set of transposon-specific nested primers (SC Intsx1, SC Clamp, SC tnpRL17-1, SC tnpRL13-2, SC Extdx, SC Extsx, SC Ext-1) and arbitrary degenerate primers (SC ARB-1A, SC ARB-1B, SC ARB1, SC ARB2, SC ARB8) are listed in Supporting Table S13.

**Generation of Δ*xibR*, Δ*glnG*, Δ*xssA*, Δ*xibR*Δ*glnG*, Δ*xibR*Δ*xssA*, Δ*fhuE*Δ*XC_0925*, Δ*fecR* and Δ*yciE*Δ*yciF*Δ*XC_3754* deletion mutants in wild-type Xcc 8004 background.**

Chromosomal deletion of Δ*xibR*, Δ*glnG*, Δ*xssA*, Δ*xibR*Δ*glnG*, Δ*xibR*Δ*xssA*, Δ*fhuE*Δ*XC_0925*, Δ*fecR* and Δ*yciE*Δ*yciF*Δ*XC_3754* genes in Xcc 8004 background was performed by allelic exchange and homologous recombination using suicide vector pK18mobsacB [3], harboring the 5’ and 3’ flanking regions of the target gene (Table S2). The 5’ flanking regions of *xibR*, Δ*glnG*, Δ*xssA* and Δ*fecR* were amplified with primers (listed in Table S13): SCP3F & SCP4R, SCP20F & SCP21R, Sidxcdel1F & Sidxcdel1R and SCP64F & SCP65R respectively while 3’ flanking regions were amplified with primers SCP5F & SCP6R, SCP22F & SCP23R, Sidxcdel2F & Sidxcdel2R and SCP66F & SCP67R respectively. The 5’ flanking regions for simultaneous double deletion Δ*fhuE*Δ*XC_0925* and simultaneous triple deletion Δ*yciE*Δ*yciF*Δ*XC_3754* were amplified with primers SCP59F &SCP60R and SCP69F & SCP70R respectively and 3’ flanking regions were amplified with primers SCP61F &SCP62R and SCP71F & SCP72R respectively. Amplified PCR products of flanking regions and the pK18mobsacB vectors were digested with appropriate restriction enzymes, ligated, transformed into *E. coli* DH5α cells and selected on the nalidixic acid and kanamycin containing LB agar plates. These pK18mobsacB vectors with respective deletion constructs (Table S2) were transformed into electrocompetent wild-type Xcc 8004 cells. Transformants with single crossover were selected on Nutrient agar plate containing rifampicin and kanamycin. The selected colonies were passaged under antibiotic free condition in NB (nutrient broth) medium. While passaging, the second recombination occurs and plasmid background gets removed and then selected the appropriate colonies on PSA plate containing 5% sucrose. Deletion was confirmed using outward primers (listed in Table S13). Double deletion Δ*xibR*Δ*glnG* and Δ*xibR*Δ*xssA* mutants were generated by making the 2^nd^ deletion in 1^st^ deletion background by the above mention procedure. The Δ*xibR*, Δ*glnG*, Δ*xssA*, Δ*fhuE*Δ*XC_0925*, Δ*fecR* and Δ*yciE*Δ*yciF*Δ*XC_3754* deletion mutants were identified by PCR using outward primers SCP28F & SC29R, SCP32F & SCP33R, SCP52F & SCP52R, SCP74F & SCP75R, SCP76F & SCP77R and SCP69F & SCP73R respectively. Nucleotide sequences of all the mentioned primers used in the generation and confirmation of deletion strains are given in supplementary Table S13.

**Complementation analysis, generation of XibR point mutant and domain-swapped XibR and GlnG (NtrC) constructs**

Full-length *xibr* with the promoter, *xibR*, *glnG*, C-terminal his tagged *xibR* and C-terminal HA-tagged *xibR* were amplified from genomic DNA of wild-type Xcc 8004 using the primers SC60F & SC62R, SC63F & SC63R, SC71F & SC73R, SC63F & SCP18R and SC63F & SCP19R respectively. PCR products and expression vector pHM1 were digested with respective restriction enzymes, ligated and transformed in DH5α cells. Positive clones were selected on spectinomycin and nalidixic acids containing LB agar plates and further confirmed by colony PCR, plasmid isolation and sequencing. Positive clones were mobilized into different Xcc mutant strains. Point mutant D55AXibR encoding gene was made by amplifying the plasmid pSSP31 (pK18mob vector with full-length *xibR*) with the primers with point mutation D55AXibR F & D55AXibR R and transforming in DH5α after DpnI treatment. A point mutation was confirmed by sequencing of positive clones then amplified with SC63F & SC62R. Amplified point mutant D55AXibR encoding PCR product and expression vector pHM1 were digested with HindIII and EcoR1, ligated, transformed in DH5α and selected on spectinomycin and nalidixic acids containing LB agar plates. Subsequently, the clone was confirmed by colony PCR, plasmid isolation and sequencing. A positive clone was mobilized into Δ*xibR* deletion strain. The expression vector pHM1 with swapped domain proteins encoding genes: pSSP61, pSSP62, pSSP63, pSSP64, pSSP65 and pSSP66 (see Table S2) were made by cloning of swapped amplification products to pHM1 which were amplified by overlapping extension PCR using the primers N2Srec F & N2Srec R, N2Ssigma F & N2Ssigma R, N2Sdnab F & N2Sdnab R, S2Nrec F & S2Nrec R, S2Nsigma F & S2Nsigma R and S2Ndnab F & S2Ndnab R respectively. Nucleotide sequences of all the primer used in the generation of complementary strains are given in Supporting Table S13.

**Generation of promoter fusions with GUS and GFP reporters**

Chromosomal glucuronidase (GUS) reporter gene fusions were made with plasmid pVO155 containing promoterless *gusA* gene [4]. Putative promoters of *flg*, *mot* and *xss* cluster and *xibR* were amplified using the primers SCP_flg1_prom F & SCP_flg1_prom R1, SCP_mot_prom F & SCP_mot_prom R1, SCP_sid_prom F & SCP_sid_prom R1 and SCP_xibR_prom F & SCP_xibR_prom R1, respectively (Table S13). Amplified promoter fragments were then digested with HindIII and XbaI and cloned upstream to the promoterless *gusA* gene in pVO155. GFP reporter gene fusions were made in GFP reporter plasmid pPROBEGT harboring promoterless GFP [5]. Putative promoters of *flg* cluster, *mot* cluster, *xss* cluster and *xibR* were amplified using the primers SCP_flg1_prom F & SCP_flg1_prom R2, SCP_mot_prom F & SCP_mot_prom R2, SCP_sid_prom F & SCP_sid_prom R2, SCP_xibR_prom F & SCP_xibR_prom R2, respectively. Amplified fragments were digested with HindIII and EcoR1 and cloned upstream to the promoter less *gfp* gene into the similarly digested pPROBEGT. The promoter fusion constructs were mobilized from *E.coli* DH5α to *E.coli* S17-1 by triparental mating. Subsequently, the resulting promoter fusion constructs were introduced into different strains of Xcc by biparental mating using *E. coli*. Nucleotide sequences primers used in the generation of promoter fusions with GUS and GFP reporters are given in Supporting Table S13.

**RNA Extraction, labeling, microarray hybridization, scanning and data analysis**

Whole cell RNA was isolated from mid-exponential phase cultures of Xcc 8004 wild-type and Δ*xibR* mutant grown in the rich PS and iron-deprived media (PS + 100 µM 2, 2’-bipyridyl) by Trizol (Invitrogen, CA, USA) as per manufacturer’s instruction. RNA concentration and purity was determined at an optical density ratio of 260/280 using the Nanodrop® ND-1000 spectrophotometer (NanoDrop Technologies, Wilmington, DE) and the integrity of total RNA was verified on an Agilent 2100 Bioanalyzer using the RNA 6000 Nano LabChip (Agilent Technologies, Santa Clara, California, USA).

The samples for Gene expression were labelled using Agilent Quick-Amp labelling Kit (p/n5190-0442). 2000 ng each of total RNA were polyadenylated and purified by precipitation. 500 ng of polyadenylated total RNA were reverse transcribed at 40°C using a T7 primer with the T7 polymerase promoter converted to double stranded cDNA. Synthesized double stranded cDNA were used as a template for cRNA generation. cRNA was generated by in vitro transcription and the dye Cy3 CTP (Agilent) was incorporated during this step. The cDNA synthesis and in vitro transcription steps were carried out at 40°C. Labelled cRNA was cleaned up using Qiagen RNesay columns (Qiagen, Cat No: 74106) and quality assessed for yields and specific activity using the Nanodrop ND-1000. 600 ng of labelled cRNA sample were fragmented at 60 º C and hybridized onto a Genotypic designed Custom *Xanthomonas campestris* GXP, 8X15K (AMADID No: 028737) arrays. Fragmentation of labelled cRNA and hybridization were done using the Gene Expression Hybridization kit of (Agilent Technologies, In situ Hybridization kit, Part Number 5190-0404). Hybridization was carried out in Agilent’s Surehyb Chambers at 65º C for 16 hours. The hybridized slides were washed using Agilent Gene Expression wash buffers (Agilent Technologies, Part Number 5188-5327) and scanned using the Agilent Microarray Scanner (Agilent Technologies, Part Number G2505C) at 5-micron resolution.

Data extraction from Images was done using Agilent Feature Extraction software. Feature extracted data was analyzed using GeneSpring GX version 11software from Agilent. Normalization of the data was done in GeneSpring GX using the 75^th^ percentile shift and Normalization to Specific Samples. Samples were grouped based on the replicates. Significant genes up and down regulated showing 0.6 fold geomean ratio (1.5 actual fold change) differences among the samples were identified. T-test p-value was calculated using volcano Plot. Differentially regulated genes were clustered using hierarchical clustering to identify significant gene expression patterns.

***In planta* GUS expression assay for siderophore cluster and *xibR***

The Xcc 8004 and Δ*xibR* strains with or without chromosomal *gusA* transcriptional fusions were injected in veins of cabbage plants in the presence and absence of 250 µM FeSO_4_. Leaves were crushed in sterile miliQ water and dilution plated to determine the CFU at every alternate day between 0-14 days after infection. At the same time for GUS assay, dissolved the crushed leaves in 1 ml of extraction buffer (50 mM sodium dihydrogen phosphate [pH 7.0], 0.1% Triton X-100, 10 mM EDTA, 0.1% sodium lauryl sarcosine, and 10 mM β-mercaptoethanol [6] without MUG substrate (4-methylumbelliferyl β-D-glucuronide). Further, 250 μl of extraction buffer containing MUG substrate was added to the plant extract and incubated at 37°C for an appropriate time. Subsequently, reactions were terminated after addition of 675 μl of 0.2 M Na2CO3 into 75μl of reaction mixture and fluorescence was measured with 4-methyl-umbelliferone (MU; Sigma) as the standard at excitation 365nm and emission 455nm of wavelength. β-Glucuronidase activity for GUS assays was expressed as nanomoles of MU produced/minute/10^9^ cells.

**Protein expression and purification**

For expression of XibR protein in *E*. *coli*, pET 23a expression vector harboring the C-terminal His-tagged XibR encoding gene (pET (*lac*-P/*xibR*) was transformed into *E. coli* (*Plys S*) strain. One milliliter of the grown culture was inoculated into 250 ml LB broth with 100 µM ampicillin (final concentration). After the cells were grown at 37°C to an OD (0.6-0.7), His-fusion protein was induced by addition of IPTG to a final concentration of 0.5 mM and cell growth was continued overnight at 28°C and 200 rpm. The cells were harvested and the soluble His-tagged proteins were purified by affinity chromatography with Talon resin (CloneTech). Briefly, each cell pellet was resuspended in 10 ml of the column buffer (50 mM Tris-HCl, pH 7.0; 150 mM NaCl; 1% Sarcosine) with 1 mM PMSF, incubated on ice for 10 min, sonicated, centrifuged and the supernatant was added to 2 ml of Talon resin slurry. After washing with 700 ml of column buffer with 8 mM Imidazole, the proteins were eluted using 10 ml of the column buffer with 200 mM Imidazole. The protein samples were dialyzed against column buffer and used for further downstream experiments. The samples were aliquot for storage at -80 °C. Protein concentrations were measured using the Bio-Rad Protein Assay reagent with BSA as a standard. About 10 µg of each protein sample was analyzed by 12% SDS-PAGE to verify molecular weight and purity.

***In silico* analysis of consensus motifs in XibR regulated promoters**

MEME program version 4.11.2 [7] was used to identify motifs in *flg*, *mot* and *xss* promoter sequences, which were identified by EMSA and ChIP assays. We use the MEME command
“meme <fasta file>  -mod anr -dna -minsites 3 -nmotifs 5 -minw 5  -maxw 15 -revcomp ”
to detect the top 5 motifs. These 5 identified motifs were scanned against the genes listed in supplementary tables S3 (401 genes), S4 (163 genes), S7 (73 genes), S8 (12 genes), S9 (73 genes) and S10 (11 genes) datasets.  The 200 bp region upstream of each gene was identified and analyzed by FIMO program version 4.11.2 for scanning the occurrences of motifs in DNA sequence using default parameters. The results are shown in Supplementary Table S12. No motif occurrence was identified in differentially expressed gene in Table S10.

**Supporting Figure Legends**

**Fig. S1. Siderophore overproduction phenotype of *xibR* mutants of Xcc.**

(A) Siderophore production by the wild type *Xanthomonas campestris* pv. *campestris* 8004 after 48 h of growth on PSA-CAS plate supplemented without or with different concentration of iron specific chelator [2,2'-dipyridyl](https://www.google.co.in/url?sa=t&rct=j&q=&esrc=s&source=web&cd=1&cad=rja&uact=8&ved=0ahUKEwi88Za_irrJAhWTBY4KHYvaCXkQFggbMAA&url=http%3A%2F%2Fwww.scbt.com%2Fdatasheet-206502-2-2-dipyridyl.html&usg=AFQjCNHhYZUZSwsgOSHyXwYq_O-DgIvBfg&sig2=jG0lu4oHDJlZz4CckkE7Pg&bvm=bv.108194040,d.c2E)(DP).

(B) Location of the mTn*5* insertions and gene organization in the *Xanthomonas campestris* pv. *campestris* (Xcc 8004) genomic region containing the *xibR* gene. The arrows indicate transcriptional orientations of the genes. The *xibR* encodes a NtrC family of transcriptional regulator of 433 aa. The *xibRM2*, *xibRM1* and *xibRB1* mutants carry the mTn*5* insertions at 9^th^, 79^th^ and 425^th^ codon of *xibR*, indicated by inverted triangles.

(C) The transposon induced mutants *xibRM1*,*xibRM2*, and *xibRB1*, and a non-polar insertional mutant *xibrNPI* overproduce siderophore, indicated by the presence of an extended halo around the colony grown on peptone-sucrose agar plates containing chrome azurol sulphonate (CAS) + 75 µM 2,2’ dipyridyl (PSA-CAS + DP). Wild type level of siderophore are restored by the addition of plasmid pSSP30 (wild type *xibR* allele cloned in pHM1), indicated by + sign.

(D) Quantification of siderophore production. Average ratio of siderophore halo to colony diameter for different strains of *Xcc*grown on PSA-CAS-DP plate. Strains: Xcc 8004 (wild type strain), Δ*xibR* (*xibR* deletion mutant), Δ*xibR*Δ*xssA* [*xibR* and *xssA* (xanthomonas siderophore synthesis A) double mutant], and strains harboring the plasmid containing either the wild type *xibR* allele (pSSP30) or a point mutant of *xibR* in the putative conserved aspartate residue phosphorylation site (D55AXibR; pSSP39), *xssA* (pAP15; wild type *xssA* allele) and pHM1 (vector). * indicate P < 0.05 in student’s t test (T-test) significant difference in the siderophore production between the wild type Xcc8004 harboring the plasmid containing the wild type *xibR* allele (pSSP30) compared to the strain harboring the vector control (pHM1). Error bars represent SD of the mean (n=3).

(E) Siderophore production phenotype of Δ*xibR*Δ*xssA* double deletion mutant [*xibR* and *xssA* (xanthomonas siderophore synthesis A)] or Δ*xibR*Δ*xssA* strain harboring the plasmid containing wild type *xssA* allele (pAP15). Top: colony grown on PSA-CAS + DP plates. Bottom: wells on PSA-CAS plate containing siderophore isolated from cell free culture supernatant of different strains of *Xcc* grown under low-iron condition (PS + 75 µM) using Amberlite XAD-16 resin column chromatography. Cell normalized siderophore fractions were loaded in the wells made on PSA-CAS indicator plate.

**Fig. S2**. **Representative HPLC chromatogram.**

Representative HPLC chromatogram of siderophore isolated from the cell-free culture supernatants of wild type Xcc8004, Δ*xssA* (xanthomonas siderophore synthesis A), Δ*xibR*, Δ*xibR* Δ*xssA* (*xibR* and *xssA* double deletion mutant), and strains harboring either the plasmid containing the wild type *xibR* allele (pSSP30), the vector control (pHM1) or wild type *xssA* allele (pAP15). Siderophore was isolated by Amberlite XAD-16 resin column chromatography and analyzed by HPLC (see supporting experimental procedures). Vibrioferrin peak was detected at 300 nm. Red color inset indicate the vibrioferrin peak corresponding to the standard purified vibrioferrin [8].

**Fig. S3**. **Transcriptional analysis of the *Xanthomonas* siderophore synthesis (*xss*) cluster.** Expression analysis was carried out with the β-glucuronidase (GUS) chromosomal reporter fusions (P*xssA:: gusA*) in the wild-type (Xcc 8004 P*xssA*::*gusA*) (A), Δ*xibR* (Δ*xibR* P*xssA*::*gusA*) (B), Δ*xibR* mutant harboring the complementing plasmid pSSP30 (Δ*xibR*/pSSP30 P*xssA*::*gusA*) (C). Strains were grown either in rich PS medium or PS medium supplemented with 100 µM DP (low-iron condition), 50 µM FeSO_4_ (iron-replete condition), and 100 µM DP + 100 µM FeSO_4_. β-Glucuronidase (GUS) activity was measured at 365/ 455 nm excitation/emission wavelength respectively and represented as cell normalized nanomoles of 4-methyl-umbelliferone (4-MU) produced per minute. Data are shown as mean ± S.D. (n=3).

(D) Transcriptional analysis of plasmid borne P*xssA*::*gfp* expression in wild type Xcc8004 and Δ*xibR* strain. Relative GFP fluorescence of wild type Xcc8004 and Δ*xibR* strain harboring the GFP reporter plasmid pPROBE-GT (P*xssA*:: pPROBE-GT). Strains were grown either in rich PS medium or PS medium supplemented with 100 µM DP (low-iron condition), 50 µM FeSO_4_ (iron-replete condition), and 100 µM DP + 100 µM FeSO_4_. The error bars represent the standard deviations of the mean cell-normalized GFP fluorescence. Data are shown as mean ± S.E. (n=3).

(E) Relative quantification of expression of the siderophore biosynthesis gene (*xssA*) of *Xcc* by real-time qRT-PCR. The wild-type Xcc 8004 and Δ*xibR* strains harboring either the plasmid containing the wild type *xibR* allele (pSSP30) or the vector pHM1 (control), were grown to OD_600_ 1.2 in PS medium containing 100 µM 2′2,dipyridyl (DP). 16S ribosomal RNA was used as an endogenous control to normalize the RNA for cellular abundance. Data are shown as mean ± S.E. (n=3).

**Fig. S4. Transcriptional analysis of *xibR* gene.**

(A) Relative quantification of expression of the *xibR* in the wild type Xcc 8004 strain grown in PS (rich medium), PS + 100 µM FeSO_4_ (iron-replete), PS + 100 µM DP (low-iron), and PS + 100 µM DP + 100 µM FeSO_4_ by real-time qRT-PCR. ** P < 0.01 and * P < 0.05 in Student’s *t* test. Data shown in the graphs as mean ± S.E. (n=3)

(B) Transcriptional analysis of *xibR* gene in Xcc. Expression analysis was carried out with the β-glucuronidase (GUS) chromosomal reporter fusion (P*xibR:: gusA*) in the wild-type (Xcc 8004 P*xibR*::*gusA*) strain grown either in PS medium or supplemented with 50 µM FeSO_4_. Error bars represent SD of the mean (n=3) cell normalized Glucuronidase (GUS) activity represented as nanomoles of 4-methyl-umbelliferone (4-MU) produced per minute. * indicates P < 0.01 in Student’s *t* test, significant difference between the data obtained for the wild-type Xcc 8004 P*xibR* ::*gusA* strain grown in PS medium compared to those obtained from growth under iron-replete condition (PS + 50 µM FeSO_4_).

**Fig. S5. XibR and NtrC are two functionally distinct members of the NtrC family proteins.**

(A) Siderophore production on PSA-CAS-DP plates by different Xcc strains: Xcc strains: Xcc 8004 (wild type), Δ*xibR* (*xibR* deletion mutant), Δ*glnG* (*glnG* deletion mutant), Δ*xibr*/pSSP30 (Δ*xibR* mutant harboring the plasmid containing the wild type *xibR* allele; XibR), Δ*glnG*/pSSP34 (Δ*glnG* mutant harboring the plasmid containing wild type *glnG* or *ntrC* allele; NtrC), Δ*xibR* (pSS61; XibR Swp ^Rec^), Δ*xibR* (pSS62; XibR Swp ^σ54^), Δ*xibR* (pSS63; XibR Swp^HTH^), Δ*glnG* (pSS64; NtrC Swp ^Rec^), Δ*glnG* (pSS65; NtrC Swp ^σ54^) and Δ*glnG* (pSS66; NtrC Swp^HTH^).

(B) Serial dilution spotting assay of different Xcc strains on modified MM9 minimal medium plates containing arginine as a sole nitrogen source.

**Fig. S6**. Phylogenetic analysis of XibR sequence homologs. Phylogenetic dendrogram of XibR homologs in NCBI database was constructed by using the UPGMA method after amino acid sequence alignment with ClustalW and phylip 3.67 (mobyle.pasteur.fr/cgi-bin/portal.). *Xanthomonas campestris* pv. *campestris* str. 8004 (Xcc; AAY50800); *Xanthomonas fuscans* subsp. *fuscans* (Xff; CDF63051); *Xanthomonas axonopodis* pv. *citri* str. 306 (Xac; AAM38576); *Xanthomonas gardneri* (Xga; WP_046933196); *Xanthomonas arboricola* pv. *pruni* MAFF 301420 (Xap; GAE55687); *Xanthomonas oryzae* pv. *oryzae* KACC 10331 (Xoo; AAW73895); *Xanthomonas vesicatoria* (Xcv; WP_005988114); *Xanthomonas maliensis* (Xma; WP_022971710); *Pseudoxanthomonas dokdonensis* (Psedo; KRG68042); *Lysobacter* sp. URHA0019 (Lyso; WP_027082117); *Bordetella bronchiseptica* (Bor; WP_003811339). Scale 0.1 represents 10% differences between two sequences.

**Fig. S7. Heat map of differentially expressed genes.**

Heat map was generated using GeneSpringGX Software using the geomean fold (Log_2_) expression values of (A) Δ*xibR* mutant versus wild-type Xcc 8004 grown in PS medium (iron-replete condition)under rich medium; (B) Δ*xibR* mutant versus wild-type Xcc 8004 under low-iron condition (PS + DP); (C) wild-type Xcc 8004 grown under low-iron condition versus that grown under iron-replete condition; and (D) Δ*xibR* mutant grown under low-iron condition versus that grown under iron-replete condition. Color scale indicates log_2_–fold change of expression (from green for downregulated to red for upregulated).

**Fig. S8. Schematic representation of the predicted low-iron condition and/or XibR regulated Xcc operon’s on the basis of micro array and sequence analysis.**

Predicted operon's which are either positively regulated (A) or repressed (B) by XibR but are not affected by low-iron condition. Operons which are either up-regulated (C) or down-regulated (D) under low-iron condition but are not affected by XibR. Operons which are either positively (E) or negatively (F) regulated by both XibR and low iron. (G) Operons which are positively regulated by XibR and repressed by low-iron. (H) Operons which are repressed by XibR and induced under low iron condition. Arrow indicates the direction of transcription of each predicted operon. The genes not differentially expressed in microarray were depicted as black boxes.

**Fig. S9. Role of XibR in iron uptake and storage.**

(A) Δ*xibR* mutant do not exhibit any defect in Fe^2+^ uptake. Incorporation of radiolabelled Fe^2+^ by Xcc 8004, Δ*xibR*, and strains harboring the plasmid containing either the wild type *xibR* allele (pSSP30) or a point mutant of *xibR* in the putative conserved aspartate residue phosphorylation site (D55AXibR; pSSP39). ^55^FeCl_3_ was reduced to ^55^Fe^2+^ in 1M sodium ascorbate. Uptake assay was performed in the presence of sodium ascorbate to maintain the FeCl_3_ in the reduced form. Data are shown as mean ± S.E. (n=3).

(B and C) Relative quantification of expression of the ferrous iron transporter (*feoB*) and ferric uptake regulator (*fur*) of Xcc by real-time qRT-PCR. RNA was isolated from Xcc 8004, Δ*xibR* and strain harboring the plasmid containing the wild type *xibR* allele (pSSP30) grown under PS, PS + 100 µM DP and PS + 100 µM DP + 100 µM FeSO_4_. 16S ribosomal RNA was used as an endogenous control to normalize the RNA for cellular abundance. Data are shown as mean ± S.E. (n=3), ns = not significant.

(D) Absorbance at 600 nm of Xcc strains grown in PS broth with or without 0.5 μg/ml SNG and 0.01M sodium citrate after 16 and 42 h of growth are shown. Data are shown as mean ± S.E. (n=3).

(E-G). The growth of Xcc 8004, Δ*xibR*, Δ*xibR*/pSSP30 and Δ*xibR*/pSSP39 strains in rich PS medium (E), low iron medium (PS + intracellular ferrous iron chelator 150 µM 2′2′-bipyridyl) (F), and low-iron medium supplemented with iron (PS + 150 µM 2′2′-bipyridyl + 100 µM FeSO_4_) (G). Growth was monitored by determining the OD_600_. Data are shown as mean ± S.E. (n=3).

(H and I) Relative quantification of expression of the putative ferritin-like protein (XC_3752) and periplasmic iron dicitrate sensor (XC_0557) of Xcc by real-time qRT-PCR. RNA was isolated from Xcc 8004, Δ*xibR* and strain harboring the plasmid containing the wild type *xibR* allele (pSSP30) grown in rich PS media, PS + 100 µM DP and PS + 100 µM DP + 100 µM FeSO_4_. 16S ribosomal RNA was used as an endogenous control to normalize the RNA for cellular abundance. Data are shown as mean ± S.E. (n=3). ** Indicating p-value < 0.01 statistical significance by paired student t-test. ns = not significant.

(J-L) The growth of Xcc 8004, Δ*fhuE* Δ*XC_0925*, Δ*fecR* and Δ*yciE* Δ*yciF* Δ*XC_3754* in rich PS medium (J), low-iron medium (PS + intracellular ferrous iron chelator 150 µM 2′2′-dipyridyl) (K), and low-iron medium supplemented with iron (PS + 150 µM 2′2′-dipyridyl + 100 µM FeSO_4_) (L). Growth was monitored by determining the OD_600_. Data is mean of three biological replicates. Error bars are showing SEM.

(M) SNG sensitivity plate assay. Xcc 8004, Δ*fhuE* Δ*XC_0925*, Δ*fecR* and Δ*yciE* Δ*yciF* Δ*XC_3754* were grown in PS media at a density of 1 × 10^9^ cells/ml. 4µL of cultures from each serial dilution was spotted on PSA plates containing 1µg/ml SNG and 0.01M sodium citrate. Plates were incubated for 72 h at 28°C to observe bacterial growth.

(N) Absorbance at 600 nm of Xcc 8004, Δ*fhuE* Δ*XC_0925*, Δ*fecR* and Δ*yciE* Δ*yciF* Δ*XC_3754* grown in PS broth with or without 0.5 μg/ml SNG and 0.01M sodium citrate after 16 and 42 h of growth are shown. Data are shown as mean ± S.E. (n=3).

**Fig. S10. Role of XibR and low-iron condition on the expression of flagellar genes involved in regulation and assembly of different flagellar component.**

(A) Schematic representation of the model of the flagellar transcriptional cascade in Xcc [9]. Expression and assembly of flagellar components takes place in a temporal fashion, in which the Class I protein σ54 and FleQ regulates the expression of class II genes, which are required for site selection and basal body formation. Class III genes encode proteins required for flagellar filament, cap proteins and motility regulatory proteins. Locus tags of flagellar genes encoding proteins are shown in bracket. Based on expression analysis by microarray, genes (locus tags) which are positively regulated by XibR are shown in red color. Genes (locus tags) which are positively regulated by both XibR and low-iron condition are shown as underline. Genes which are not differentially expressed in microarray were depicted in black color.

(B) Quantitative chemotaxis capillary assay with different Xcc strains grown under PS, PS + 100 µM DP and PS + 100 µM DP + 100 µM FeSO_4_. Cells were incubated at 28°C with capillaries containing potassium glutamate (4.9 mM) and PBS. Relative chemotaxis response was determined by migrated bacterial cells in capillary containing potassium glutamate over the capillary containing PBS. Data are shown as mean ± S.E. (n=3). The experiment was repeated two times.

(C) Relative quantification of the expression of *flgD* by real-time qRT-PCR. Different strains of Xcc; Xcc 8004, Δ*xibR* and strain harboring the plasmid containing the wild type *xibR* allele (pSSP30), were grown to OD_600_ 1.2 in PS, PS + 100 µM DP and PS + 100 µM DP + 100 µM FeSO_4_. 16S ribosomal RNA was used as an endogenous control to normalize the RNA for cellular abundance. Data are shown as mean ± S.E. (n=3).

(D) Expression analysis of *flgG* operon in wild-type (Xcc 8004 P*_flgG_*::*_gusA_*) and Δ*xibR* (Δ*xibR*P*_flgG_*::*_gusA_*) grown under PS, PS + 100 µM DP and PS + 100 µM DP + 100 µM FeSO_4_ while monitoring the β-glucuronidase (GUS) activity. Data are shown as mean ± S.D. (n=3).

* Indicating p-value < 0.05, ** Indicating p-value < 0.01 and *** indicating p-value < 0.001 statistically significance by paired student t-test.

**Fig. S11. Cell aggregation phenotype of different strains of Xcc.**

(A) Saturated cultures were grown in rich PS medium and the tubes were kept at room temperature for 4 hours for the observation of aggregation phenotype. Wild-type Xcc 8004 and Δ*xibR*/pSSP30 exhibit disperse phenotype than Δ*xibR* and Δ*xibR*/pSSP39.

(B) Average biofilm thickness of different strains of Xcc formed on the glass slide at the air-media interphase. Different Xcc strains were inoculated in PS, low-iron (PS + 100 µM DP) and iron supplemented (PS + 100 µM DP + 100 µM FeSO_4_) media at a concentration of 10^6^ cells/ml and grown for 24 h. For quantification of the thickness, five independent biofilms were scanned with CLSM at ten randomly selected positions and thickness was determined through height of the biofilm. Data are shown as mean ± S.E. (n=3).

**Fig. S12. Siderophore production by Xcc strains on on PSA-CAS plate with 75 µM DP.**

(A) wild-type Xcc 8004, Δ*xibR*, Δ*xibR*/pSSP30 and Δ*xibR*/pSSP80

(B) Strains wild-type Xcc 8004, Δ*xibR*, Δ*xibR*/pSSP30 and Δ*xssA*/pSSP81

(C) SDS-PAGE for purified XibR with C-terminal His-tag; lane 1 is Unstained Protein MW Marker (ThermoFisher Scientific, Waltham, MA, USA), lane 2, 3, and 4 are different fractions of purified XibR.

(D) Western blot for XibR with C-terminal His-tag using anti-His antibody. Lane 1= Pierce™ Prestained Protein MW Marker (ThermoFisher Scientific, Waltham, MA, USA); lane 2 = un-induced XibR in bl21 (DE3); and lane 3= induced XibR in bl21 (DE3).

**Fig. S13.** Electrophoretic mobility shift assay (EMSA) showing binding of XibR to a ^32^P-labeled *motA* probe with increasing concentration of either ferric (B) or ferrous (D) form of iron.

(A) EMSA showing binding of XibR to a ^32^P-labeled *xss* (-188 to +205) probe. More DNA-protein binary complex was observed with the increase in the concentration of XibR protein.

(C) EMSA showing binding of XibR to a ^32^P-labeled *motA* probe in the presence of other divalent metal ions and ferric iron. Presence of deferoxamine mesylate with FeSO_4_ decreased binding of XibR to the *motA* probe (lane 3).

**Fig. S14.** *In silico* analysis of consensus sequence motifs in promoter of *flg*, *mot* and *xss* operons**. (**A) Sequence logos for the five consensus motifs identified by MEME. (B) Schematic representation of relative position of conserved motifs (shown in red, blue, green, magenta and orange color boxes) on the *flg*, *mot* and *xss* promoter sequences.

**Fig. S15. Exogenous iron supplementation rescued the siderophore overproduction**

**phenotype of the Δ*xibR* mutant.**

Different strains of Xcc were grown on PSA-CAS medium containing without or with 50 μM 2,2′-dipyridyl (DP). For iron supplementation, either FeCl_3_ or FeSO_4_ were added in PSA-CAS + DP medium at a concentration of 10 and 20 μM.

**Supporting References**

1. Larsen R, Wilson M, Guss A, Metcalf W. Genetic analysis of pigment biosynthesis in *Xanthobacter autotrophicus* Py2 using a new, highly efficient transposon mutagenesis system that is functional in a wide variety of bacteria. Arch Microbiol. 2002;178: 193–201. doi:10.1007/s00203-002-0442-2

2. Yao-Guang Liu, Mitsukawa, Teruko Oosumi RFW. Efficient isolation and mapping of *Arabidopsis thaliana* T-DNA insert junctions by thermal asymmetric interlaced PCR. Plant J. 1995;8: 457–463.

3. Schäfer A, Tauch A, Jäger W, Kalinowski J, Thierbach G, Pühler A. Small mobilizable multi-purpose cloning vectors derived from the *Escherichia coli* plasmids pK18 and pK19: selection of defined deletions in the chromosome of *Corynebacterium glutamicum*. Gene. 1994;145: 69–73.

4. Oke V, Long SR. Bacterial genes induced within the nodule during the Rhizobium - legume symbiosis. Mol Microbiol. 1999;32: 837–849.

5. Miller WG, Leveau JHJ, Lindow SE, Biology M, Hall K. Improved gfp and inaZ Broad-Host-Range Promoter-Probe Vectors. Mol Plant Microbe Interact. 2000;13: 1243–1250.

6. Jefferson RA, Kavanagh TA, Bevan MW. GUS fusions: beta-glucuronidase as a sensitive and versatile gene fusion marker in higher plants. EMBO J. 1987;6: 3901–7. doi:10.1073/pnas.1411926112

7. Bailey TL, Elkan C. Fitting a Mixture Model by Expectation Maximization to Discover Motifs in Bipolymers. Proc Second Int Conf Intell Syst Mol Biol. 1994;2: 28–36. doi:citeulike-article-id:878292

8. Fujita MJ, Kimura N, Sakai A, Ichikawa Y, Hanyu T, Otsuka M. Cloning and heterologous expression of the vibrioferrin biosynthetic gene cluster from a Marine Metagenomic Library. Biosci Biotechnol Biochem. 2011;75: 2283–2287. doi:10.1271/bbb.110379.

9. Yang TC, Leu YW, Chang-Chien HC, Hu RM. Flagellar biogenesis of *Xanthomonas campestris* requires the alternative gigma factors RpoN2 and FliA and is temporally regulated by FlhA, FlhB, and FlgM. J Bacteriol. 2009;191: 2266–2275. doi:10.1128/JB.01152-08
