# Supplementary material for: Co-regulation of Iron Metabolism and Virulence Associated Functions by Iron and XibR, a Novel Iron Binding Transcription Factor, in the Plant Pathogen Xanthomonas
Source: PLoS Pathog. 2016 Nov 30;12(11):e1006019. doi: 10.1371/journal.ppat.1006019 (PMC5130282; doi:10.1371/journal.ppat.1006019)
Supplement: S1 Table — (DOC) [file ppat.1006019.s002.doc]

**Table S1. Insertional mutants of *Xanthomonas campestris* pv. *campestris*** with altered siderophore production.

| Locus of Tn5 insertion | Function of gene product | No. of individual transposon hits | Mutant strain | Predicted size, aa | Insertion site, aa | Siderophore halo to colony diameter ratio±SD# | EPS production  (μg/cell) |
| --- | --- | --- | --- | --- | --- | --- | --- |
| ------------ | Wild Type | - | Xcc8004 | --- | --- | 0.26±0.03 | 7.31±0.91 |
| ------------ | Positive control | - | Xom R | --- | --- | 2.06±0.09** | ---- |
| XC_0486 | CAP-like protein | 1 | G12 | 230 | 15 | 0.69±0.04** | 10.63±1.25 |
| XC_1015/  *thiG* | Thiazole synthase | 1 | I3 | 264 | 76 | 1.50±0.15** | 0.12±0.03* |
| XC_1311 | DNA-directed RNA polymerase subunit N | 1 | M5 | 478 | 278 | 1.64±0.30** | 5.11±1.36 |
| XC_1360 | Putative ABC transporter  permease | 1 | M3 | 218 | 191 | 1.33±0.0.52 | 5.09±0.81 |
| XC_1660 | GumD protein | 3 | P2, R13, B12 | 484 | 14, 306,  343 | 1.89±0.17** | 0.17±0.04* |
| XC_1667 | GumK protein | 1 | F15 | 400 | 289 | 0.76±0.07** | 0.02±0.001* |
| XC_2095 | Putative rhodanese | 1 | O28 | 253 | 213 | 0.60±0.15 | 6.69±0.23 |
| XC_2252 | Response regulator | 1 | A11 | 127 | 110 | 0.58±0.13* | 7.57±0.46 |
| XC_2430 | Predicted transcriptional regulator | 1 | S6 | 304 | 277 | 0.53±0.11* | 7.56±1.53 |
| XC_2523 | Putative SNARE associated Golgi Protein | 3 | B3, A2, P1/R6 | 204 | 17, 103,  132 | 1.39±0.50* | 2.24±1.73* |
| XC_2931 | Hypothetical protein | 1 | I22 | 61 | 22 | 0.71±0.07** | 9.17±0.74 |
| XC_3263 | ATP-dependent serine proteinase La | 1 | R5 | 823 | 507 | 0.47±0.03* | 6.09±0.56 |
| XC_3608 | Phosphoglucomutase; phosphomannomutase | 3 | E13, B5, UN | 450 | 320, 343, 384 | 2.53±0.15** | 0.01±0.0006* |
| XC_3609 | Phosphomannose isomerase/GDP-mannose pyrophosphorylase | 1 | S8 | 467 | 79 | 1.23±0.37* | 0.04±0.012* |
| XC_3621 | Hypothetical protein | 1 | B4 | 730 | 92 | 0.87±0.21* | 5.99±0.82 |
| XC_3626 | UDP-glucose 4-epimerase | 1 | H24 | 312 | 127 | 1.05±0.07** | 5.35±0.76 |
| XC_3627 | Putative GDP-mannose 4, 6-dehydratase | 1 | D38 | 344 | 303 | 0.91±0.18* | 7.40±3.47 |
| XC_3630 | Glycosyl transferase | 1 | C8 | 359 | 112 | 0.87±0.22* | 8.05±1.8 |
| XC_3759 | Hypothetical protein | 1 | M4 | 64 | 32 | 0.92±0.13** | 5.39±1.59 |
| **XC_3760** | **Transcriptional regulator NtrC family** | **3** | **M2, M1, B1** | **433** | **9, 79, 425** | **2.61±0.60**** | **5.60±0.28** |
| XC_4297 | Toluene tolerance protein | 1 | S5 | 249 | 121 | 0.98±0.15** | 1.57±0.05* |
| XC_4386, XC_4393 | 16S ribosomal RNA | 1 | T10 | 1539bp | 1379bp | 1.22±0.31* | 6.04±2.24 |
| XC_0689-0690 | Transcriptional regulator-sugar kinase | 1 | T7 | IG | 828751CN | 0.87±0.10** | 6.18±0.54 |
| XC_4342-1658 | tRNA-Pro-GGG-GumB | 1 | B15 | IG | 1993599CN | 0.85±0.23* | 2.17±0.13* |
| XC_3087-3086/*lspA* | isoleucyl-tRNA Synthetase- lspA/signal peptidase II | 1 | G29 | IG | 3697136CN | 0.64±0.28 | 0.54±0.01* |
| XC_3342/  *tuf*-3341 | Elongation factor Tu-30S ribosomal protein | 1 | M6 | IG | 3985034CN | 1.42±0.14** | 9.40±0.10 |
| XC_4366- XC_R0067 | tRNA-Ser-GGA- SRP_bact | 1 | E7 | IG | 3890142CN | 0.78±0.17* | 4.55±0.58 |

# Means and standard deviation of three biological replicates are shown. Data with ‘*’ and ‘**’ in the same column are significantly different from wild-type strain Xcc 8004 at P <0.01 and P < 0.001(Student’s t-test) respectively. The NtrC family of response regulator (XC_3760), is indicated in bold which has been characterized in this study.
