# Supplementary material for: Co-regulation of Iron Metabolism and Virulence Associated Functions by Iron and XibR, a Novel Iron Binding Transcription Factor, in the Plant Pathogen Xanthomonas
Source: PLoS Pathog. 2016 Nov 30;12(11):e1006019. doi: 10.1371/journal.ppat.1006019 (PMC5130282; doi:10.1371/journal.ppat.1006019)
Supplement: S2 Table — (DOC) [file ppat.1006019.s003.doc]

Table S2. Strains and plasmids used in this study.

| **Strain or plasmids** | **Relevant characteristics** | **References** |
| --- | --- | --- |
| *Xanthomonas oryzae* pv. *oryzae strains*  XomR  *Escherichia coli* strains  DH5α  DH5/λ*pir*  *Xanthmonas campestris* pv. *campestris* strains  Xcc 8004  *xibRM1*  *xibRM2*  *xibRB1*  *xibRM1*/pSSP30  *xibRM2*/pSSP30  *xibRB1*/pSSP30  *xibRNP1*  *xibRNP1*/pSSP30  Δ*xibR*  Δ*glnG*  Δ*xibR*/pSSP30  Δ*glnG*/pSSP34  Δ*xibR*Δ*glnG*  Δ*xibR*Δ*glnG*/pSSP30  Δ*xibR*Δ*glnG*/pSSP34  Δ*xibr*/pSSP39  Δ*xssA*  Δ*xssA*/pAP15  Δ*xibr*Δ*xssA*  Δ*xibr*Δ*xssA*/pAP15  Δ*xibr*/pHM1  Xcc 8004/pHM1  Xcc 8004/pSSP30  Δ*xibR*/pSSP61  Δ*xibR*/pSSP62  Δ*xibR*/pSSP63  Δ*glnG*/pSSP64  Δ*glnG*/pSSP65  Δ*glnG*/pSSP66  Xcc 8004 P*xss*:*:gusA*  Δ*xibR* P*xss*:*:gusA*  Xcc8004/pHM1P*xss*:*:gusA*  Δ*xibr*/pHM1P*xss*:*:gusA*  Xcc 8004 P*xibR*::*gusA*  Xcc 8004 P*motA*:*:gusA*  Δ*xibr* P*motA*:*:gusA*  Xcc 8004 P*flgG*:*:gusA*  Δ*xibr* P*flgG*:*:gusA*  Xcc 8004 P*xss*:*:*pPROBE-GT  Δ*xibr* P*xss*:*:*pPROBE-GT  Plasmids  pPROBE-GT  pVO155  pRL27  pK18mobSacB  pK18mob  pHM1  pSSP29  pSSP30  pSSP31  pSSP34  pSSP39  pSSP50  pSSP51  pSSP61  pSSP62  pSSP63  pSSP64  pSSP65  pSSP66  pSSP80  pSSP81  pAP15  (pET (lac-P/xibR)) | Wild-type, Rif^r^  F′/ endA1 hsdR17 (rk– mk+) supE44 thi-1 recA1 gyrA relA1 f80dlacZDM15 (lacZYA-argF) U169  Φ80*dlacZ*ΔM15 Δ(*lacZYAargF*) U169 *recA1 hsdR17 deoR thi-1 supE44 gyrA96 relA1*/λ*pir*  Wild-type, RifR  *xibR*-47::pRL27; Kan^r^  *xibR*-9::pRL27; Kan^r^  *xibR*-425::pRL27; Kan^r^  *xibRM1*mutant complemented with pSSP30; Kan^r^, Spec^r^  *xibRM2* mutant complemented with pSSP30; Kan^r^, Spec^r^  *xibRB1*mutant complemented with pSSP30; Kan^r^, Spec^r^  *xibR*::pSSP29; Kan^r^ derivative of Xcc8004  *xibRNP1* mutant complemented with pSSP30; Kan^r^, Spec^r^  Deletion of XC_3760 gene  Deletion of XC_0198 gene  Δ*xibR* mutant complemented with pSSP30, Spec^r^  Δ*glnG* mutant complemented with pSSP34, Spec^r^  Double deletion of XC_3760 and XC_0198  Δ*xibR*Δ*glnG* mutant complemented with pSSP30; Spec^r^  Δ*xibR*Δ*glnG* mutant complemented with pSSP34; Spec^r^  Δ*xibr* mutant complemented with pSSP39 which having D55AXibR encoding gene; spec^r^  Deletion of XC_1107  Δ*xssA* complemented with pAP15. Tet^r^  Double deletion of XC_3760 and XC_1107  Δ*xibr* Δ*xssA* mutant complemented with pAP15; Tet^r^  *xibR* mutant with empty expression vector pHM1, Spec^r^  Wild-type Xcc 8004 with empty expression vector pHM1, Spec^r^  Xcc 8004 mutant complemented with pSSP30; Spec^r^  Δ*xibR* mutant complemented with pSSP61; Spec^r^  Δ*xibR* mutant complemented with pSSP62; Spec^r^  Δ*xibR* mutant complemented with pSSP63 ; Spec^r^  Δ*glnG* mutant complemented with pSSP64 ; Spec^r^  Δ*glnG* mutant complemented with pSSP65; Spec^r^  Δ*glnG* mutant complemented with pSSP66; Spec^r^  Chromosomal *gusA* fusion with *xss* promoter in Xcc 8004 background; Kan^r^, Amp^r^  Chromosomal *gusA* fusion with *xss* promoter in Xcc 8004 background; Kan^r^, Amp^r^  Chromosomal *gusA* fusion with *xss* promoter in Xcc 8004/pHM1 background; Spec^r^, Kan^r^, Amp^r^  Chromosomal *gusA* fusion with *xss* promoter in Δ*xibr* /pHM1 background; Spec^r^, Kan^r^, Amp^r^  Chromosomal *gusA* fusion with the *xibR* promoter in the Xcc 8004 background; Kan^r^, Amp^r^  Chromosomal *gusA* fusion with *motA* promoter in Xcc 8004 background; Kan^r^, Amp^r^  Chromosomal *gusA* fusion with *motA* promoter in Δ*xibr* background; Kan^r^, Amp^r^  Chromosomal *gusA* fusion with *flgG* promoter in Xcc 8004 background; Kan^r^, Amp^r^  Chromosomal *gusA* fusion with *flgG* promoter in Δ*xibr* background; Kan^r^, Amp^r^  Xcc 8004 strain carrying *xss* promoter fused with *gfp* in pPROBE-GT vector; Gm^r^  Δ*xibr* strain carrying *xss* promoter fused with *gfp* in pPROBE-GT vector; Gm^r^  Promoter probe vector carrying promoterless *gfp*; Gm^r^  pUC119 derivative carrying promoterless *gusA*; Km^r^ Amp^r^  Tn5-RL27 (KmR-oriR6 K) delivery vector: circularized PCR fragment from pRL23  Suicide plasmid in XCC, Kan^r^  2.661Kb suicide plasmid in XCC, Kan^r^  Broad-host-range cosmid vector, pSa *ori*, Spec^r^  pK18::knockout construct of XC_3760 by single recombination  pHM1::full length gene XC_3760 with promoter, Spec^r^  pK18 mob::full length *xibR*  pHM1::full length XC_0198, Spec^r^  pHM1::D55AXibR encoding full length gene, Spec^r^  pK18mobSacB::deletion construct for gene XC_3760  pK18mobSacB::deletion construct for gene XC_0198  pHM1::full length *xibR* with swapped *glnG* rec domain  pHM1::full length *xibR* with swapped *glnG* σ54 interacting domain  pHM1::full length *xibR* with swapped *glnG* DNA binding domain  pHM1::full length *glnG* with swapped *xibR* rec domain  pHM1::full length *glnG* with swapped *xibR* σ54 interacting domain  pHM1::full length *glnG* with swapped *xibR* DNA binding domain  pHM1:: full length gene XC_3760 with C-terminal HA-tag, Spec^r^  pHM1:: full length gene XC_3760 with C-terminal HIS-tag, Spec^r^  pUFR034 with 38.2-kb genomic insert of *X. oryzae* pv. oryzae containing entire *xss* gene cluster, Tet^r^  pET23a vector containing full length *xibR* with C-terminal His-tag, Amp^r^ | Laboratory collection  Laboratory collection  Miller and Mekalanos 1988  Laboratory collection  This study  This study  This study  This study  This study  This study  This study  This study  This study  This study  This study  This study  This study  This study  This study  This study  This study  This study  This study  This study  This study  This study  This study  This study  This study  This study  This study  This study  This study  This study  This study  This study  This study  This study  This study  This study  This study  This study  This study  This study  Miller et al., 2000  Oke and Long, 1999  Larsen *et al*., 2002  Schafer *et al*. 1994  Pridmore *et al*. 1987  Innes *et al*., 1988  This study  This study  This study  This study  This study  This study  This study  This study  This study  This study  This study  This study  This study  This study  This study  Pandey and Sonti, 2010  This study |

The rif^r^ mutation confers resistance to rifampin; Gm^r^, Spec^r^, Tet^r^, Amp^r^ and Kan^r^ indicate resistance to gentamycin, spectinomycin, tetracycline, ampicilin and kanamycin, respectively. Δ*sntrC,* Δ*rpoN* and Δ*ntrC* are deletion mutants of Xcc background in the genes stand alone *ntrC, rpoN* and *ntrC* respectively.
