# Supplementary material for: Co-regulation of Iron Metabolism and Virulence Associated Functions by Iron and XibR, a Novel Iron Binding Transcription Factor, in the Plant Pathogen Xanthomonas
Source: PLoS Pathog. 2016 Nov 30;12(11):e1006019. doi: 10.1371/journal.ppat.1006019 (PMC5130282; doi:10.1371/journal.ppat.1006019)
Supplement: S3 Table — (DOC) [file ppat.1006019.s004.doc]

**Table S3. *xibR*** positively regulated genes but not influenced by iron starvation.

| **Functional group of genes** | **Locus Tag/gene symbol** | **Product name** | **Microarray**  Fold geomean Mutant | **P-value** |
| --- | --- | --- | --- | --- |
| **Iron related genes** | XC_1108  XC_3752(HP)  XC_0543(HP)  XC_3753(HP)  XC_0123  XC_0405  XC_4311(HP)  XC_2296  XC_1004  XC_3851(HP)  XC_4053  XC_0988  XC_0409 | outer membrane receptor for ferric iron uptake  Putative Ferritin-like protein   Putative Ton B dependent receptor  Putative YciF bacterial stress response protein, ferritin-like iron-binding domain  TonB-dependent receptor  TonB-dependent receptor  Putative tonB-dependent receptor  TonB-dependent receptor  TonB-dependent receptor  Putative Gram-negative bacterial tonB protein  TonB-dependent receptor  TonB-dependent receptor  TonB-dependent receptor | -2.914  -3.175  -1.500  -3.882  -3.023  -1.923  -1.858  -1.552  -1.516  -1.363  -1.216  -1.207  -0.777 | 0.040  0.021  0.063  0.015  0.065  0.0083  0.017  0.018  0.035  0.022  0.024  0.0092  0.0099 |
| **N_2_ Metabolism Related genes** | XC_1854  XC_3957 | Ethanolamine ammonia-lyase small subunit  Nitrogen regulatory protein P-II | -2.441  -0.783 | 0.0036  0.012 |
| **Pathogenicity related genes** | XC_1046  XC_0082  XC_1806  XC_2342/ *rpfI* | Virulence regulator  AtsE protein(attachment to host)  Virulence regulator  Regulatory protein RpfI | -1.476  -1.340  -0.762  -1.035 | 0.058  0.0039  0.032  0.091 |
| **Secretion components**  Type II  Type III  Others | XC_0639  XC_0783  XC_3168(HP)  XC_0126  XC_1514  XC_3171(HP)  XC_0747  XC_1298  XC_2481(HP)  XC_3495(HP)  XC_3379  XC_4179  XC_3564  XC_3565 | cellulase  cellulase S  Putative glycosyl hydrolase  pectate lyase E  extracellular protease  Putative glycosyl hydrolase  Type II secretion system protein L  Pectate lyase II  Putative glycosyl hydralase  Putative glycosyl hydralase  Extracellular protease  General secretory pathway related protein  General secretion pathway protein N  General secretion pathway protein M | -3.566  -2.465  -2.374  -1.951  -1.907  -1.896  -1.461  -1.369  -1.185  -1.111  -0.976  -0.910  -0.860  -0.668 | 0.0054  0.0085  0.013  0.101  0.018  0.019  0.0085  0.159  0.172  0.059  0.00603  0.029  0.0097  0.016 |
| **Flagella biogenesis and regulation** | XC_2239/flgG  XC_2234/flgB  XC_2267/fliM  XC_2277/flhB  XC_2266  XC_2260/fliF  XC_2272  XC_2273  XC_2259  XC_2278/ flhA  XC_2231  XC_2265  XC_3724  XC_4016(HP)  XC_0192(HP)  XC_3776(HP) | flagellar basal body rod protein  flagellar basal body rod protein  flagellar motor switch protein FliM  flagellar biosynthesis protein FlhB  flagellar biosynthesis protein  flagellar MS-ring protein  flagellar biosynthesis  flagellar biosynthetic protein  flagellar protein  flagellar biosynthesis protein FlhA  flagellar protein  flagellar protein  Flagellar motor protein MotA  Putative roadblock/LC7 domain  Putative META domain protein  Putative Flagellin N-methylase | -6.332  -6.159  -5.816  -5.316  -5.236  -5.163  -4.689  -3.581  -3.307  -3.266  -2.928  -2.388  -1.034  -0.958  -0.870  -0.651 | 0.00394  0.0015  0.00247  0.00491  3.50E-04  0.0024  0.0016  0.0049  0.257  0.0058  0.019  0.113  0.017  8.70E-04  0.0014  0.016 |
| **Fimbrial and non fimbrial adhesions**  Nonfimbrial adhesions  Fimbrial adhesions | XC_1359  XC_2317(HP)  XC_2860(HP)  XC_1058 | Twitching motility protein  Putative PilZ/YcgR protein  Putative Spore Coat Protein U domain  Pilin | -2.061  -1.968  -1.475  -0.699 | 0.023  0.013  0.021  0.190 |
| **Extracellular Polysaccharides** | XC_1667  XC_1665 | GumK protein  GumI protein | -1.408  -0.977 | 4.90E-04  0.0012 |
| **Chemotaxis** | XC_2284  XC_2302  XC_1383  XC_2311  XC_2318  XC_1409  XC_2223  XC_3758  XC_1190(HP)  XC_3757 | chemotaxis related protein, CheA2  chemotaxis response regulator  Response regulator (Response regulator containing a CheY-like receiver domain and a GGDEF domain)  Chemotaxis protein  Chemotaxis protein  Chemotaxis-specific methylesterase  Chemotaxis protein  Response regulator (Response regulator containing a CheY-like receiver domain and a SARP domain)  Putative CheW-like Protein  Methyltransferase | -2.915  -2.906  -2.849  -2.070  -1.684  -1.664  -1.544  -1.384  -1.205  -0.682 | 0.027  0.00602  9.60E-04  0.017  0.016  0.022  0.039  0.025  0.068  0.0075 |
| **Two component system** | XC_1382  XC_2153  XC_3068  XC_3163  XC_2965(HP)  XC_3748  XC_1756  XC_3069  XC_2579  XC_3273  XC_0987  XC_3067  XC_3056  XC_3997  XC_2252  XC_2229  XC_3055  XC_3100 | Sensor histidine kinase  Sensor histidine kinase  Two-component system sensor protein  EAL domain-containing protein  Putative response regulator receiver domain  Putative two-component system regulatory protein  Two-component system sensor protein  Two-component system regulatory protein  Histidine kinase/response regulator hybrid protein  Two-component system sensor protein  Sensor histidine kinase  Histidine kinase/response regulator hybrid protein  Two-component system sensor protein  Two-component system regulatory protein  Response regulator  Histidine kinase  Two-component system regulatory protein  Putative EF-hand, calcium binding motif (calcium sensor) | -2.133  -2.031  -1.841  -1.815  -1.593  -1.578  -1.483  -1.328  -1.307  -1.203  -1.126  -0.968  -0.842  -0.831  -0.806  -0.774  -0.648  -1.083 | 0.056  0.017  0.033  0.016  0.011  0.050  0.024  0.121  0.119  0.039  0.0203  0.019  0.018  0.0011  0.027  0.0402  0.00506  0.0128 |
| **Transcriptional Regulators** | XC_2251  XC_3760  XC_0318  XC_3216  XC_1006  XC_3796  XC_3072  XC_1457  XC_2801  XC_3399  XC_2887(HP)  XC_1649  XC_3747  XC_0891  XC_0522  XC_3070  XC_0830  XC_3151  XC_3071  XC_3738  XC_2974  XC_1745  XC_3141  XC_2204  XC_1752  XC_3175 | RNA polymerase sigma-54 factor  Transcriptional regulator ntrC family  NAD-dependent deacetylase(SIR2)  Propionate catabolism regulatory protein  LacI family transcription regulator  LysR family transcriptional regulator  Positive regulator of sigma-B  Transcriptional regulator  Transcriptional regulator  Regulator of nucleoside diphosphate kinase  Putative Transcriptional regulator padR-like family  Maltose transport gene repressor  Transcriptional regulator protein  Transcriptional regulator  PbsX family transcriptional regulator  Sigma-B negative effector  Transcriptional regulator  LexA repressor  Negative regulator of sigma-B  Transcriptional regulator  RNA polymerase sigma factor RpoE  LacI family transcription regulator  AraC/XylS family transcriptional regulator  TetR family transcriptional regulator  AraC family transcriptional regulator  uid family transcriptional regulator | -2.115  -2.430  -2.268  -2.167  -1.880  -1.737  -1.517  -1.429  -1.403  -1.353  -1.216  -1.191  -1.182  -1.181  -1.115  -1.088  -0.989  -0.921  -0.915  -0.822  -0.772  -0.767  -0.715  -0.697  -0.655  -0.652 | 9.07E-3  5.70E-5  0.020  0.036  0.049  0.0085  0.016  0.0087  0.027  0.012  0.041  0.076  0.0038  0.192  0.022  0.0064  0.031  0.086  0.042  0.015  0.073  0.049  0.0096  0.025  0.025  0.025 |
| **Small nucleotide binding proteins** | XC_0420(HP)  XC_4313(HP)  XC_2795(HP) | Putative GGDEF domain protein  Putative GGDEF domain protein  Putative GGDEF domain protein | -2.812  -0.893  -0.749 | 1.40E-04  0.066  0.014 |
| **Membrane proteins Transporters and efflux pump** | XC_2301(HP)  XC_1682  XC_0089(HP)  XC_2127(HP)  XC_0305  XC_0417  XC_1777  XC_2711/pstB  XC_1493(HP)  XC_3787(HP)  XC_1776  XC_4064  XC_3996(HP)  XC_2460  XC_3540(HP)  XC_2709  XC_2708  XC_1760  XC_1764  XC_2935(HP)  XC_2923  XC_4169  XC_3730 | Putative sulphur transporter  outer membrane lipoprotein  Putative sugar utilizing protein  Putative ABC-type nickel/oligopeptide-like import system  xanthine/uracil permease  ABC transporter substrate binding protein  Polyamine transport protein  Phosphate transporter ATP-binding protein  Putative transmembrane protein  Putative ABC-2 type transporter  ATP-binding component of putrescine transport system  Heavy metal transporter  Putative ThuA like protein  Sodium/glucose cotransport protein  Putative porin  ABC transporter phosphate permease  ABC transporter phosphate binding protein  Integral membrane transporter  Metabolite transport protein  Putative Mechanosensitive ion channel  MFS transporter  Cation symporter  ABC-2 type transporter | -4.771  -2.967  -2.736  -2.532  -2.002  -1.872  -1.809  -1.625  -1.577  -1.415  -1.403  -1.390  -1.265  -1.166  -1.160  -1.146  -1.132  -0.912  -0.910  -0.882  -0.789  -0.636  -0.804 | 8.50E-04  3.40E-5  0.0072  0.012  0.023  0.010  0.017  0.019  0.018  0.122  0.021  0.0077  0.069  0.0031  0.050  0.042  0.113  0.195  0.0072  0.017  0.0073  0.018  0.015 |
| **Energy and metabolism**  Nucleic acid metabolism and tRNA  Carbohydrate metabolism  Protein/amino acids metabolism    Fatty acid and lipid metabolism  Coenzyme/Secondary metabolism | XC_1559(HP)  XC_0304  XC_0430(HP)  XC_2186  XC_1800  XC_0049(HP)  XC_2499  XC_4199(HP)  XC_1480  XC_0303  XC_0792(HP)  XC_1808  XC_0322/purU  XC_4369  XC_2254  XC_1228/mazG  XC_1728/ upp  XC_4354  XC_3184  XC_2651(HP)  XC_4351  XC_4335  XC_1214  XC_2458  XC_1215(HP)  XC_1219  XC_3885  XC_1217  XC_3415  XC_1003  XC_3050  XC_3487  XC_1645  XC_0127(HP)  XC_4204  XC_1002  XC_2482  XC_3400  XC_0150  XC_4168  XC_3051  XC_0840  XC_0098  XC_0422  XC_4065  XC_0160  XC_2151  XC_0162 (HP)  XC_3173(HP)  XC_2149  XC_4154  XC_0141  XC_0553  XC_0142  XC_1075  XC_1223  XC_1292  XC_2163  XC_3280  XC_1230(HP)  XC_3481(HP)  XC_2929  XC_3969(HP)  XC_2162(HP)  XC_0838  XC_1715  XC_0530  XC_0837  XC_1007  XC_0839  XC_1048  XC_2854/dapD  XC_3496  XC_3722  XC_0302  XC_4051  XC_1231(HP)  XC_3174(HP)  XC_3749  XC_3402  XC_0643  XC_1232/ gcvT  XC_1357(HP)  XC_0097  XC_2255(HP)  XC_1269/ aroK  XC_3290/tdh  XC_0829  XC_0764  XC_0484/trpC  XC_3165  XC_4294  XC_0231  XC_0081  XC_2187(HP)  XC_3450(HP)  XC_0276  XC_0320  XC_4152  XC_1452  XC_3762  XC_3763  XC_3766  XC_4158  XC_2825  XC_1581(HP)  XC_2195  XC_0319  XC_2174  XC_1519  XC_0841  XC_4037  XC_3139  XC_4262  XC_2102/phhB  XC_0726  XC_0328  XC_3943  XC_1957  XC_3493  XC_2961  XC_3774  XC_0092 | Putative nudix hydrolase  adenosine deaminase  Putative Pyridine nucleotide-disulphide oxidoreductase  Exodeoxyribonuclease III  3-methyladenine DNA glycosylase  Putative 2H-phosphodiesterases  Phosphomethylpyrimidine kinase  Putative type I restriction enzyme R protein N terminus (HSDR_N)  Ribonuclease activity regulator protein RraA  Nucleoside hydrolase  Putative Amidohydrolase  ATP-dependent DNA ligase  Formyltetrahydrofolate deformylase  Asp tRNA  Nucleotide sugar transaminase  Nucleoside triphosphate pyrophosphohydrolase  Uracil phosphoribosyltransferase  Leu tRNA  Putative Mrr restriction endonuclease  Putative type II restriction enzyme  Gly tRNA  Met tRNA  Beta-galactosidase  Mannan endo-1,4-beta-mannosidase  Putative alpha-1,2-mannosidase  beta-hexosaminidase  dehydrogenase  glucan 1,4-beta-glucosidase  glucose-fructose oxidoreductase  glycosyl hydrolase  aldose 1-epimerase  alpha-amylase  alpha-glucosidase  Putative Concanavalin A-like lectin/glucanases  Ring canal kelch-like protein  Alpha-glucosidase  Sialic acid-specific 9-O-acetylesterase  Transaldolase B  L-fucose dehydrogenase  NdvB  Alpha-L-arabinosidase  Acetolactate synthase 2 catalytic subunit  Fructose-1,6-bisphosphatase  Glycogen branching enzyme  Beta-xylosidase  5-keto-4-deoxyuronate isomerise  L-sorbosone dehydrogenase  Putative Ribose/Galactose Isomerase  Putative sugar kinase  Succinoglycan biosynthesis protein  Xylosidase  Alpha-amylase  Gluconolactonase precursor  Trehalose synthase  Glucose dehydrogenase  Glucokinase  Endoproteinase Arg-C  Protein-glutamate methylesterase  Peptidyl-Asp metalloendopeptidase  Putative HflC  Putative Dipeptidyl aminopeptidases/acylaminoacyl-peptidases  Peptidyl-prolyl cis-trans isomerase  Putative SPFH domain(Protease)  Putative peptidase M14-like protein  Threonine dehydratase  Peptidase  3-dehydroquinate dehydratase  2-isopropylmalate synthase  Aminopeptidase  Acetolactate synthase isozyme II small subunit  Ribosomal protein S6 modification protein  2,3,4,5-tetrahydropyridine-2,6-carboxylate N-succinyltransferase  5,10-methylenetetrahydrofolate reductase  Methionine sulfoxide reductase B  Amidase  Dipeptidyl peptidase IV  Putative NfeD like protein  Putative histidine phosphatase  D-amino acid oxidase  Methionine sulfoxide reductase  Aminopeptidase  Glycine cleavage system aminomethyltransferase T  Putative alanine racemase  TldD protein(putative protease)  Putative ATP-dependent carboxylate-amine ligase  Shikimate kinase  L-threonine 3-dehydrogenase  L-isoaspartate protein carboxylmethyltransferase  Thiol:disulfide interchange protein  Indole-3-glycerol-phosphate synthase  thioredoxin reductase  Acetyltransferase  Acetyltransferase  Esterase  Putative lipoproteins  Putative SGNH hydrolase  Lipase  Dehydrogenase  Cytochrome C biogenesis protein  Formate dehydrogenase  Cyanide insensitive terminal oxidase  Cyanide insensitive terminal oxidase  Oxidoreductase  2-hydroxyhepta-2,4-diene-1, 7-dioateisomerase/5-carboxymethyl-2-oxo-hex-3-ene-1, 7-dioatedecarboxylase  Glutathione peroxidase-like protein  Putative cytochrome C oxidase subunit II  Molybdopterin biosynthesis protein MoeB  FMN oxidoreductase  Uroporphyrin-III C-methyltransferase  Alkaline phosphatise  Ketol-acid reductoisomerase  Catalase precursor  Catalase  Short chain dehydrogenase  Pterin-4-alpha-carbinolamine dehydratase  Oxidoreductase  NADH-dependent FMN reductase  Bifunctional phosphopantothenoylcysteine decarboxylase/phosphopantothenate synthase  CDP-diacylglycerol-serine o-phosphatidyltransferase  Quinone reductase  Polyketide synthase  Zn-dependent alcohol dehydrogenase  Methanol dehydrogenase regulator | -2.498  -1.821  -1.633  -1.587  -1.550  -1.367  -1.334  -1.142  -1.098  -1.053  -0.988  -0.980  -0.979  -0.911  -0.851  -0.851  -0.844  -0.828  -0.773  -0.718  -0.956  -0.654  -3.778  -3.452  -3.406  -3.084  -2.884  -2.603  -2.577  -2.535  -2.390  -2.310  -1.971  -1.919  -1.775  -1.734  -1.666  -1.532  -1.507  -1.423  -1.392  -1.340  -1.202  -1.171  -1.167  -1.078  -1.037  -1.034  -1.026  -0.947  -0.871  -0.871  -0.799  -0.733  -0.718  -0.647  -4.578  -3.107  -2.019  -1.759  -1.691  -1.659  -1.641  -1.523  -1.508  -1.495  -1.387  -1.309  -1.308  -1.293  -1.246  -1.223  -1.211  -1.164  -0.982  -0.958  -0.890  -0.883  -0.868  -0.818  -0.796  -0.760  -0.731  -0.729  -0.720  -0.702  -0.694  -0.680  -0.652  -0.639  -1.812  -3.018  -1.580  -1.537  -1.520  -1.177  -1.065  -0.691  -6.780  -2.959  -1.997  -1.911  -1.626  -1.452  -1.384  -1.336  -1.244  -1.217  -1.163  -1.073  -0.974  -0.967  -0.966  -0.953  -0.924  -0.841  -0.788  -0.751  -0.736  -0.726  -0.668  -1.329  -1.326 | 0.0022  0.088  0.018  0.049  0.014  0.193  0.016  0.087  0.051  0.106  0.064  0.039  0.059  0.074  0.0078  0.031  0.0027  0.150  0.027  0.0041  0.053  0.349  0.0042  0.0017  4.30E-04  0.015  0.0026  0.139  0.0013  0.014  0.021  0.0037  0.047  0.019  0.005  2.50E-04  0.029  0.025  0.049  0.060  0.162  0.029  0.0021  0.0012  0.00307  0.019  0.087  0.073  0.029  0.075  0.036  0.043  0.098  0.022  0.0053  0.012  5.40E-04  0.012  0.014  0.014  0.0012  0.0079  0.023  0.0047  0.080  0.088  0.087  0.027  0.075  0.0036  0.012  0.0046  0.072  0.0062  0.167  0.014  0.014  0.052  0.0199  0.0065  0.144  0.018  0.029  0.040  0.0085  0.011  0.021  0.0081  0.0045  0.150  0.014  0.020  0.022  0.065  0.025  0.025  9.30E-04  8.02E-04  0.014  0.00402  0.0039  0.056  0.0067  0.0027  0.012  0.071  0.012  0.023  0.013  0.065  0.012  0.023  0.043  0.021  0.069  0.329  0.039  0.128  0.00304  0.042  0.015  0.120 |
| **Stress Response** | XC_0407  XC_1289(HP)  XC_1243  XC_0644  XC_1400(HP)  XC_0755  XC_2401(HP)  XC_4010  XC_3199  XC_1852(HP)  XC_0274  XC_0598(HP)  XC_1317(HP)  XC_3999  XC_0045  XC_0734(HP)  XC_4260(HP)  XC_2764 | Superoxide dismutase  Putative stress induced protein (KGG)  Heat shock protein  Sulfur deprivation response regulator  Putative Osmotically inducible protein C (OsmC)  Nisin-resistance protein  Putative abortive infection C-terminus (bacteriophage resistance)  Beta-lactamase related protein  Low molecular weight heat shock protein  Putative metallo-beta-lactamase protein  Organic hydroperoxide resistance protein  Putative Glyoxalase/Bleomycin resistance protein/Dioxygenase  Putative BolA-like protein  Acriflavin resistance protein  Methicillin resistance protein  Putative VanZ protein  Putative YdeI or OmpD-associated  Heat shock protein GrpE | -1.309  -4.123  -2.832  -2.089  -1.730  -1.443  -1.030  -0.939  -0.916  -0.832  -0.822  -0.772  -0.757  -0.753  -0.735  -0.677  -0.669  -0.664 | 0.0073  0.0057  0.143  0.065  0.013  0.061  0.023  0.0047  0.019  0.019  0.011  0.014  0.153  0.037  0.068  0.0011  0.01007  0.035 |
| **Replication and maintenance** | XC_3967  XC_2299  XC_1286(HP)  XC_2577(HP)  XC_1758(HP)  XC_1355  XC_4203  XC_3520(HP)  XC_1332  XC_2400  XC_3166(HP)  XC_3821  XC_0109 | DNA polymerase-related protein  chromosome partioning protein  Putative JmjC domain, hydroxylase  Putative RecA-superfamilyATPases  Putative chromosome segregation protein SMC  Histone-like protein  Histone  Putative post-segregation antitoxin CcdA protein  DNA transport competence protein  DNA helicase related protein  Putative Alkylated DNA repair protein  DNA processing chain A  ATP-dependent DNA ligase | -2.016  -1.951  -1.352  -1.348  -0.994  -0.977  -0.956  -0.780  -0.706  -0.977  -1.642  -1.155  -1.754 | 0.100  0.018  0.020  0.00603  0.071  0.015  0.069  0.051  0.042  0.064  0.035  0.012  0.031 |
| **Cell wall biogenesis** | XC_3172  XC_3767  XC_4130/gidB  XC_1354  XC_1389 | Glycosyl hydrolase  UDP-glucose 4-epimerase  Glucose-inhibited division proteinB  Peptidase(cell envelop biogenesis)  Putative glycosyltransferases | -1.452  -1.256  -1.196  -0.998  -0.864 | 0.0021  0.0018  0.063  0.101  0.092 |
| **Phage related Proteins** | XC_2421 | Phage-related integrase | -2.235 | 0.0047 |
| **Hypothetical Proteins** | XC_4153  XC_3761  XC_3756  XC_1453  XC_2437  XC_3759  XC_4035  XC_3661  XC_1068  XC_3755  XC_3765  XC_2420  XC_0406  XC_1353  XC_2505  XC_2861  XC_3695  XC_0129  XC_3750  XC_1288  XC_1044  XC_2418  XC_3696  XC_1362  XC_3169  XC_4217  XC_2439  XC_1045  XC_0986  XC_2583  XC_1352  XC_3595  XC_0129  XC_4295  XC_2926  XC_1040  XC_2249  XC_2094  XC_1954  XC_4307  XC_1019  XC_3717  XC_2937  XC_2963  XC_4269  XC_3142  XC_3775  XC_0128  XC_1557  XC_2898  XC_2821  XC_1709  XC_0466  XC_1859  XC_2794  XC_1558  XC_1351  XC_1052  XC_0074  XC_1583  XC_0934  XC_3971  XC_3977  XC_2792  XC_3784  XC_1804  XC_2582  XC_1805  XC_1967  XC_3494  XC_0973  XC_3152  XC_3497  XC_0650  XC_3417  XC_2662  XC_1157  XC_3299  XC_0329  XC_0180 XC_3291 XC_3831 XC_3777  XC_3871  XC_3716  XC_1932  XC_0935  XC_2446 | HP  HP  HP  HP  HP  HP  HP  HP  HP  HP  HP  HP  HP  HP  HP  HP  HP  HP  HP  HP  HP  HP  HP  HP  HP  HP  HP  HP  HP  HP  HP  HP  HP  HP  HP  HP  HP  HP  HP  HP  HP  HP  HP  HP  HP HP HP HP HP HP HP  HP HP HP HP HP HP  HP  HP  HP  HP  HP  HP  HP  HP  HP HP HP  HP HP HP  HP  HP  HP  HP  HP  HP  HP  HP  HP  HP  HP  HP  HP HP HP HP HP HP | -3.406  -3.179  -3.168  -2.729  -2.248  -2.134  -1.997  -1.976  -1.974  -1.956  -1.951  -1.937  -1.788  -1.697  -1.673  -1.642  -1.639  -1.572  -1.557  -1.508  -1.431  -1.410  -1.395  -1.392  -1.389  -1.369  -1.367  -1.360  -1.357  -1.273  -1.232  -1.219  -1.177  -1.164  -1.161  -1.149  -1.150  -1.148  -1.144  -1.120  -1.100  -1.086  -1.082  -1.066  -1.039  -1.039  -1.020  -1.018  -1.016  -1.015  -1.003  -1.001  -0.986  -0.971  -0.956  -0.941  -0.940  -0.937  -0.934  -0.921  -0.908  -0.905  -0.891  -0.890  -0.885  -0.878  -0.874  -0.868  -0.861  -0.840  -0.836  -0.804  -0.800  -0.794  -0.794  -0.794  -0.789  -0.785  -0.769  -0.767  -0.742  -0.735  -0.728  -0.728  -0.702  -0.687  -0.672  -0.645 | 0.0078  0.00516  0.00334  0.0059  0.039  0.010  0.0036  0.00501  0.015  0.124  0.016  0.013  0.014  0.023  0.035  0.034  0.012  0.035  0.0094  0.016  0.066  0.0058  0.029  0.050  2.60E-04  0.0061  0.054  0.0092  0.0053  0.0074  0.106  0.0053  0.0052  0.012  0.077  0.108  0.0082  0.018  0.054  0.093  0.072  0.148  0.119  0.029  0.161  0.067  0.0063  0.023  0.033  5.80E-04  0.0015  0.0099  0.029  0.151  0.0058  0.0087  0.031  0.041  0.00902  0.00402  0.015  0.038  0.014  0.024  0.011  0.106  0.043  0.036  0.056  0.036  0.104  0.033  0.0089  6.05E-04  0.0026  0.120  0.0015  0.054  0.111  0.018  0.0095  0.017  0.068  0.0297  0.013  0.018  0.027  0.015 |
| **Others** | XC_4293  XC_4292  XC_0130(HP)  XC_4291  XC_1285(HP)  XC_2936(HP)  XC_0131(HP)  XC_1399  XC_3418(HP)  XC_2545  XC_1495  XC_1670(HP)  XC_4036  XC_2939(HP)  XC_1366  XC_3167  XC_0994  XC_2374  XC_2962(HP) | Microcystin dependent protein  Microcystin dependent protein  Putative KTSC domain  Microcystin dependent protein  Putative SapC  Putative prokaryotic dksA/traR C4-type zinc finger  Putative bacterial Tol like receptor  Pirin  Putative EF-hand, calcium binding motif  Putative luciferase  Thioredoxin  Putative cupin  Ankyrin-like protein  Putative metal-dependent hydrolase  Holliday junction resolvase-like protein  Short chain dehydrogenase  Bifunctional sulfate adenylyltransferase subunit 1/adenylylsulfate kinase protein  Bifunctional phosphoribosyl-AMP cyclohydrolase/phosphoribosyl-ATP pyrophosphatase protein  Putative adenylate cyclase class IV | -2.679  -2.614  -2.253  -2.137  -1.498  -1.235  -1.091  -1.075  -1.075  -1.060  -1.038  -0.992  -0.919  -0.844  -0.632  -1.369  -1.522  -1.391  -0.721 | 0.0012  9.90E-04  0.017  0.034  0.0092  0.041  0.022  0.026  0.0052  0.024  0.076  0.019  0.057  0.068  0.212  0.110  0.015  0.039  0.074 |
