# Supplementary material for: Co-regulation of Iron Metabolism and Virulence Associated Functions by Iron and XibR, a Novel Iron Binding Transcription Factor, in the Plant Pathogen Xanthomonas
Source: PLoS Pathog. 2016 Nov 30;12(11):e1006019. doi: 10.1371/journal.ppat.1006019 (PMC5130282; doi:10.1371/journal.ppat.1006019)
Supplement: S4 Table — (DOC) [file ppat.1006019.s005.doc]

**Table S4. *xibR*** negatively regulated genes but not influenced by iron starvation.

| **Functional group of genes** | **Locus Tag/gene symbol** | **Product name** | **Microarray**  Fold geomean Mutant | **P-value** |
| --- | --- | --- | --- | --- |
| **Iron related genes** | XC_0123 | TonB-dependent receptor | 0.679 | 0.198 |
| **N_2_ Metabolism Related genes** | XC_4180 | glutamine amidotransferase | 0.839 | 0.015 |
| **Pathogenicity related genes** | XC_1027  XC_2227 | VirB6 protein  HrpX related protein | 0.820  0.735 | 0.101  0.0028 |
| **Secretion components**  Type II  Type III  Others | XC_0748 | type II secretion system protein M | 1.088 | 0.148 |
| **Flagella biogenesis and regulation** |  |  |  |  |
| **Fimbrial and non fimbrial adhesions**  Nonfimbrial adhesions  Fimbrial adhesions |  |  |  |  |
| **Extracellular Polysaccharides** |  |  |  |  |
| **Chemotaxis** | XC_1290/cheB | protein-glutamate methylesterase | 4.196 | 0.064 |
| **Two component system** | XC_4236  XC_0496  XC_0250(HP)  XC_2129  XC_4167  XC_2390  XC_3982 | sensor histidine kinase  two-component system regulatory protein  putative Adenylate cyclise  histidine kinase/response regulator hybrid protein  two-component system sensor protein  tryptophan-rich sensory protein  two-component system sensor protein | 1.840  1.649  1.250  1.144  0.815  0.671  0.624 | 0.006  0.001  0.090  0.025  0.078  0.031  0.111 |
| **Transcriptional Regulators** | XC_2906(HP)  XC_2905  XC_0078(HP)  XC_0245  XC_3739  XC_1295 | putative anti-sigma-K factor, RskA  RNA polymerase sigma factor (sigma70)  putative NmrA like protein  AraC family transcriptional regulator  tryptophan repressor binding protein  AhyR/AsaR family transcriptional regulator | 1.083  0.983  0.826  0.779  0.755  0.739 | 0.0021  0.0017  0.040  0.045  0.025  0.032 |
| **Small nucleotide binding proteins** |  |  |  |  |
| **Membrane proteins Transporters and efflux pump** | XC_0762  XC_0157  XC_0269  XC_0820  XC_0388(HP)  XC_4223  XC_4321  XC_1840  XC_1134  XC_3745  XC_2409(HP)  XC_0876(HP)  XC_3477(HP)  XC_0313  XC_1469  XC_1887  XC_1834  XC_4224 | high-affinity choline transport  C4-dicarboxylate transport protein  integral membrane protein  C4-dicarboxylate transporter DctA  putative outer membrane beta barrel protein  OmpA-related protein  Export protein  phosphate-binding protein  potassium uptake protein  amino acid transporter  putative ATP-binding domain of ABC transporters  putative Bestrophin, RFP-TM, chloride channel  putative membrane protein  MFS transporter  inner membrane protein  ABC transporter ATP-binding protein  ABC transporter ATP-binding protein  OmpA-related protein | 2.476  1.900  1.767  1.654  1.643  1.230  1.020  1.018  0.965  0.961  0.854  0.745  0.739  0.694  0.694  0.673  0.659  1.129 | 0.002  0.158  0.168  0.018  7.00E-05  0.0027  0.116  0.0073  0.0018  0.062  5.20E-04  0.022  0.0104  0.030  0.076  0.0024  0.017  0.034 |
| **Energy and metabolism**  Nucleic acid metabolism and tRNA  Carbohydrate metabolism  Protein/amino acids metabolism  Fatty acid and lipid metabolism  Secondary metabolism | XC_2959(HP)  XC_0283  XC_0451(HP)  XC_4289  XC_1328  XC_0509  XC_0575  XC_0574  XC_0122  XC_0292(HP)  XC_1543  XC_0528(HP)  XC_3502  XC_0077  XC_1331  XC_2380/hisD  XC_0483/trpD  XC_0216  XC_0899  XC_3393(HP)  XC_3313  XC_3718/alr  XC_1577/trpA  XC_3719  XC_0035  XC_0586(HP)  XC_4267(HP)  XC_0782(HP)  XC_2331  XC_2980  XC_3652  XC_4263  XC_0760  XC_4080  XC_2979  XC_0206  XC_0029  XC_0380  XC_2978  XC_2977  XC_2678  XC_0229  XC_0254  XC_1135(HP)  XC_0540(HP)  XC_0375  XC_3539  XC_1470(HP)  XC_0482(HP)  XC_0539(HP) | putative Deoxyribodipyrimidine photo-lyase-related protein  pseudouridylate synthase  putative endonuclease  exodeoxyribonuclease V gamma chain  nucleotidyl transferase  phosphoribosylamine--glycine ligase  malonate decarboxylase gamma subunit  malonate decarboxylase subunit beta  2-keto-3-deoxygluconate kinase  putative polysaccharide deacetylase  metallopeptidase  putative 23S rRNA protein  peptidase  metalloprotease  Product: succinyl-diaminopimelate desuccinylase  histidinol dehydrogenase  anthranilate phosphoribosyltransferase  pyruvate dehydrogenase  ribose-5-phosphate isomerase A  Putative Formylglutamate amidohydrolase (FGase)  family II 2-keto-3-deoxy-D-arabino-heptulosonate 7-phosphate synthase  alanine racemase  tryptophan synthase subunit alpha  D-amino acid dehydrogenase small subunit  phospholipid N-methyltransferase  putative lipase 3  putative esterase  putative DegV (fatty acid transport or metabolism)  putative long-chain fatty acyl CoA ligase  acyl-CoA dehydrogenase  beta-ketoacyl-[ACP] synthase I  cardiolipin synthetase  choline dehydrogenase  carbonic anhydrase  enoyl-CoA hydratase  porphyrin biosynthesis protein  alcohol dehydrogenase  protocatechuate 3,4-dioxygenase alpha chain  enoyl-CoA hydratase  3-hydroxyisobutirate dehydrogenase  carbonic anhydrase  NAD(P)H steroid dehydrogenase  biotin carboxylase  putative Dienelactone hydrolase  putative Dienelactone hydrolase  vanillate O-demethylase oxygenase  riboflavin synthase subunit alpha  putative monooxygenase  putative flavin reductase  putative Dienelactone hydrolase | 3.386  2.033  1.162  0.817  0.745  0.662  1.267  0.916  0.688  0.626  2.315  1.146  0.970  0.910  0.764  0.754  0.735  0.720  0.702  0.693  0.692  0.690  0.683  0.667  1.715  1.087  0.985  0.960  0.841  0.737  0.713  0.708  2.257  2.202  2.022  1.931  1.376  1.219  1.142  1.077  1.049  0.961  0.932  0.890  0.874  0.825  0.776  0.768  0.744  0.892 | 0.032  0.249  0.0062  0.0064  0.014  0.013  0.028  0.012  7.70E-04  0.082  0.006  0.187  0.00570  0.059  9.40E-05  0.0059  0.0048  0.095  0.015  0.0049  0.016  0.0055  0.021  0.015  0.007  0.036  0.010  0.016  0.038  0.059  0.070  0.018  0.008  0.003  0.012  0.0033  0.016  0.163  0.00427  0.0093  0.068  0.032  0.0029  0.039  0.015  0.0049  0.042  0.016  5.75E-04  0.020 |
| **Stress Response** | XC_0244(HP)  XC_4256  XC_2429(HP)  XC_1013 | putative SgaA-N-like protein  fusaric acid resistance protein  putative Abi-like protein  sulfur deprivation response regulator | 3.701  0.863  0.777  0.748 | 0.244  0.155  0.024  0.024 |
| **Replication and maintenance** | XC_2065  XC_2071  XC_0538(HP)  XC_4115/uvrD  XC_1817/muL  XC_3985  XC_3242 /*recR*  XC_0947 | single-stranded DNA-binding protein  chromosome partitioning related protein  putative Chromosome segregation ATPases  DNA-dependent helicase II  DNA mismatch repair protein  histone H1  recombination protein  ATP-dependent DNA helicase RecG | 3.539  1.802  0.765  0.759  0.689  0.921  0.866  0.651 | 0.248  0.062  0.059  7.70E-05  0.0069  0.0056  9.50E-04  0.016 |
| **Cell wall biogenesis** | XC_3123/minE  XC_3555(HP)  XC_1732(HP)  XC_0209  XC_0567(HP)  XC_4282(HP)  XC_0771  XC_3874  XC_3122  XC_3235(HP)  XC_3121/minC | cell division topological specificity factor MinE  putative glycosyl transferase  Putative Glycosyltransferase sugar-binding region containing DXD motif  glycosyltransferase  putative nucleoside-diphosphate-sugar epimerase  putative peptidoglycan-binding domain-containing protein [Cell envelope biogenesis, outer membrane]  putative Glycosyl transferases  putative peptidoglycan binding domain  septum site-determining protein  putative Glycosyltransferases  septum formation inhibitor | 0.664  5.446  2.968  2.834  2.702  1.004  0.811  0.805  0.778  0.701  0.665 | 0.197  0.003  0.024  0.004  0.242  0.0043  0.0051  0.0067  0.026  0.012  0.0049 |
| **Phage related Proteins** | XC_2434 | phage associated protein | 1.736 | 0.199 |
| **Hypothetical Proteins** | XC_2781  XC_0561  XC_2615  XC_0736  XC_3554  XC_2135  XC_0634  XC_2933  XC_0207  XC_1711  XC_2055  XC_2407  XC_0076  XC_4283  XC_3955  XC_3388 XC_0497  XC_0620  XC_3909  XC_4029  XC_4245  XC_0870  XC_3109  XC_4039  XC_0464  XC_3124  XC_1549  XC_2406  XC_3110  XC_4040  XC_3217  XC_4175 XC_2932  XC_2017  XC_2532  XC_0291  XC_1083  XC_3131  XC_1401  XC_3835  XC_0860  XC_2614 | HP  HP  HP  HP  HP  HP  HP  HP  HP  HP  HP  HP  HP  HP  HP  HP  HP  HP  HP  HP  HP  HP  HP  HP  HP  HP  HP  HP  HP  HP  HP  HP  HP  HP  HP  HP  HP  HP  HP  HP  HP  HP | 4.898  4.551  3.614  3.538  2.339  1.593  1.264  1.259  1.254  1.199  1.096  1.047  1.041  1.004  0.958  0.902  0.892  0.875  0.844  0.844  0.841  0.826  0.795  0.793  0.785  0.783  0.780  0.778  0.767  0.752  0.748  0.748  0.721  0.703  0.678  0.671  0.667  0.663  0.649  0.647  0.630  3.765 | 0.016  0.063  3.40E-04  0.095  0.162  0.024  0.009  0.131  0.006  0.020  0.018  0.013  0.011  0.087  0.091  0.038  0.020  0.033  0.020  0.072  0.027  0.0032  0.055  0.0052  0.016  0.0065  0.0297  0.023  0.103  0.0062  0.077  0.022  0.010  0.054  0.0059  0.0034  0.0013  0.0094  0.0063  0.00601  0.036  7.05E-03 |
| **Others** | XC_3203(HP)  XC_4085  XC_2408  XC_0685  XC_0223(HP)  XC_2344  XC_0352  XC_3624  XC_2007  XC_2628  XC_1212 | putative CAAX protease self-immunity  ankyrin-like protein  hydroxyproline-rich glycoprotein DZ-HRGP  surface antigen gene  putative Fic family protein  regulatory protein rpfE  xanthomonadin biosynthesis related protein 1  ISxcC1 transposase  IS1478 transposase  ISxcC1 transposase  ISxac3 transposase | 1.396  1.360  0.813  0.709  0.693  0.643  0.680  0.861  0.794  0.762  0.661 | 0.199  0.023  0.016  0.130  0.014  0.0033  0.026  0.069  0.013  0.022  0.071 |
