# Supplementary material for: Co-regulation of Iron Metabolism and Virulence Associated Functions by Iron and XibR, a Novel Iron Binding Transcription Factor, in the Plant Pathogen Xanthomonas
Source: PLoS Pathog. 2016 Nov 30;12(11):e1006019. doi: 10.1371/journal.ppat.1006019 (PMC5130282; doi:10.1371/journal.ppat.1006019)
Supplement: S5 Table — (DOC) [file ppat.1006019.s006.doc]

**Table S5. List of the genes positively regulated by iron starvation but not regulated by Xib*R***.

| **Functional group of genes** | **Locus Tag/gene symbol** | **Product name** | | **Microarray**  Fold geomean WT+DP | **P-value** |
| --- | --- | --- | --- | --- | --- |
| **Iron related genes**  Ferrous  Ferric | XC_2354  XC_0557  XC_2355  XC_4249  XC_0167  XC_1106(HP)  XC_0642  XC_0493  XC_1341  XC_3463  XC_2846  XC_4044  XC_1108  XC_3209  XC_1948 | ferrous iron transport protein B  Putative FecR which involved in regulation of iron dicitrate transport (transmembrane sensor)  ferrous iron transport protein  ferrichrome-iron receptor 3  ferripyoverdine receptor  Putative IucA / IucC family(siderophore production) and Ferric iron reductase FhuF-like transporter  ferripyoverdine receptor  Bacterioferritin-associated ferredoxin  TonB-dependent receptor  outer membrane hemin receptor  iron receptor  iron-uptake factor  citrate-dependent iron transporter  TonB-dependent receptor  iron dependent repressor(IdeR) | | 1.275  6.413  1.684  2.893  2.136  2.508  3.579  1.498  3.194  6.726  4.770  3.423  2.223  1.037  0.820 | 0.051  0.003  0.006  0.006  0.524  0.200  0.004  0.003  0.005  0.018  0.003  0.001  0.009  0.062  0.105 |
| **N_2_ Metabolism Related genes** | XC_0897(HP) | Putative Transglutaminase like protein | | 0.973 | 0.053 |
| **Pathogenicity related genes** | XC_2081  XC_2004  XC_2602  XC_3802  XC_2082  XC_1811  XC_2917 | avirulence protein  avirulence protein  avirulence protein  Avirulence protein  avirulence protein  virulence protein(VirJ)  virulence protein | | 1.176  1.422  1.839  1.755  1.559  0.823  0.638 | 0.035  0.048  0.002  0.003  0.027  0.001  0.00045 |
| **Secretion components**  Type II  Type III  Others | XC_1849  XC_1120  XC_0625  XC_0027  XC_0626  XC_1740(HP)  XC_3021  XC_3001  XC_3003  XC_3004  XC_3005  XC_3006  XC_3007  XC_3008  XC_3009  XC_3010  XC_3011  XC_3025  XC_3015  XC_3020  XC_3019  XC_3013  XC_3018  XC_3016  XC_3023  XC_3076  XC_3017  XC_3014 | Polygalacturonase  xylanase  Cellulose  cellulase  1,4-beta-cellobiosidase  Putative Secretory lipase  HrpE protein  Hpa2 protein  HrcC protein  HrpB8 protein  HrpB7 protein  Type III secretion system ATPase  Type III secretion protein  HrpB4 protein  HrcJ protein  HrpB2 protein  HrpB1 protein  HrpF protein  HrcQ protein  HrpD6 protein  HrpD5 protein  HrcV protein  HpaA protein  HrcR protein  HrpW protein  HrpX protein  HrcS protein  HpaP protein | | 2.535  1.107  2.804  1.760  1.644  1.083  2.013  3.734  2.429  2.319  2.229  2.553  2.801  2.735  2.103  2.480  2.479  3.201  2.318  2.517  3.016  2.237  2.573  2.013  2.151  1.906  2.495  2.222 | 0.002  0.140  0.002  0.003  0.027  0.096  0.048  0.014  0.001  0.006  0.015  0.006  0.006  0.005  0.011  0.025  0.006  0.006  0.008  0.005  0.017  0.005  0.009  0.010  0.032  0.012  0.018  0.017 |
| **Flagella biogenesis and regulation** |  |  | |  |  |
| **Fimbrial and non fimbrial adhesions** |  |  | |  |  |
| **Extracellular Polysaccharides** |  |  | |  |  |
| **Chemotaxis** | XC_1414 | chemotaxis histidine protein kinase,cheA3 | | 4.098 | 0.105 |
| **Two component system** | XC_4022(HP)  XC_0197  XC_0637  XC_1261  XC_2793  XC_0386(HP)  XC_1938 | Putative Ca2+/calmodulin-dependent protein kinases II  two-component system sensor protein  histidine kinase/response regulator hybrid protein  histidine kinase/response regulator hybrid protein  sensor histidine kinase  Putative RIO1(serine/threonine kinase)  two-component system regulatory protein | | 1.524  2.523  1.289  1.095  1.004  0.865  0.766 | 0.018  0.206  0.117  0.230  0.004  0.029  0.021 |
| **Transcriptional Regulators** | XC_2827  XC_0334(HP)  XC_2841  XC_1348  (HP)  XC_0556  XC_3428  XC_1022 | transcriptional regulator marR family  Putative HTH family  transcriptional regulator  Putative transcriptional regulator, BadM/Rrf2 family  RNA polymerase sigma factor(putative sigma 70)  transcriptional regulator gntR family  regulatory protein bphR | | 1.020  1.245  1.104  2.060  4.458  1.150  0.868 | 0.070  0.078  0.020  0.056  1.60E-04  0.015  0.041 |
| **Small nucleotide binding proteins** | XC_0671 | ElaA protein | | 1.206 | 0.167 |
| **Membrane proteins Transporters and efflux pump** | XC_1347  XC_0155  XC_2546  XC_2859  XC_0470  XC_1978 | ABC transporter permease  C4-dicarboxylate transport system  MFS transporter  outer membrane usher protein FasD membrane protein  sugar ABC transporter ATP-binding protein | | 1.189  3.254  1.616  1.107  0.915  0.826 | 0.017  0.085  0.001  0.047  0.135  0.241 |
| **Energy and metabolism**  Nucleic acid metabolism and tRNA  Carbohydrate metabolism  Protein /amino acids metabolism    Fatty acid and lipid metabolism  Secondary metabolism | XC_3161  XC_2417  XC_4274  XC_2742  (fumC)  XC_1109  XC_2984  XC_2985  XC_2990  XC_3676  XC_0360  XC_3192  XC_1340  XC_0112  (HP)  XC_0917(HP)  XC_3895  XC_3427  XC_0208  XC_2824  XC_1111  XC_4326 | excinuclease ABC subunit A  plasmid mobilization protein  integrase  fumarate hydratase  4-hydroxy-2-oxovalerate aldolase  arabinogalactan endo-1,4-beta-galactosidase  beta-galactosidase  Putative Alpha-L-fucosidase  chorismate mutase  transferase  Aminopeptidase N   1. putative hydroxylase 2. Putative 2-oxoglutarate (2OG) and Fe(II)-dependent oxygenase   Putative Isoprenylcysteine carboxyl methyltransferase (ICMT) family  Putative Acetyl-CoA hydrolase  disulphide-isomerase  protocatechuate 3,4-dioxygenase beta chain  uroporphyrinogen-III synthetase  ferredoxin-NADP reductase  Cytochrome P450 hydroxylase  Phosphatase precursor | | 0.877  0.641  0.919  1.096  2.058  3.964  3.520  1.358  1.393  0.951  0.680  3.582  2.017  0.780  1.722  1.003  0.697  1.265  1.222  0.884 | 0.014  0.193  0.072  0.051  0.072  0.003  0.094  0.117  0.013  0.024  0.003  0.008  0.043  0.004  0.036  0.039  0.013  0.002  0.339  0.016 |
| **Stress Response** | XC_0085(HP) | Putative Activator of Hsp90 ATPase homolog 1-like protein | | 0.894 | 0.013 |
| **Replication and maintenance** | XC_1035(HP)  XC_3035 | Putative helicase  DNA mismatch repair protein | | 3.214  1.327 | 0.077  0.021 |
| **Cell wall biogenesis** | XC_1908(HP)  XC_2691(HP) | Putative BolA-like protein  Putative Cyclopropane fatty acid synthase and related methyltransferases [Cell envelope biogenesis | | 0.750  1.919 | 0.005  0.021 |
| **Phage related Proteins** | XC_3916 | phage-related baseplate assembly protein | | 1.644 | 0.002 |
| **Hypothetical Proteins** | XC_3189  XC_0241  XC_3147  XC_2996  XC_2353  XC_3024  XC_0268  XC_2998  XC_3461  XC_0030  XC_0260  XC_1336  XC_0563  XC_0262  XC_1147  XC_1448  XC_2995  XC_3963  XC_0542  XC_2997  XC_0030  XC_1210  XC_2696  XC_4275  XC_2753  XC_4128 | HP  HP  HP  HP  HP  HP  HP  HP  HP  HP  HP  HP  HP  HP  HP  HP  HP  HP  HP  HP  HP  HP  HP  HP  HP  HP | | 0.838  2.513  2.060  2.244  1.294  2.320  1.940  2.166  4.062  1.225  3.880  3.801  1.593  3.048  1.685  2.447  2.318  2.263  2.164  2.011  1.341  0.995  0.826  0.797  0.724  0.694 | 0.012  0.013  0.003  0.005  0.090  0.030  0.009  0.015  0.003  0.049  0.006  0.001  0.050  0.030  0.150  0.075  0.005  0.146  0.008  0.000053  0.032  0.093  0.158  0.006  0.036  0.060 |
| **Others** | XC_1553  XC_4273  XC_1339(HP)  XC_3464(HP)  XC_1194(HP)  XC_4023(HP)  XC_0282(HP) | | leucin rich protein  leucin rich protein  Putative TPR repeat, SEL1 subfamily(-ve regulator of notch formation)  Putative Hemin uptake protein hemp  Putative von Willebrand factor  Putative SnoaL-like polyketide cyclise  putative methyltransferase | 2.004  1.466  3.374  6.926  0.883  1.030 | 0.033  0.049  0.001  0.0002  0.005  0.018 |
