# Supplementary material for: Co-regulation of Iron Metabolism and Virulence Associated Functions by Iron and XibR, a Novel Iron Binding Transcription Factor, in the Plant Pathogen Xanthomonas
Source: PLoS Pathog. 2016 Nov 30;12(11):e1006019. doi: 10.1371/journal.ppat.1006019 (PMC5130282; doi:10.1371/journal.ppat.1006019)
Supplement: S6 Table — (DOC) [file ppat.1006019.s007.doc]

**Table S6. List of the genes negatively regulated by iron starvation but not influenced by *xibR***.

| **Functional group of genes** | **Locus Tag/gene symbol** | **Product name** | **Microarray**  Fold geomean WT+DP | **P-value** |
| --- | --- | --- | --- | --- |
| **Iron related genes** | XC_3559  XC_2844  XC_3201  XC_0494  XC_1545  XC_1644  XC_0849  XC_2485  XC_0893  XC_1619  XC_0919 | putative siderophore receptor  Bacterioferritin  Bacterioferritin  Bacterioferritin  Oar protein  TonB-dependent receptor  TonB-dependent receptor  TonB-dependent receptor  Oar protein  Oar protein  TonB-dependent receptor | -0.703  -2.073  -2.978  -1.771  -0.722  -1.624  -1.298  -1.475  -1.062  -0.927  -0.765 | 0.015  0.003  0.003  0.011  0.122  0.021  0.024  0.027  0.054  0.132  0.600 |
| **N_2_ Metabolism Related genes** | XC_4114 | nodulation protein | -1.633 | 0.102 |
| **Pathogenicity related genes** | XC_1630 | VirD4 protein | -0.824 | 0.023 |
| **Secretion components**  Type II  Type III  Others | XC_1738  XC_4112(HP) | protein export protein SecD  Putative type VI secretion-associated protein, ImpA family | -0.932  -1.644 | 0.028  0.072 |
| **Flagella biogenesis and regulation** |  |  |  |  |
| **Fimbrial and non fimbrial adhesions**  Nonfimbrial adhesions  Fimbrial adhesions | XC_0940 | fimbrial assembly membrane protein(pilN) | -0.853 | 0.143 |
| **Extracellular Polysaccharides** | XC_1658 | GumB protein | -0.889 | 0.027 |
| **Chemotaxis** | XC_3058 | chemotaxis protein(cheR type) | -0.986 | 0.111 |
| **Two component system** | XC_0495  XC_3057  XC_3406  XC_3956(HP) | Two-component system sensor protein  two-component system sensor protein  two-component system regulatory protein  Putative serine kinase | -1.134  -0.917  -1.408  -2.707 | 0.079  0.088  0.104  0.009 |
| **Transcriptional Regulators** | XC_1020(HP)  XC_0832  XC_4254  XC_4163  XC_0044  XC_0848  XC_3386  XC_2005(HP) | Putative LysR-type transcriptional regulators  transcriptional regulator lysR family  transcriptional regulator for cryptic hemolysin  transcriptional regulator araC family  Transcriptional regulator( TetR family)  transcriptional regulator(AraC)  transcriptional regulator(TetR)  Putative LysR substrate binding domain | -0.849  -1.310  -0.759  -1.925  -1.136  -1.479  -1.127  -0.848 | 0.006  0.008  0.017  0.047  0.240  0.002  0.080  0.023 |
| **Small nucleotide binding proteins** | XC_3157 | hydroxylase molybdopterin-containing subunit | -1.487 | 0.030 |
| **Membrane proteins Transporters and efflux pump** | XC_4316  XC_0502  XC_0640  XC_1995  XC_4064  XC_4248(HP)  XC_3686(HP)  XC_3859  XC_0600  XC_3699  XC_1972  XC_2743(HP)  XC_3788  XC_3990  XC_0672  XC_1434  XC_2928  XC_0674  XC_4068  XC_3771  XC_1279  XC_4166(HP)  XC_1091 | MFS transporter  transmembrane protein  Sulfate permease  ABC transporter ATP-binding protein  Heavy metal transporter  Putative Sulfite exporter TauE/SafE  Putative Outer membrane protein  membrane fusion protein precursor  putative transmembrane protein  outer membrane protein  transport protein  Putative transporter associated domain  ABC transporter ATP-binding protein  transport protein  multidrug resistance efflux pump  outer membrane protein  cation transport protein  MFS transporter  Permease  ATP binding transporter 1  MFS transporter  Putative transmembrane protein of unknown function  outer membrane receptor for transport of vitamin B | -0.811  -0.654  -1.369  -0.711  -1.505  -1.502  -0.781  -1.261  -1.495  -0.639  -1.209  -1.055  -1.341  -1.502  -0.933  -2.420  -1.284  -1.052  -0.754  -1.269  -1.212  -1.167  -0.706 | 0.001  0.012  0.018  0.021  0.028  0.029  0.042  0.046  0.050  0.056  0.059  0.107  0.113  0.116  0.132  0.135  0.143  0.145  0.152  0.160  0.181  0.100  0.036 |
| **Energy and metabolism**  Nucleic acid metabolism and tRNA  Carbohydrate metabolism  Protein/amino acids metabolism    Fatty acid and lipid metabolism  Secondary metabolism | XC_2920(HP)  XC_0884(HP)  XC_3158  XC_1472(HP)  XC_4075  XC_0717(HP)  XC_4135  XC_0798(HP)  XC_3979  XC_1689(HP)  XC_3522(HP)  XC_3791(HP)  XC_4355  XC_4379  XC_3391  XC_0960  (RPH)  XC_4360  XC_0219  XC_3684  XC_3156  XC_4346  XC_2527  (truD)  XC_4388  XC_1684  XC_2964/  rumA  XC_2343  XC_2822  XC_3649  XC_1642  XC_3054  XC_0025(HP)  XC_2138  XC_3032  XC_0452  XC_0112(HP)  XC_1250(HP)  XC_2772  XC_2372(HP)  XC_0446  XC_3742  XC_0018  XC_0411  XC_0654  XC_3592  XC_2833(HP)  XC_3937  XC_3341  XC_3090  (rpsT)  XC_1652  (rpmI)  XC_0800  XC_0592  XC_0511  XC_0255  XC_1394  XC_0799(HP)  XC_0121(HP)  XC_0168(HP)  XC_2649(HP)  XC_2878  XC_0341  XC_4073  XC_0963  XC_0983  XC_4074  XC_0227  XC_1521(HP)  XC_3073  XC_1968  XC_1445  XC_1794  XC_0267  XC_1792  XC_3153  XC_1793 | Putative LigT like Phosphoesterase  Putative Hydrolase/dna  hydroxylase large subunit  Putative S4/Hsp/ tRNA synthetase RNA-binding domain  ribonucleotide-diphosphate reductase beta subunit  PutativeEndonuclease/Exonuclease/phosphatase  Exodeoxyribonuclease III  Putative SAM dependent methyltransferases  mercuric reductase  Putative methyl transferase  Putative methyltransferase  Putative Endoribonuclease L-PSP  tRNA-Arg-CCT  tRNA-Tyr-GTA  exodeoxyribonuclease IX  Ribonuclease PH  tRNA-Ser-TGA  tRNA/rRNA methyltransferase  ATP synthase subunit A  ferredoxin  tRNA-Leu-CAA  tRNA pseudouridine synthase D  tRNA-Thr-TGT  excinuclease ABC subunit C homolog  23S rRNA (uracil-5-)-methyltransferase  putative single stranded DNA exonuclease nucleosidase  Fumarate hydratase  Phosphoglycolate phosphatase  alpha-glucosidase  endo-1,3-beta-glucanase precursor  Putative Peptidase propeptide and YPEB domain (PepSY)  Putative SNARE associated Golgi protein  tryptophan 2,3-dioxygenase  homogentisate 1,2-dioxygenase  putative isoprenylcysteine o-methyltransferase  Putative Peptidase_M23  serine peptidase  Putative Peptidase_C13  tryptophan 2,3-dioxygenase  metallopeptidase(M13 fam)  Putative rhomboid proteases  isopenicillin N epimerase  Prolyl oligopeptidase  ribosomal-protein-alanine acetyltransferase  Putative peptidase  nucleotide sugar epimerase  30S ribosomal protein S10  30S ribosomal protein S20  50S ribosomal protein L35  50S ribosomal protein L31  aspartate aminotransferase  ThiJ/PfpI family protein  CDP-alcohol phosphatidyltransferase  Acyl-CoA carboxyltransferase beta chain  phospholipase A1  Putative GDSL-like Lipase/Acylhydrolase  Putative GDSL-like Lipase / Acylhydrolase  Putative polyhydroxyalkanoic acid system protein (PHA_gran_rgn)  Lipid-A-disaccharide synthase  AttT protein  thioredoxin  coproporphyrinogen iii oxidase  siroheme synthase  flavodoxins  ferredoxin  putative methyltransferase  xenobiotic flavin oxidoreductase a  cytochrome c552  oxydoreductase  Ubiquinol cytochrome C oxidoreductase, cytochrome C1 subunit  nitrile hydratase activator  Ubiquinol-cytochrome C reductase iron-sulfur subunit  tetracenomycin polyketide synthesis protein  ubiquinol cytochrome C oxidoreductase, cytochrome B subunit | -0.633  -0.795  -1.353  -0.647  -0.914  -0.811  -0.772  -1.744  -0.778  -1.571  -1.259  -0.946  -0.702  -0.948  -0.811  -0.675  -0.948  -0.694  -0.737  -1.247  -1.059  -1.305  -1.579  -2.094  -1.186  -0.831  -1.321  -0.829  -1.058  -0.965  -1.769  -1.071  -1.053  -0.923  -0.857  -0.868  -0.973  -1.109  -1.121  -0.988  -1.128  -1.202  -1.422  -0.765  -0.846  -1.140  -1.151  -0.916  -0.906  -0.812  -0.909  -1.318  -0.699  -1.140  -0.752  -1.897  -1.631  -1.090  -0.880  -1.077  -1.144  -0.983  -0.850  -0.750  -1.019  -0.913  -1.002  -1.051  -0.852  -1.174  -0.911  -1.804  -2.104  -1.268  -1.473  -1.679 | 0.002  0.008  0.010  0.020  0.026  0.027  0.044  0.045  0.054  0.055  0.457  0.072  0.076  0.096  0.107  0.116  0.123  0.130  0.179  0.194  0.209  0.101  0.301  0.004  0.030  0.029  0.069  0.003  0.005  0.017  0.025  0.090  0.004  0.033  0.008  0.009  0.030  0.032  0.037  0.041  0.043  0.043  0.052  0.056  0.068  0.088  0.200  0.046  0.054  0.060  0.093  0.017  0.0002  0.026  0.006  0.022  0.006  0.083  0.007  0.047  0.165  0.272  0.005  0.012  0.019  0.047  0.067  0.087  0.089  0.208  0.095  0.167  0.062  0.107  0.023  0.133 |
| **Stress Response** | XC_2930  XC_2897 | heat shock protein  glyoxylase I family protein | -0.651  -0.632 | 0.005  0.020 |
| **Replication and maintenance**  Cell wall biogenesis | XC_2107  XC_2508  (recX)  XC_1053(HP)  XC_3933  XC_0001  (dnaA)  XC_2612  XC_3219(HP)  XC_0355  XC_1906(HP)  XC_3079  XC_0054  XC_2951  XC_1247(HP)  XC_0695  XC_0691  XC_4132  XC_0692  XC_3770(HP)  XC_3820(HP)  XC_3127(HP) | replication initiation protein  RecA regulator RecX  Putative Toxin SymE(SOS)  chromosome partitioning protein  chromosomal replication initiation protein  RadC related protein  Putative HIRAN domain (HIP116, Rad5p N-terminal)  site-specific recombinase  Putative ScpA/B protein ;ScpA and ScpB participate in chromosomal partition during cell division  DNA repair protein  ATP-dependent RNA helicase  DNA ligase  putative AsmA  Rod shape-determining protein (RodA)  Rod shape-determining protein (MreB)  hetI protein  Rod shape-determining protein (MreC)  Putative Glycosyl hydrolase  Uncharacterized protein containing LysM domain  Putative 4-amino-4-deoxy-L-arabinose transferase and related glycosyltransferases of PMT family [Cell envelope biogenesis, outer membrane] | -2.391  -0.853  -1.259  -0.727  -0.923  -1.175  -1.312  -1.839  -0.913  -0.985  -0.821  -1.062  -0.883  -1.224  -0.660  -0.891  -0.688  -0.974  -0.824  -0.852 | 0.128  0.060  0.457  0.028  0.091  0.041  0.075  0.142  0.099  0.011  0.028  0.031  0.101  0.015  0.003  0.102  0.113  0.116  0.131  0.162 |
| **Phage related Proteins** | XC_2773(HP)  XC_2072(HP) | Putative Phage_AlpA  Putative Prophage CP4-57 regulatory protein (AlpA) | -0.997  -1.533 | 0.043  0.122 |
| **Hypothetical Proteins** | XC_0855  XC_4062  XC_2613  XC_1274  XC_1278  XC_2882  XC_1629  XC_0629  XC_2436  XC_4017  XC_2705  XC_4151  XC_0059  XC_4018  XC_0023  XC_3968  XC_3248  XC_0102  XC_3549  XC_2539  XC_3231  XC_1011  XC_1294  XC_1130  XC_1610  XC_2028  XC_1987  XC_0632  XC_0594  XC_1509  XC_0106  XC_2874  XC_1042  XC_3220  XC_0624  XC_1487  XC_0861  XC_2416  XC_2015  XC_3773  XC_3155  XC_3797  XC_2581  XC_2415  XC_0653  XC_2414 | HP  HP  HP  HP  HP  HP  HP  HP  HP  HP  HP  HP  HP  HP  HP  HP  HP  HP  HP  HP  HP  HP  HP  HP  HP  HP  HP  HP  HP  HP  HP  HP  HP  HP  HP  HP  HP  HP  HP  HP  HP  HP  HP  HP  HP  HP | -1.159  -0.789  -1.030  -0.695  -1.098  -0.761  -1.488  -0.944  -0.923  -0.933  -0.749  -0.709  -1.535  -1.200  -0.800  -1.177  -0.988  -1.242  -1.982  -1.302  -0.717  -0.838  -1.800  -1.607  -1.312  -1.048  -0.815  -1.087  -1.160  -0.664  -0.932  -1.452  -1.253  -0.805  -0.811  -0.929  -2.163  -2.519  -0.956  -1.416  -1.545  -0.840  -1.609  -2.024  -0.976  -1.030 | 0.003  0.008  0.011  0.024  0.025  0.026  0.028  0.030  0.033  0.037  0.040  0.044  0.044  0.052  0.053  0.055  0.056  0.061  0.070  0.070  0.077  0.083  0.083  0.092  0.093  0.097  0.098  0.115  0.117  0.126  0.168  0.144  0.152  0.161  0.195  0.197  0.214  0.051  0.057  0.077  0.032  0.164  0.069  0.013  0.021  0.062 |
| **Others** | XC_3447  XC_2646(HP)  XC_2046  XC_3237  XC_1586 | glycine rich protein  Putative Sulfite oxidase  plasmid-related protein  methanol dehydrogenase regulator  dehydrogenase(alcohol/glucoseribitol) | -0.662  -0.677  -1.403  -1.795  -0.966 | 0.002  0.012  0.025  0.064  0.127 |
