# Supplementary material for: Co-regulation of Iron Metabolism and Virulence Associated Functions by Iron and XibR, a Novel Iron Binding Transcription Factor, in the Plant Pathogen Xanthomonas
Source: PLoS Pathog. 2016 Nov 30;12(11):e1006019. doi: 10.1371/journal.ppat.1006019 (PMC5130282; doi:10.1371/journal.ppat.1006019)
Supplement: S7 Table — (DOC) [file ppat.1006019.s008.doc]

**Table S7. List of the genes negatively regulated by iron starvation and positively regulated by *xibR***.

| **Functional group of genes** | **Locus Tag/gene symbol** | **Product name** | **Microarray**  Ratio geomean WT+DP | **P-value** | **Microarray**  Ratio geomean  Mu | **P-value** |
| --- | --- | --- | --- | --- | --- | --- |
| **Iron related genes** | XC_2164(HP)  XC_2190(HP)  XC_3754(HP)  XC_3785  XC_0570  XC_3412(HP) | Putative Ferritin-like, diiron-carboxylate  Putative ferritin related protein  Putative Manganese catalase, ferritin-like diiron-binding domain  ferrichrome-iron receptor 3  iron utilization protein  Putative Secretin and TonB N terminus short domain | -3.327  -1.393  -1.801  -1.390  -1.608  -1.232 | 0.018  0.079  0.073  0.0077  0.073  0.058 | -1.576  -1.474  -2.264  -2.297  -2.237  -3.696 | 0.044  0.111  0.056  0.195  0.121  0.00247 |
| **N_2_ Metabolism Related genes** |  |  |  |  |  |  |
| **Pathogenicity related genes** |  |  |  |  |  |  |
| **Secretion components**  Type II  Type III  Others | XC_1515  XC_3591 | Extracellular protease  pectate lyase | -1.094  -3.627 | 0.00173  0.017 | -2.731  -3.962 | 0.00021  0.0043 |
| **Flagella biogenesis and regulation** |  |  |  |  |  |  |
| **Fimbrial and non fimbrial adhesions**  Nonfimbrial adhesions  Fimbrial adhesions | XC_1627 | fimbrial biogenesis protein | -1.084 | 0.170 | -1.582 | 0.113 |
| **Extracellular Polysaccharides** |  |  |  |  |  |  |
| **Chemotaxis** | XC_1413 | Chemotaxis protein | -2.364 | 0.020 | -2.167 | 0.033 |
| **Two component system** | XC_2886 | sensor protein | -0.974 | 0.187 | -1.635 | 0.078 |
| **Transcriptional Regulators** | XC_0043  XC_0078(HP)  XC_1574  XC_2840  XC_3383 | transcriptional regulator  Putative NmrA  transcriptional regulator (lysR)  MarR family transcriptional regulator  RNA polymerase factor sigma-70 | -0.708  -2.190  -1.188  -1.525  -1.313 | 0.027  0.195  0.110  0.018  0.252 | -1.088  0.301  -1.000  -1.138  -1.247 | 0.031  0.266  0.084  0.100  0.288 |
| **Small nucleotide binding proteins** | XC_0641 | GGDEF family protein | -0.710 | 0.255 | -1.763 | 0.087 |
| **Membrane proteins Transporters and efflux pump** | XC_0881  XC_0332(HP)  XC_2450  XC_2837  XC_2838  XC_2839  XC_3731 | Ion transporter  Putative Phosphate-selective porin O and P  integral membrane protein  multidrug resistance membrane translocase  multidrug resistance efflux pump  outer membrane efflux protein  ABC transporter ATP-binding protein | -1.213  -1.368  -0.780  -1.205  -1.220  -1.224  -2.455 | 0.227  0.034  0.338  0.120  0.038  0.036  0.141 | -3.696  -1.723  -1.187  -1.353  -0.999  -1.039  -2.584 | 0.069  0.016  0.195  0.092  0.041  0.041  0.133 |
| **Energy and metabolism**  Nucleic acid metabolism and tRNA  Carbohydrate metabolism  Protein/amino acids metabolism  Fatty acid and lipid metabolism  Secondary metabolism | XC_0908  XC_1385  XC_1386  XC_1387  XC_1388(HP)  XC_4190(HP)  XC_4373  XC_4387  XC_0777  XC_1166  XC_1648  XC_2169  XC_3028  XC_1512  XC_4322(HP)  XC_2191  XC_2585  XC_2586(HP)  XC_2468  XC_2511  XC_3073 | endonuclease  oxidoreductase(purine metabolism)  Oxidoreductase(nucleotide degradation)  Oxidoreductase(nucleotide degradation)  Putative xanthine dehydrogenase accessory protein XdhC  Putative tRNA 2-thiocytidine biosynthesis protein TtcA  His tRNA  Pseudo tRNA  Esterase  glucokinase  Cyclomaltodextrin glucanotransferase  glucose-1-phosphate cytidylyltransferase  transminase  PmbA protein  Putative rhomboid proteases  fatty acid alpha hydroxylase  Dehydrogenase(acyl-CoA dehydrogenase)  Putative GlcNAc-PI de-N-acetylase  3-methyl-2-oxobutanoate hydroxymethyltransferase  ubiquinone biosynthesis protein  xenobiotic flavin oxidoreductase A | -1.000  -0.939  -1.606  -1.098  -1.813  -1.325  -0.816  -0.975  -1.265  -2.583  -1.397  -3.536  -2.597  -1.307  -0.948  -1.335  -0.891  -1.563  -0.836  -0.934  -0.852 | 0.209  0.081  0.0091  0.0062  1.02E-4  0.020  0.088  0.029  0.436  0.022  0.053  0.109  0.104  0.040  0.174  0.105  0.109  0.118  0.014  0.160  0.089 | -0.838  -1.184  -1.387  -1.643  -0.901  -0.965  -1.138  -1.055  -2.081  -1.439  -0.967  -3.699  -2.378  -1.308  -1.178  -3.559  -1.323  -1.322  -0.636  -1.478  -1.454 | 0.280  0.013  0.0066  0.0056  0.024  0.091  0.0599  0.035  0.251  0.064  0.030  0.102  0.140  0.027  0.137  0.024  0.083  0.200  0.0039  0.120  0.038 |
| **Stress Response** | XC_1687(HP)  XC_3548 | putative Stress-induced protein  arsenate reductase | -0.973  -1.194 | 0.111  0.048 | -1.963  -1.418 | 0.020  0.0057 |
| **Replication and maintenance** | XC_1041(HP)  XC_2612 | Putative integrating conjugative element protein  RadC family protein | -3.737  -1.175 | 0.239  0.041 | -3.415  -1.343 | 0.270  8.80E-04 |
| **Cell wall biogenesis** | XC_2588  XC_3768(HP)  XC_2168(HP) | glycosyl transferase-related protein  Putative Glycosyltransferases  Putative Glycosyltransferases | -1.243  -0.993  -1.895 | 0.030  0.073  0.054 | -1.714  -1.598  -1.289 | 0.019  0.027  0.109 |
| **Phage related Proteins** |  |  |  |  |  |  |
| **Hypothetical Proteins** | XC_0073  XC_0091  XC_0102  XC_0140  XC_0521  XC_0566  XC_0612  XC_1297  XC_1392  XC_1425  XC_2185  XC_2788  XC_2904  XC_2922  XC_3178  XC_3729  XC_3764  XC_3772  XC_3775  XC_4201 | HP  HP  HP  HP  HP  HP  HP  HP  HP  HP  HP HP  HP  HP  HP  HP  HP  HP  HP  HP | -1.320  -2.406  -1.242  -1.293  -0.973  -2.426  -0.873  -1.274  -0.848  -0.854  -1.320  -1.701  -1.137  -0.973  -1.200  -0.745  -0.713  -1.630  -0.921  -1.021 | 0.134  0.035  0.061  0.115  0.030  0.169  0.028  0.0087  0.060  0.064  0.069  0.350  0.040  0.092  0.222  0.018  0.023  0.076  0.147  0.069 | -1.392  -2.667  -1.039  -1.508  -1.367  -2.779  -0.708  -1.639  -1.490  -0.935  -4.134  -2.928  -1.086  -1.120  -0.731  -0.714  -0.986  -1.232  -1.082  -1.642 | 0.050  0.016  0.067  0.016  0.054  0.136  0.032  0.012  0.042  0.116  0.008  0.150  0.148  0.093  0.376  0.019  0.029  0.106  0.119  0.034 |
| **Others** | XC_2503(HP)  XC_3167 | Putative Pirin-related protein  short chain dehydrogenase | -0.821  -1.017 | 0.412  0.144 | -1.917  -1.369 | 0.138  0.110 |
