# Supplementary material for: Co-regulation of Iron Metabolism and Virulence Associated Functions by Iron and XibR, a Novel Iron Binding Transcription Factor, in the Plant Pathogen Xanthomonas
Source: PLoS Pathog. 2016 Nov 30;12(11):e1006019. doi: 10.1371/journal.ppat.1006019 (PMC5130282; doi:10.1371/journal.ppat.1006019)
Supplement: S8 Table — (DOC) [file ppat.1006019.s009.doc]

**Table S8. List of the genes positively regulated by iron starvation and negatively regulated by *xibR*.**

| **Functional group of genes** | **Locus Tag/gene symbol** | **Product name** | **Microarray**  Ratio geomean WT+DP | **P-value** | **Microarray**  Ratio geomean  Mu | **P-value** |
| --- | --- | --- | --- | --- | --- | --- |
| **Iron related genes** | XC_0558  XC_0261(HP) | ferric pseudobactin M114 receptor protein  Putative iron-regulated membrane protein | 2.462  3.545 | 0.262  0.008 | 4.262  0.768 | 0.017  0.095 |
| **N_2_ Metabolism Related genes** | XC_0042 | NonF-related protein | 1.077 | 0.069 | 3.921 | 0.014 |
| **Pathogenicity related genes** | XC_0052 | avirulence protein | 2.019 | 0.013 | 1.719 | 0.0029 |
| **Secretion components**  Type II  Type III  Others | XC_3022 | HpaB protein | 1.875 | 0.035 | 0.727 | 0.0013 |
| **Flagella biogenesis and regulation** |  |  |  |  |  |  |
| **Fimbrial and non fimbrial adhesions** |  |  |  |  |  |  |
| **Extracellular Polysaccharides** |  |  |  |  |  |  |
| **Chemotaxis** |  |  |  |  |  |  |
| **Two component system** |  |  |  |  |  |  |
| **Transcriptional Regulators** |  |  |  |  |  |  |
| **Small nucleotide binding proteins** |  |  |  |  |  |  |
| **Membrane proteins Transporters and efflux pump** |  |  |  |  |  |  |
| **Energy and metabolism**  Protein/amino acids metabolism  Secondary metabolism | XC_2981  XC_0686  XC_2924 | methylmalonate-semialdehyde dehydrogenase  alcohol dehydrogenase class III  GTN reductase | 1.015  0.942  0.856 | 0.020  0.161  0.019 | 0.848  0.006  0.680 | 0.011  4.950  0.012 |
| **Stress Response** |  |  |  |  |  |  |
| **Replication and maintenance** |  |  |  |  |  |  |
| **Cell wall biogenesis** | XC_0125 | pectin methylesterase-like protein | 2.466 | 0.112 | 3.734 | 0.00968 |
| **Phage related Proteins** |  |  |  |  |  |  |
| **Hypothetical Proteins** | XC_0263  XC_0610 | HP  HP | 4.351  1.222 | 0.0062  0.104 | 0.635  0.674 | 0.0031  0.145 |
| **Others** | XC_3426 | protocatechuate degradation protein | 1.894 | 0.0005 | 1.040 | 0.031 |
