# Supplementary material for: Co-regulation of Iron Metabolism and Virulence Associated Functions by Iron and XibR, a Novel Iron Binding Transcription Factor, in the Plant Pathogen Xanthomonas
Source: PLoS Pathog. 2016 Nov 30;12(11):e1006019. doi: 10.1371/journal.ppat.1006019 (PMC5130282; doi:10.1371/journal.ppat.1006019)
Supplement: S9 Table — (DOC) [file ppat.1006019.s010.doc]

Table S9. List of the genes positively regulated by both iron starvation and XibR.

| **Functional group of genes** | **Locus Tag/gene symbol** | **Product name** | **Microarray**  Ratio geomean WT+DP | **P-value** | **Microarray**  Ratio geomean  Mu | **P-value** |
| --- | --- | --- | --- | --- | --- | --- |
| **Iron related genes** | XC_0924  XC_3462(HP)  XC_2512 | outer membrane receptor for ferric iron uptake  Putative Haem utilisation ChuX/HutX  TonB-dependent receptor | 1.233  3.906  1.694 | 0.064  0.033  0.03 | -1.608  -2.059  -2.012 | 0.039  0.087  0.046 |
| **N_2_ Metabolism Related genes** | XC_3401(HP) | Putative type 1 glutamine amidotransferase (GATase1) like protein | 0.687 | 0.056 | -0.793 | 0.084 |
| **Pathogenicity related genes** | XC_2087  XC_0431 | Tannase precursor  VirK protein | 1.471  1.088 | 0.0051  0.018 | -1.982  -1.095 | 0.0046  0.011 |
| **Secretion components**  Type II  Type III  Others | XC_0784  XC_0026  XC_3373  XC_3012 | Cellulase S  Cellulase  Xylanase  Type III secretion system protein HrcU | 1.618  1.106  0.910  2.337 | 0.051  0.00496  0.119  0.041 | -1.032  -4.293  -1.369  -1.064 | 0.023  0.00271  0.018  0.073 |
| **Flagella biogenesis and regulation** | XC_1337(HP)  XC_2237/flgE  XC_2280  XC_2263  XC_2242/flgJ  XC_2262  XC_2236/flgD    XC_2238/flgF  XC_2297/motC  XC_2279  XC_2243/flgK    XC_2244/flgL  XC_2241/flgI  XC_2264  XC_2247  XC_2245  XC_2235/flgC  XC_2261  XC_2269  XC_2270/flip  XC_2298  XC_2230(HP)  XC_2232/flgA | Putative flagellar hook capping protein FlgD  Flagellar hook protein FlgE  Flagellar biosynthesis switch protein  Flagellar protein  Flagellar rod assembly protein/muramidase FlgJ  Flagellar protein  Flagellar basal body rod modification protein  Flagellar basal body rod protein FlgF  flagellar motor protein MotC  flagellar biosynthesis regulator FlhF  flagellar hook-associated protein FlgK  lagellar hook-associated protein FlgL  Flagellar basal body P-ring protein  Flagellar FliJ protein  Flagellar protein  Flagellin  Flagellar basal body rod protein FlgC  Flagellar protein  Flagellar protein  Flagellar biosynthesis protein FliP  Flagellar motor protein MotD  Putative FlgN protein  Flagellar basal body P-ring biosynthesis protein FlgA | 3.320  1.683  1.547  1.344  1.305  1.225  1.220  1.218  1.169  1.129  1.114  1.092  1.066  0.988  0.960  0.938  0.870  0.827  0.809  0.745  0.743  0.733  0.716 | 0.00096  0.00819  0.00061  0.024  0.041  0.017  0.012  0.042  0.057  0.029  0.045  0.038  0.00728  0.00513  0.03962  0.0097  0.026  0.031  0.016  0.00476  0.102  0.078  0.00795 | -1.200  -3.828  -3.643  -5.054  -3.184  -1.430  -3.556  -2.835  -1.510  -2.766  -2.426  -4.836  -1.369  -4.158  -3.458  -4.680  -4.158  -4.680  -3.556  0.7458  -3.265  -3.058  -5.380 | 0.015  0.00266  0.0054  0.00419  0.219  0.017  8.20E-04  0.025  0.039  0.00582  0.039  0.00481  0.073  9.22E-05  0.09981  0.00352  0.0104  0.00426  0.159  0.00476  0.00704  0.00823  0.00173 |
| **Fimbrial and non fimbrial adhesions**  Nonfimbrial adhesions  Fimbrial adhesions | XC_2858  XC_2857 | Pili assembly chaperone  Protein U | 1.319  0.852 | 0.0481  0.082 | -3.988  -3.838 | 0.00815  0.00174 |
| **Extracellular Polysaccharides** | XC_1668  XC_1669 | GumL protein  GumM protein | 1.111  0.756 | 0.068  0.013 | -1.971  -1.490 | 0.00943  0.00402 |
| **Chemotaxis** | XC_2300  XC_2283  XC_2303  XC_2282  XC_0638 | chemotaxis protein  chemotaxis related protein  chemotaxis protein, CheA1  chemotaxis protein  chemotaxis protein | 1.329  1.326  1.027  1.000  0.7111 | 0.0298  0.00072  0.019  0.00951  0.023 | -3.411  -4.756  -0.713  -4.107  -2.465 | 0.00637  1.50-E4  0.026  0.00122  0.0098 |
| **Two component system** | XC_3160  XC_2475 | Transducer protein car  Sensor kinase | | 2.208  1.061 | | --- | | 0.0408  0.092 | | -1.089  -0.664 | | --- | | 0.092  0.071 |
| **Transcriptional Regulators** | XC_2281 | RNA polymerase sigma factor | 1.224 | 0.017 | -2.805 | 0.00808 |
| **Small nucleotide binding proteins** |  |  |  |  |  |  |
| **Membrane Transporters and efflux pump** | XC_2476 | MFS transporter | 1.303 | 0.016 | -1.771 | 0.01006 |
| **Energy and metabolism**  Nucleic acid metabolism and tRNA  Carbohydrate metabolism  Protein/amino acids metabolism  Fatty acid and lipid metabolism  Coenzymes/Secondary metabolism | XC_3177(HP)  XC_0705  XC_0356  XC_2478  XC_4191  XC_0279/dkgB  XC_3159  XC_1218  XC_0419(HP)  XC_1422  XC_2088(HP)  XC_0281  XC_0280  XC_0154 | Putative Inosine-uridine preferring nucleoside hydrolase  endopolygalacturonase  dihydroxy-acid dehydratase  D-xylulokinase  xylose isomerase  2,5-diketo-D-gluconate reductase B  periplasmic beta-glucosidase  beta-mannosidase  putative carbohydrate hydrolase  Cysteine protease  Putative avidin  oxidoreductase  Quinone oxidoreductase  2-keto-4-pentenoate hydratase | 2.085        1.927  1.4906  1.4388  1.3763  1.2928    0.9986  0.7519   1.515    1.8127              1.3329  1.3004  1.2203   0.7415 | 0.0046  0.017  0.083  0.00976  0.019  0.044  0.018  0.064  0.00122  0.00564  0.0297  0.017  0.081  0.108 | -2.910  -0.859  -0.935  -1.527  -1.599  -1.795  -2.120  -3.158  -0.694  -2.177  -3.442  -1.403  -1.771  -1.204 | 0.00576  0.0399  0.0499  0.064  0.042  0.00026  0.012  0.00439  0.00263  0.013  0.00148  0.011  0.017  0.016 |
| **Stress Response** |  |  |  |  |  |  |
| **Replication and maintenance** |  |  |  |  |  |  |
| **Cell wall biogenesis** | XC_3562(HP) | Putative glycosyl transferase | 1.062 | 0.074 | -1.666 | 0.00517 |
| **Phage related Proteins** |  |  |  |  |  |  |
| **Hypothetical Proteins** | XC_0817  XC_3176  XC_3922  XC_2789  XC_2474  XC_2830  XC_4206  XC_0147  XC_2248 | HP  HP  HP  HP  HP  HP  HP  HP  HP | | 2.956  2.344  2.165  1.362  1.299  1.272  1.113  0.835  0.818 | | --- | | 0.0033  0.0103  0.018  0.128  0.085  8.80-E4  0.037  0.117  0.00804 | -1.358  -1.002  -3.171  -2.217  -1.937  -2.836  -0.766  -1.522  -1.776 | 0.0092  0.00153  0.00334  0.053  0.013  0.00027  0.018  0.059  0.00710 |
| **Others** | XC_2438  XC_1338(HP)  XC_0936 | Plasmid-related protein  Putative PepSY-associated TM helix  Methanol dehydrogenase regulatory protein | 1.400  0.851  0.793 | 0.097  0.155  0.055 | -1.207  -2.336  -0.865 | 0.128  0.103  0.042 |
