# Supplementary material for: Co-regulation of Iron Metabolism and Virulence Associated Functions by Iron and XibR, a Novel Iron Binding Transcription Factor, in the Plant Pathogen Xanthomonas
Source: PLoS Pathog. 2016 Nov 30;12(11):e1006019. doi: 10.1371/journal.ppat.1006019 (PMC5130282; doi:10.1371/journal.ppat.1006019)
Supplement: S10 Table — (DOC) [file ppat.1006019.s011.doc]

**Table S10.** List of the genes negatively regulated by both iron starvation and *xibR*.

| **Functional group of genes** | **Locus Tag/gene symbol** | **Product name** | **Microarray**  Ratio geomean WT+DP | **P-value** | **Microarray**  Ratio geomean  Mu | **P-value** |
| --- | --- | --- | --- | --- | --- | --- |
| **Iron related genes** |  |  |  |  |  |  |
| **N_2_ Metabolism Related genes** |  |  |  |  |  |  |
| **Pathogenicity related genes** |  |  |  |  |  |  |
| **Secretion components**  Type II  Others | XC_3305(HP)  XC_2413 | Putative Cell Wall Hydrolase  Putative NTPase VagA (Type IV secretory system Conjugative DNA transfer) | -1.092  -1.044 | 0.133  0.113 | 0.624  0.712 | 0.009  0.009 |
| **Flagella biogenesis and regulation** |  |  |  |  |  |  |
| **Fimbrial and non fimbrial adhesions** |  |  |  |  |  |  |
| **Extracellular Polysaccharides** |  |  |  |  |  |  |
| **Chemotaxis** |  |  |  |  |  |  |
| **Two component system** |  |  |  |  |  |  |
| **Transcriptional Regulators** |  |  |  |  |  |  |
| **Small nucleotide binding proteins** |  |  |  |  |  |  |
| **Membrane Transporters and efflux pump** | XC_0174 | ABC transporter amino acid permease | -0.941 | 0.033 | 1.118 | 0.00505 |
| **Energy and metabolism**  Carbohydrate metabolism  Protein/amino acids metabolism | XC_0761  XC_1789  XC_3837  XC_2953(HP) | betaine aldehyde dehydrogenase  glutathione S-transferase (Posttranslational modification)  glutathione S-transferase  Putative metallophosphatase | -0.821  -1.626  -1.377  -1.062 | 0.025  0.027  0.0078  0.152 | 2.796  0.96  0.987  0.989 | 0.00065  0.031  0.054  0.0398 |
| **Stress Response** |  |  |  |  |  |  |
| **Replication and maintenance** |  |  |  |  |  |  |
| **Cell wall biogenesis** |  |  |  |  |  |  |
| **Phage Related genes** |  |  |  |  |  |  |
| **Hypothetical Proteins** | XC_3556  XC_3553 | HP  HP | 0.459  0.528 | 0.182  0.132 | 3.019  2.788 | 0.009  0.009 |
| **Others** | XC_3839(HP)  XC_3034 | Putative Lab A like protein  (role in circadian rhythm)  IS1595 transposase | 0.599  -0.685 | 0.039  0.027 | 0.681  0.807 | 0.038  0.015 |
