# Supplementary material for: Co-regulation of Iron Metabolism and Virulence Associated Functions by Iron and XibR, a Novel Iron Binding Transcription Factor, in the Plant Pathogen Xanthomonas
Source: PLoS Pathog. 2016 Nov 30;12(11):e1006019. doi: 10.1371/journal.ppat.1006019 (PMC5130282; doi:10.1371/journal.ppat.1006019)
Supplement: S11 Table — (DOC) [file ppat.1006019.s012.doc]

**Table S11.** Generations time of Xcc strains.

|  | **Generation time (h)** | | |
| --- | --- | --- | --- |
|  | **PS** | **PS+DP** | **PS+DP+FeSO_4_** |
| **Xcc 8004** | 2.26 ± 0.08 | 3.98 ± 0.13 | 2.36 ± 0.05 |
| **Δ*xibR*** | 2.21 ± 0.05 | 4.86 ± 0.33* | 2.39 ± 0.02 |
| **Δ*xibR*/pSSP30** | 2.36 ± 0.05 | 4.11 ± 0.24 | 2.37 ± 0.02 |
| **Δ*xibR*/pSSP39** | 2.38 ± 0.04 | 5.1 ± 0.15** | 2.37 ± 0.03 |
| **Δ*fhuE* ΔXC_0925** | 2.32 ± 0.13 | 4.09 ± 0.1 | 2.35 ± 0.03 |
| **Δ*fecR*** | 2.35 ± 0.04 | 4.89 ± 0.46* | 2.4 ± 0.04 |
| **Δ*yciE* Δ*yciF* ΔXC_3754** | 2.37 ± 0.05 | 5.21 ± 0.37** | 2.39 ± 0.05 |

Generation time values are mean of three biological replicates ± SD. * indicates P<0.05 and ** indicates P<0.01 while comparing with wild type in same medium by paired student T-Test.
