# Supplementary material for: Co-regulation of Iron Metabolism and Virulence Associated Functions by Iron and XibR, a Novel Iron Binding Transcription Factor, in the Plant Pathogen Xanthomonas
Source: PLoS Pathog. 2016 Nov 30;12(11):e1006019. doi: 10.1371/journal.ppat.1006019 (PMC5130282; doi:10.1371/journal.ppat.1006019)
Supplement: S13 Table — (DOC) [file ppat.1006019.s014.doc]

**Table S13. The primers used in this study.**

| **Primers name** | **Nucleotide sequences of primers** |
| --- | --- |
| SC Intsx1  SC Clamp  SC tnpRL17-1  SC tnpRL13-2  SC Extdx  SC Extsx  SC Ext-1  SC ARB-1A  SC ARB-1B  SC ARB1  SC ARB2  SC ARB8  SC60 F  SC62R  SC63F  SC69F  SC70R  SC71F  SC73R  SC74F  SC75F  SCP3F  SCP4R  SCP5F  SCP6R  SCP18R  SCP19R  SCP20F  SCP21R  SCP22F  SCP23R  SCP28R  SCP29F  SCP32F  SCP33R  Sidxcdel1F  Sidxcdel1R  Sidxcdel2F  Sidxcdel2R  SCP52F  SCP52R  SCP59F  SCP60R  SCP61F  SCP62R  SCP64F  SCP65R  SCP66F  SCP67R  SCP69F  SCP70R  SCP71F  SCP72R  SCP73R  SCP74F  SCP75R  SCP76F  SCP77R  SCP_flg1 chip F  SCP_flg1 chip R  SCP_mot Chip F  SCP_mot Chip R  SCP_xsuA Chip F  SCP_xsuA Chip R  SCP_motEMSA F  SCP_motEMSAR  SCP_xsuEMSA F  SCP_xsuEMSAR  SCP_flg1_prom F  SCP_flg1_prom R1 SCP_flg1_prom R2  SCP_mot_prom F  SCP_mot_prom R1  SCP_mot_prom R2  SCP_sid_prom F  SCP_sid_prom R1  SCP_sid_prom R2  SCP_xibR_prom F  SCP_xibR_prom R  RT 1 F (*feoA*)  RT 1 R (*feoA*)  RT 2 F (*XC_1108*)  RT 2 R (*XC_1108*)  RT 4 F (*XC_3001*)  RT 4 R (*XC_3001*)  RT 6 F (*XC_1414*)  RT 6 R (*XC_1414*)  RT 7 F (*XC_0027*)  RT 7 F (*XC_0027*)  RT 9 F (*XC_3012*)  RT 9 R (*XC_3012*)  RT 12 F (*XC_1337*)  RT 12 R (*XC_1337*)  RT 13F (*XC_2300*)  RT 13 R (*XC_2300*)  RT 15 F (*XC_3591*)  RT 15 R (*XC_3591*)  RT 16 F (*gumL*)  RT 16 R (*gumL*)  RT 17 F (*atsE*)  RT 17 R (*atsE*)  RT 18 F (*XC_2245*)  RT 18 R (*XC_2245*)  RT 19 F (*XC_2230*)  RT 19 R (*XC_2230*)  RT 20 F (*XC_2247*)  RT 20 R (*XC_2247*)  RT 28 F (*XC_1035*)  RT 28 R (*XC_1035*)  RT 30 F (*XC_3006*)  RT 30 R (*XC_3006*)  RT 33F (*hrcV*)  RT 33R (*hrcV*)  RT 38 F (*XC_2602*)  RT 38 R (*XC_2602*)  RT 39 F (*feoB*)  RT 39 R (*feoB*)  RT 40 F (*fur*)  RT 40 R (*fur*)  RT 41 F (*xssA*)  RT 41 R (*xssA*)  RT 42 F (*xibR*)  RT 42 R (*xibR*)  RT 43 F (*XC_3752*)  RT 43 R (*XC_3752*)  RT 44 F (*XC_0557*)  RT 44 R (*XC_0557*)  RT 45 F (*XC_0925*)  RT 45 R (*XC_0925*)  RT 49 F (*XC_2858*)  RT 49 R (*XC_2858*)  RT 50 F (*XC_2857*)  RT 50 R (*XC_2857*)  RT 54 F (*XC_2164*)  RT 54 R (*XC_2164*)  RT 55 F (*XC_2190*)  RT 55 R (*XC_2190*)  XCHrpG RT F  XCHrpG RT R  XCHrpX RT F  XCHrpX RT R  D55AXibR F  D55AXibR R  N2Srec F  N2Srec R  N2Ssigma F  N2Ssigma R  N2Sdnab F  N2Sdnab R  S2Nrec F  S2Nrec R  S2Nsigma F  S2Nsigma R  S2Ndnab F  S2Ndnab R | 5’-CGCACTGAGAAGCCCTTAGAGC-3’  5’-GACCACGAGACGCCACACT-3’  5’-AACAAGCCAGGGATGTAACG-3’  5’-CAGCAACACCTTCTTCACGA-3’  5’-CCAGAAAGTGAGGGAGCCA-3’  5’-GACAACAAGCCAGGGATG-3’  5’-CGAACTAAACCCTCATGGCTAACG-3’  5’-GGCCACGCGTCGACTAGTAC(AGCT)(AGCT)(AGCT)(AGCT)(AGCT)(AGCT) (AGCT)(AGCT)(AGCT)(AGCT)GATAT-3’  5’-GGCCACGCGTCGACTAGTAC(AGCT)(AGCT)(AGCT)(AGCT)(AGCT)(AGCT) (AGCT)(AGCT)(AGCT)(AGCT)ACGCC-3’  5’-GACCACGAGACGCCACACT(AGCT)(AGCT)(AGCT)(AGCT)(AGCT)(AGCT) (AGCT)(AGCT)(AGCT)(AGCT)CATG-3’  5’-GACCACGAGACGCCACACT(AGCT)(AGCT)(AGCT)(AGCT)(AGCT)(AGCT)(AGCT)(AGCT)(AGCT)(AGCT)(AGCT)(AGCT)CAGCG-3’  5’-GACCACGAGACGCCACACT(AGCT)(AGCT)(AGCT)(AGCT)(AGCT)(AGCT) (AGCT)(AGCT)(AGCT)(AGCT)GACGT-3’  5’-GCAAGCTTAGCACGACGCAGATGGCGCAGGCAGCGCG-3’  5’-GCGAATTCTCACGCGTTGTCCTTGTCGCTCAGGC-3’  5’-GCAAGCTTAGGAGGACAGCTTTGCGGACAGAAGCGCCTATG-3’  5’-GCGAATTCTTCGGTGTCAGCGAGTTCCTCACCA-3’  5’-GCAAGCTTGGGCGGCACCTGGATCGGGTATTCGTTGAG-3’  5’-GCAAGCTTAGGAGGACAGCTATGGCTGAGGCGGCCGCCGTATC-3’  5’-GCGAGCTCTCAGCGCCGCTTGCGCCCCGGCCCCAGC-3’  5’-GCAAGCTTAGGAGGACAGCTATGCACCATCATCATCATCATATGGCTGAGGCCGCCGCCGTATC-3’  5’-GCAAGCTTAGGAGGACAGCTATGTACCCATACGATGTTCCAGATTACGCTATGGCTGAGGCCGCCGCCGTATC-3’  5’-GCCGGATCCCGGACAGAAGCGCCTATGGAC-3’  5’-GCGTCTAGACGCCTCACGCAGCCCGCC-3’  5’-GCGTCTAGAGATCACGCGCAGGCCGAG-3’  5’-GCAAGCTTTCACGCGTTGTCCTTGTC-3’  5’-GCGAATTCTCAATGATGATGATGATGGTGCGCGTTGTCCTTGTCGCTCAGGC-3’  5’-GCGAATTCTCAAGCGTAATCTGGAACATCGTATGGGTACGCGTTGTCCTTGTCGCTCAGGC-3’  5’-GCGGATCCGTATCCCACCGCATCTGG-3’  5’-GCTCTAGAGGGCAGTGCACGCGCGGC-3’  5’-GCTCTAGA ATGCTACCCAAGCGCTTGTCGTCT-3’  5’-GCAAGCTTCAGCTTGCGGGTCACGGTGTT-3’  5’-CTTCTTCGCCCAGCTGATT-3’  5’-TCGAACGGGTAATAGCCAAG-3’  5’-ACGGCACGCTGACCTATC-3’  5’-GGCAGTGTACGGGTGACG-3’  5’-GCGGATCCTCCTGTCGAACGAGAGCT-3’  5’-GCTCTAGAGTGATCGCTGTTGCTGAA-3’  5’-GCTCTAGAATCAACTACCGCAGCATC-3’  5’-GCAAGCTTCAGCTCCGGTGGGTCGAT-3’  5’-GGGCGCCTACCTCATTGAT-3’  5’-GTGCCAGGCCGCGATGT-3’  5’-GCTCTAGAGGTAGGGAATTGGACTCCAAACTGAG-3’  5’-GCGGATCCGGTGATGCTCTGCAGGTTCTGGTCGT-3’  5’-GCGGATCCGTCAAGGCTGCCTGGTTCGACGACAAG-3’  5’-GCAAGCTTGTCGTCGCAGTCGCAAGAGAACGATCA-3’  5’-GCGGATCCCAGGACACCTTCATCAGCGTGATCGA-3’  5’-GCTCTAGACCAGTCACGTAATTGCGCCGCGCT-3'  5’-GCTCTAGACAGCGCAGCCGCTACTGGGTGAGCAT-3’  5’-GCCAAGCTTGAGAGGTGCAGCGAAACAGCGTGGT-3’  5’-GCTCTAGAGCTCCATCGCATTGCGCGGACTGAACA-3’  5’-CGGAATTCCACGCCCTTGGTGACCTCATCGTTCA-3’  5’-CGGAATTCCACCTGGAGATCATCGGCTCGATCATTG-3’  5’-GCAAGCTTGTTGGAGAGCAGGCGCAGCGACATCG-3’  5’-GGTCTGCGCCAGTGACGCGGTCCAGCT-3’  5’-GAGGCGCTACTCAAAATTGG-3’  5’-GCAGAATCTCAACGCAAACA-3’  5’-CCTGTACACCGACCACCAC-3’  5’-GCACTGACACCGTCATCAAG-3’  5’-CTATCAACAAAGCCGCCAGT-3’  5’-ATCAAGAGCTTGGGTGGTTG-3’  5’-TCACCGAAACCTACAATCGAC-3’  5’-TGAACTCAGTGGTGCCTCAG-3’  5’-CATATGTTCCAGGTCCAGCA-3’  5’-GACTCGCGATCCAGAAAAAC-3’  5’-GCAACCAGGCGCAAGCGGCTCA-3’  5’-CCAGTCCAATGAGGCTGAGTCTGTCCA-3’  5’-CGACAACACATCGCCTTTCAA-3’  5’-CGATCAGTTTCTCGTCCACCT-3’  5’-GCAAGCTTGCCAACCACTTCGAGAGCAGGCGC-3’  5’-GCTCTAGACGTCTGCTGCGCATCCAGTCCGGT-3’  5’-GCGAATTCCGTCTGCTGCGCATCCAGTCCGGT-3’  5’-GCAAGCTTGTATCCATCCAACGACCGGACA-3’  5’-CGTCTAGACACCAGTCCAATGAGGCTGAGT-3’  5’-GCGAATTCCACCAGTCCAATGAGGCTGAGT-3’  5’-GCAAGCTTCGACAACACATCGCCTTTCAA-3’  5’-GCTCTAGAGTGGAAACGGAACGTCGGCAT-3’  5’-GCGAATTCGTGGAAACGGAACGTCGGCAT-3’  5’-GCAAGCTTCACCTGTGTCGCCTCAGTGTTG-3’  5’-GCTCTAGAGAACTCCACGTCGTCGTCGATGA-3’  5’-ACGTTGTCGGATATGCCATT-3’  5’-GCGTATAGCCAACCTGCAC-3’  5’-ATTTTCTGGTCGGCAATCTG-3’  5’-TCTGGTTGAGCGTACTCACG-3’  5’-GATCCATCTGCGACGTCTTT-3’  5’-CCTGATCAGAATCGCATGAA-3’  5’-GAAGAGGTACGCGAAAGTGC-3’  5’-CTTTTCCACCACCGACTTGT-3’  5’-TCGACTACCGCTATCCAACC-3’  5’-GGCCGTAGTACTTGGCGTAG-3’  5’-GCCGACAGAAAAGAAACTGC-3’  5’-GTCAGCAAGACACCCTGGAT-3’  5’-AGCTCAATGTCGCCGAATAC-3’  5’-TTGGTATCGGTGAACGACAA-3’  5’-CTGCTGGCAGAACTGGATTC-3’  5’-GTTCCAGCGCATAGGTTTGT-3’  5’-GGCAACTGGAACTCCGAATA-3’  5’-TTCTCGTGCAGCTTGAACAC-3’  5’-AACGAGCCGGTTGAGAAGTA-3’  5’-CCTGGTGCCGTGGTAGTAGT-3’  5’-ACTGGACGAAATGACCTTCG-3’  5’-ATATCTTCCAGCGGCAGGTT-3’  5’-CAGCGTAACCTCAACACCAA-3’  5’-GCCAGTGAGATACCGTCGTT-3’  5’-CATTGATCGAGCACGACATC-3’  5’-CAGTGCCCAGTTCACCTCAC-3’  5’-AGTTGGTGTCGCTGCTGTTT-3’  5’-CGCTGGATCACGTAGTCGTA-3’  5’-ATATCGGCAAGATCGCTGTC-3’  5’-CGTAAAGCAACTGGGACCAT-3’  5’-GATGTCAACGTGATCGTGCT-3’  5’-CGAGTCCATCATCAGCAGAA-3’  5’-CGAAGTGTTGATCCACGATG-3’  5’-TTGTTGACCTCTGCAACCAG-3’  5’-CACATCTACGCGCTTTACCA-3’  5’-GTCAGGCTTGTCGAATCGTT-3’  5’-TCGTGGACGATGAAGAAGTG-3’  5’-CAGCAGGAACCACAACAGAA-3’  5’-TCGTGGACGATGAAGAAGTG-3’  5’-CAGCAGGAACCACAACAGAA-3’  5’-ATGCCATAGCGAATTCATCC-3’  5’-ATGTTCGGCCACGTAATAGC-3’  5’-ATTGGCTGGACAGCAACTTC-3’  5’-ACTCGCTGACACCGAACTG-3’  5’-GAGCACTACCCCGAACTCAA-3’  5’-GGTAGGACGCAATTTCCAGA-3’  5’-CAGCAATGCGTGTTCAATG-3’  5’-GTATCGTCCAGCGGAAACAC-3’  5’-ATCACCGATGTGCTCAACAA-3’  5’-ACTTCAACCCGGTCGTACAG-3’  5’-GTTACTCGGTGCCGATCTTC-3’  5’-CAGTCCGTTCAACTCCGTCT-3’  5’-TACTCTCTCTCGGCCTGCTC-3’  5’-TTGGCGGTAGAGGTGGATAG-3’  5’-GAGCAGCACCTGGAAGAAAC-3’  5’-ACGTGGCAATTTCCAGATTC-3’  5’-GAGCACACCGAGAACAATCA-3’  5’-TTGACCATCGCCTTGATGTA-3’  5’-AAGGATCGGCATTCGTACTG-3’  5’-GCATCGAAAACCAGAAGCTC-3’  5’-GAGACATCTTCGGCTTCGAC-3’  5’-GCCTGGCAATACTCGAACTC-3’  5’-CTGGACAGCAACTTCCCCGCGCTGCTGGTGGTCGACGTC-3’  5’-GACGTCGACCACCAGCAGCGCGGGGAAGTTGCTGTCCAG-3’  5’-ATGGCTGAGGCCGCCGCCGTATCCCACCGCATCGCCATCATCGACGACGACGT-3’  5’-AGCGTGTCTTCAACCGCCGCATCGGCGTCGGGACGCAGCCCGCCCAGCA-3’  5’-GCCGAAGGCTCGGCCGCAATCGTCGGCAACAGCGACAGCA-3’  5’-GCGTTCGCGCAGCGGTGGCAGCTGGATCGGGTATTCGT-3’  5’-GCAGGGCCTGCACGCCGAAACCATGCGCCAGGCCGAAGA-3’  5’-TCAGCGCCGCTTGCGCTTGTCGCTCAGGCGGTTGTGC-3’  5’-TTGCGGACAGAAGCGCCTATGGACCGTCTTTCCTGCTGGGTGGTCGACGACGA-3’  5’-GCCCAGGTTGCCCTGCTGCGCCTCCAGTGCACGCGCGGCCAGC-3’  5’-GAGGCGCAGCAGGGCAACCTGGGCCTGATCGGCGACACCCCGGCGA-3’  5’-GCGCTCGCGCAGGGGCGGCACCTGCAGCCGCACCACGT-3’  5’-GCTGATTCCGGCCGGCTGGGCCCGCGAACGCCTGGACAA-3’  5’-TCACGCGTTGTCCCCCGGCCCCAGCTTGC-3’ |
