# Supplementary figures and images for: Co-regulation of Iron Metabolism and Virulence Associated Functions by Iron and XibR, a Novel Iron Binding Transcription Factor, in the Plant Pathogen Xanthomonas
Source: PLoS Pathog. 2016 Nov 30;12(11):e1006019. doi: 10.1371/journal.ppat.1006019 (PMC5130282; doi:10.1371/journal.ppat.1006019)

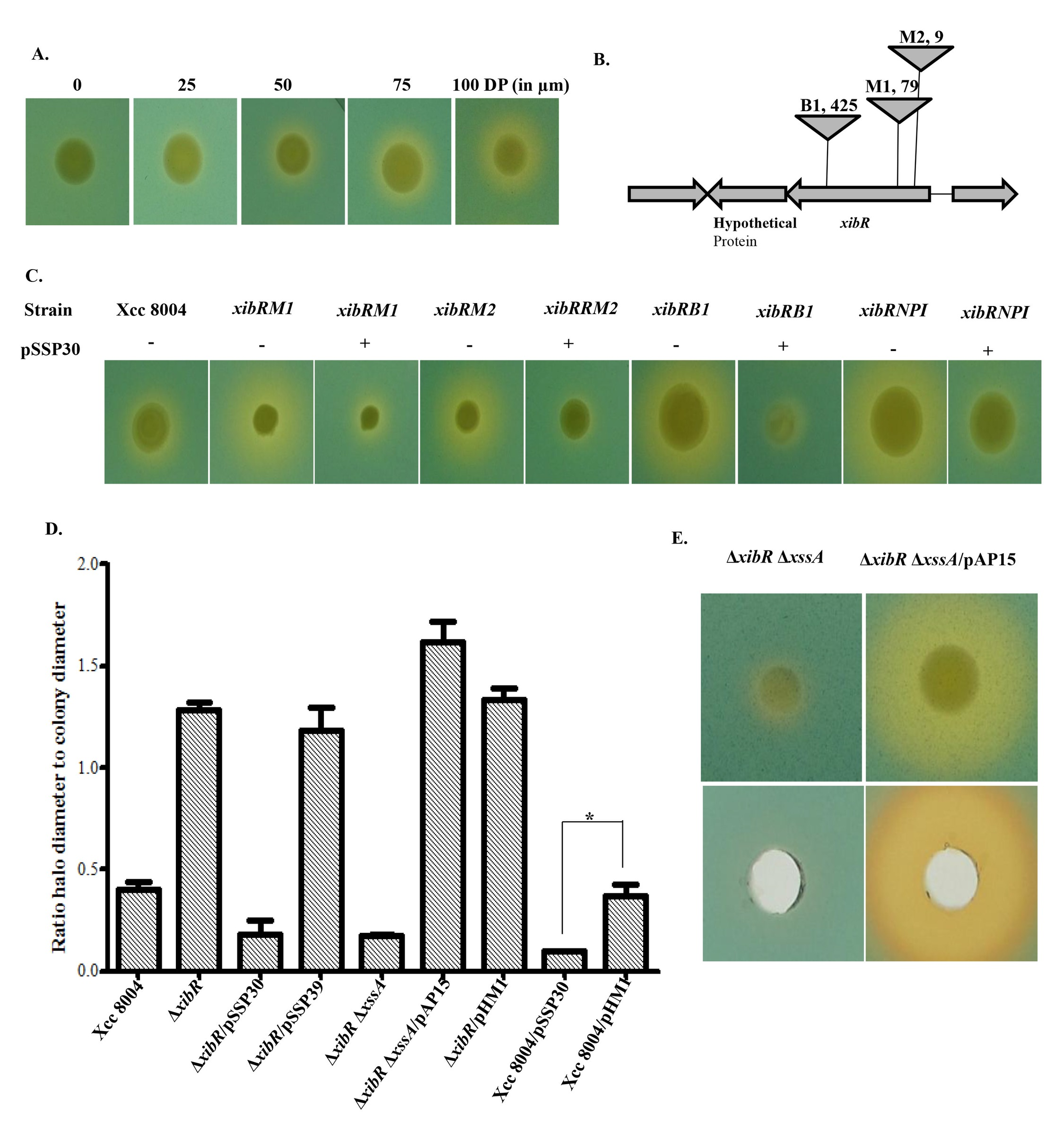

Supplement: S1 Fig — (A) Siderophore production by the wild-type Xanthomonas campestris pv. campestris 8004 after 48 h of growth on PSA-CAS plate supplemented without or with different concentration of iron specific chelator 2,2'-dipyridyl (DP). (B) Location of the mTn5 insertions and gene organization in the Xanthomonas campestris pv. campestris (Xcc 8004) genomic region containing the xibR gene. The arrows indicate transcriptional orientations of the genes. The xibR encodes a NtrC family of transcriptional regulator of 433 aa. The xibRM2, xibRM1 and xibRB1 mutants carry the mTn5 insertions at 9th, 79th and 425th codon of xibR, indicated by inverted triangles. (C) The transposon induced mutants xibRM1, xibRM2, and xibRB1, and a non-polar insertional mutant xibRNPI overproduce siderophore, indicated by the presence of an extended halo around the colony grown on peptone-sucrose agar plates containing chrome azurol sulphonate (CAS) + 75 μM 2,2’ dipyridyl (PSA-CAS + DP). Wild-type level of siderophore are restored by the addition of plasmid pSSP30 (wild-type xibR allele cloned in pHM1), indicated by + sign. (D) Quantification of siderophore production. Average ratio of siderophore halo to colony diameter for different strains of Xcc grown on PSA-CAS-DP plate. Strains: Xcc 8004 (wild-type strain), ΔxibR (xibR deletion mutant), ΔxibRΔxssA [xibR and xssA (X anthomonas siderophore synthesis A) double mutant], and strains harboring the plasmid containing either the wild-type xibR allele (pSSP30) or a point mutant of xibR in the putative conserved aspartate residue phosphorylation site (D55AXibR; pSSP39), xssA (pAP15; wild-type xssA allele) and pHM1 (vector). * indicate P < 0.05 in student’s t test (T-test) significant difference in the siderophore production between the wild-type Xcc 8004 harboring the plasmid containing the wild-type xibR allele (pSSP30) compared to the strain harboring the vector control (pHM1). Error bars represent SD of the mean (n = 3). (E) Siderophore production phenotype [file ppat.1006019.s018.tif]

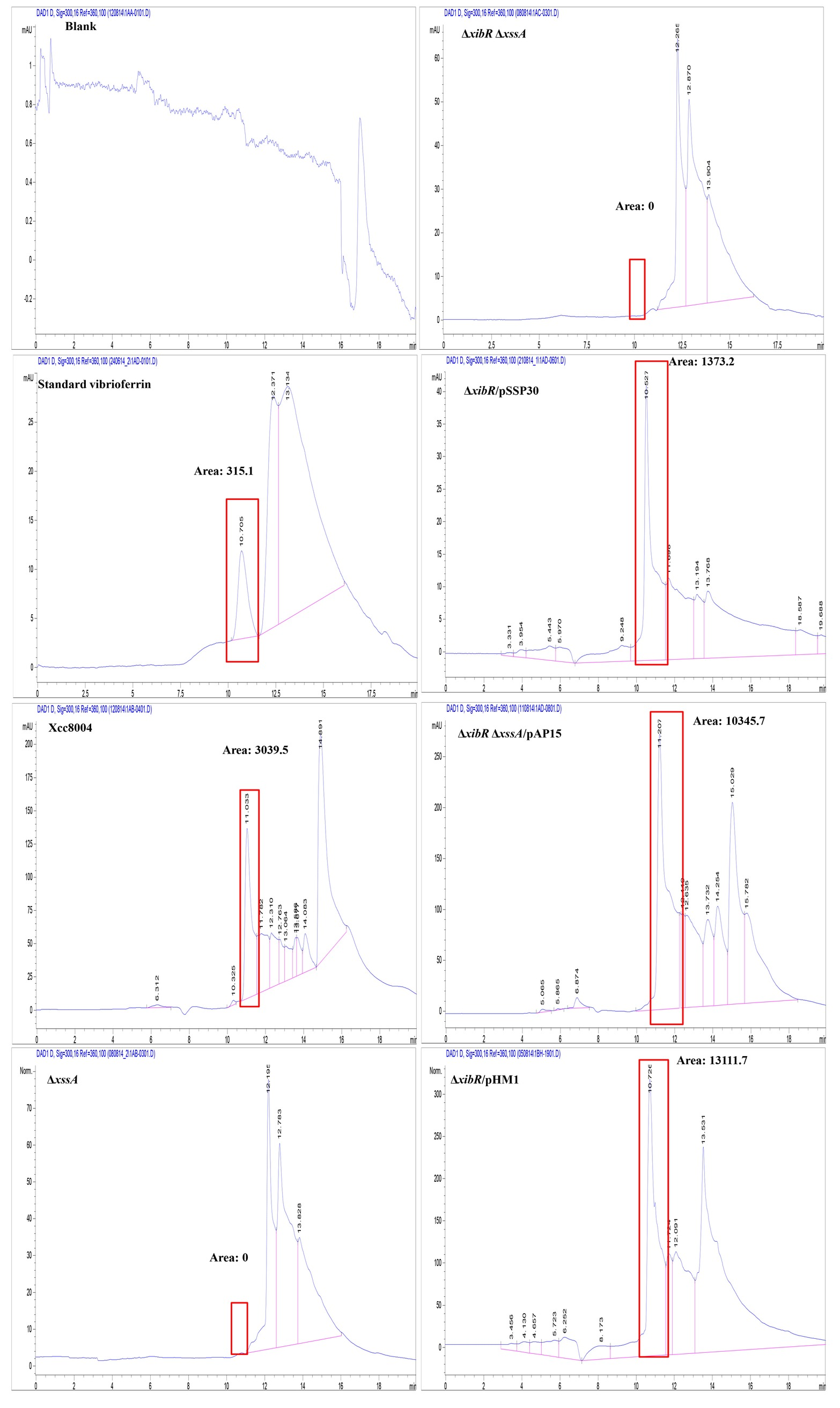

Supplement: S2 Fig — Representative HPLC chromatogram of siderophore isolated from the cell-free culture supernatants of wild-type Xcc 8004, ΔxssA (Xanthomonas siderophore synthesis A), ΔxibR, ΔxibR ΔxssA (xibR and xssA double deletion mutant), and strains harboring either the plasmid containing the wild-type xibR allele (pSSP30), the vector control (pHM1) or wild-type xssA allele (pAP15). Siderophore was isolated by Amberlite XAD-16 resin column chromatography and analyzed by HPLC (see supporting experimental procedures). Vibrioferrin peak was detected at 300 nm. Red color inset indicate the vibrioferrin peak corresponding to the standard purified vibrioferrin. (TIF) [file ppat.1006019.s019.tif]

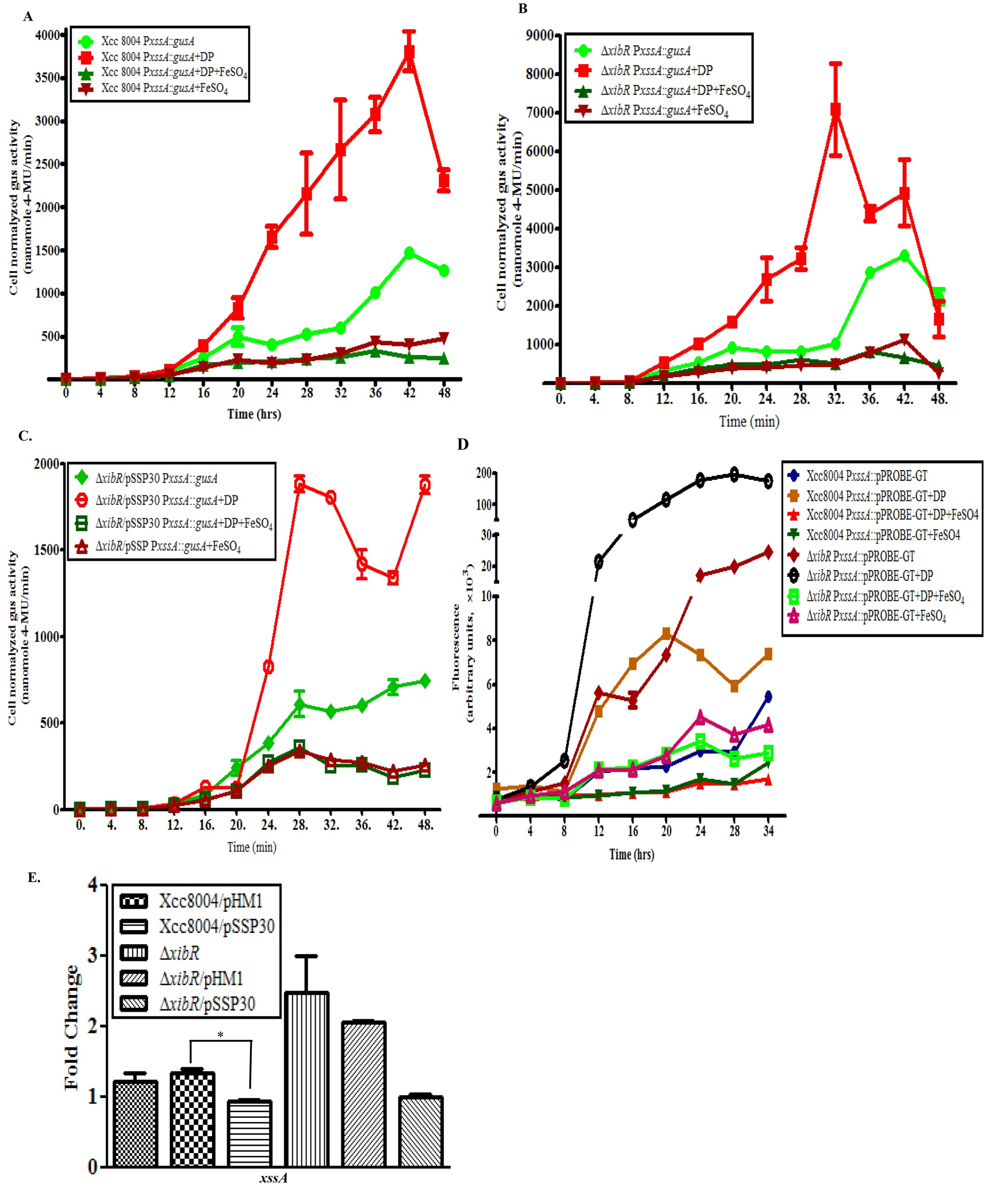

Supplement: S3 Fig — Expression analysis was carried out with the β-glucuronidase (GUS) chromosomal reporter fusions (PxssA:: gusA) in the wild-type (Xcc 8004 PxssA::gusA) (A), ΔxibR (ΔxibR PxssA::gusA) (B), ΔxibR mutant harboring the complementing plasmid pSSP30 (ΔxibR/pSSP30 PxssA::gusA) (C). Strains were grown either in rich PS medium or PS medium supplemented with 100 μM DP (low-iron condition), 50 μM FeSO4 (iron-replete condition), and 100 μM DP + 100 μM FeSO4. β-Glucuronidase (GUS) activity was measured at 365/455 nm excitation/emission wavelength respectively and represented as cell normalized nanomoles of 4-methyl-umbelliferone (4-MU) produced per minute. Data are shown as mean ± S.D. (n = 3). (D) Transcriptional analysis of plasmid borne PxssA::gfp expression in wild-type Xcc 8004 and ΔxibR strain. Relative GFP fluorescence of wild-type Xcc 8004 and ΔxibR strain harboring the GFP reporter plasmid pPROBE-GT (PxssA:: pPROBE-GT). Strains were grown either in rich PS medium or PS medium supplemented with 100 μM DP (low-iron condition), 50 μM FeSO4 (iron-replete condition), and 100 μM DP + 100 μM FeSO4. The error bars represent the standard deviations of the mean cell-normalized GFP fluorescence. Data are shown as mean ± S.E. (n = 3). (E) Relative quantification of expression of the siderophore biosynthesis gene (xssA) of Xcc by real-time qRT-PCR. The wild-type Xcc 8004 and ΔxibR strains harboring either the plasmid containing the wild-type xibR allele (pSSP30) or the vector pHM1 (control), were grown to OD600 1.2 in PS medium containing 100 μM 2′2,dipyridyl (DP). 16S ribosomal RNA was used as an endogenous control to normalize the RNA for cellular abundance. Data are shown as mean ± S.E. (n = 3). (TIF) [file ppat.1006019.s020.tif]

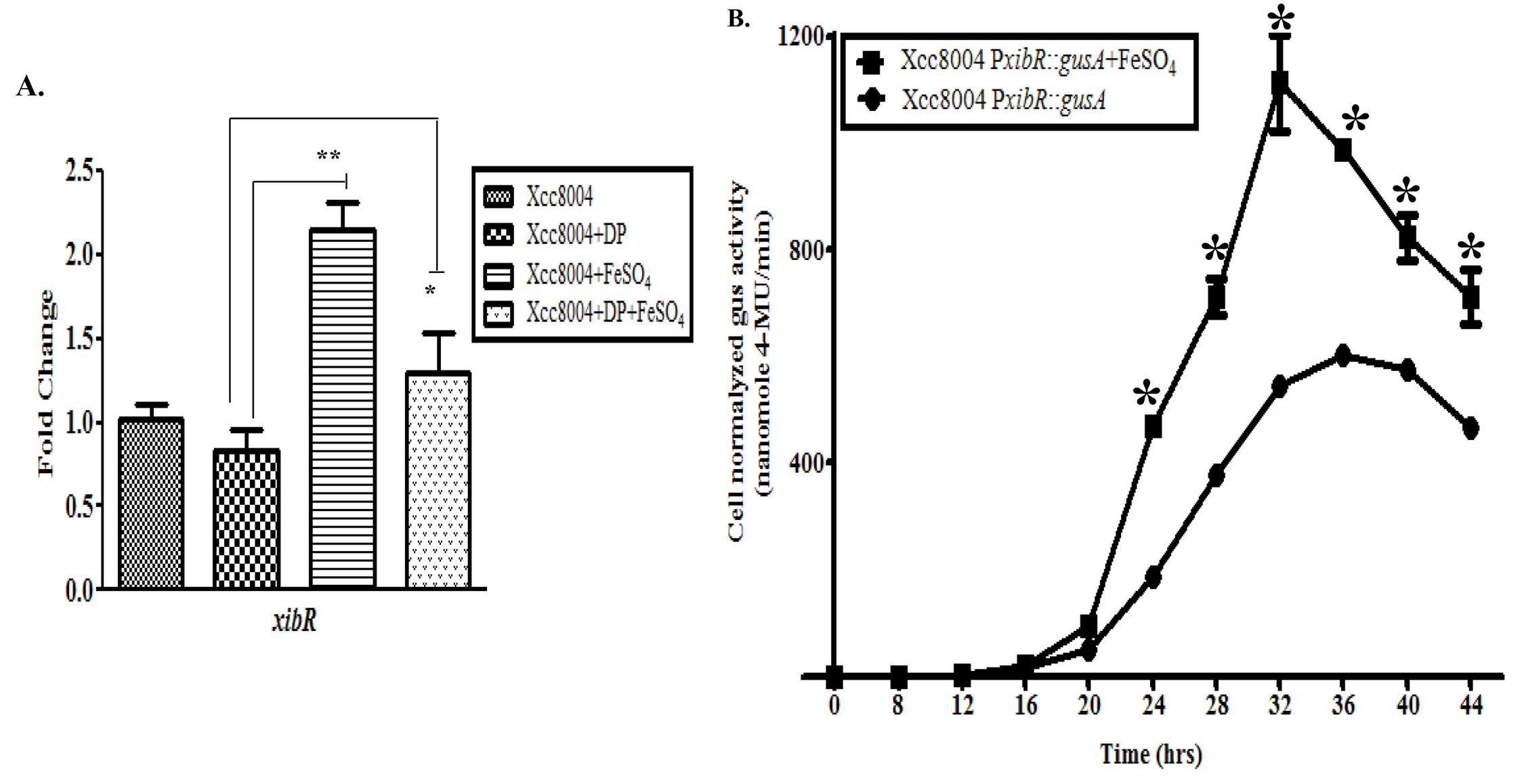

Supplement: S4 Fig — (A) Relative quantification of expression of the xibR in the wild-type Xcc 8004 strain grown in PS (rich medium), PS + 100 μM FeSO4 (iron-replete), PS + 100 μM DP (low-iron), and PS + 100 μM DP + 100 μM FeSO4 by real-time qRT-PCR. ** P < 0.01 and * P < 0.05 in Student’s t test. Data shown in the graphs as mean ± S.E. (n = 3) (B) Transcriptional analysis of xibR gene in Xcc. Expression analysis was carried out with the β-glucuronidase (GUS) chromosomal reporter fusion (PxibR:: gusA) in the wild-type (Xcc 8004 PxibR::gusA) strain grown either in PS medium or supplemented with 50 μM FeSO4. Error bars represent SD of the mean (n = 3) cell normalized Glucuronidase (GUS) activity represented as nanomoles of 4-methyl-umbelliferone (4-MU) produced per minute. * indicates P < 0.01 in Student’s t test, significant difference between the data obtained for the wild-type Xcc 8004 PxibR:: gusA strain grown in PS medium compared to those obtained from growth under iron-replete condition (PS + 50 μM FeSO4). (TIF) [file ppat.1006019.s021.tif]

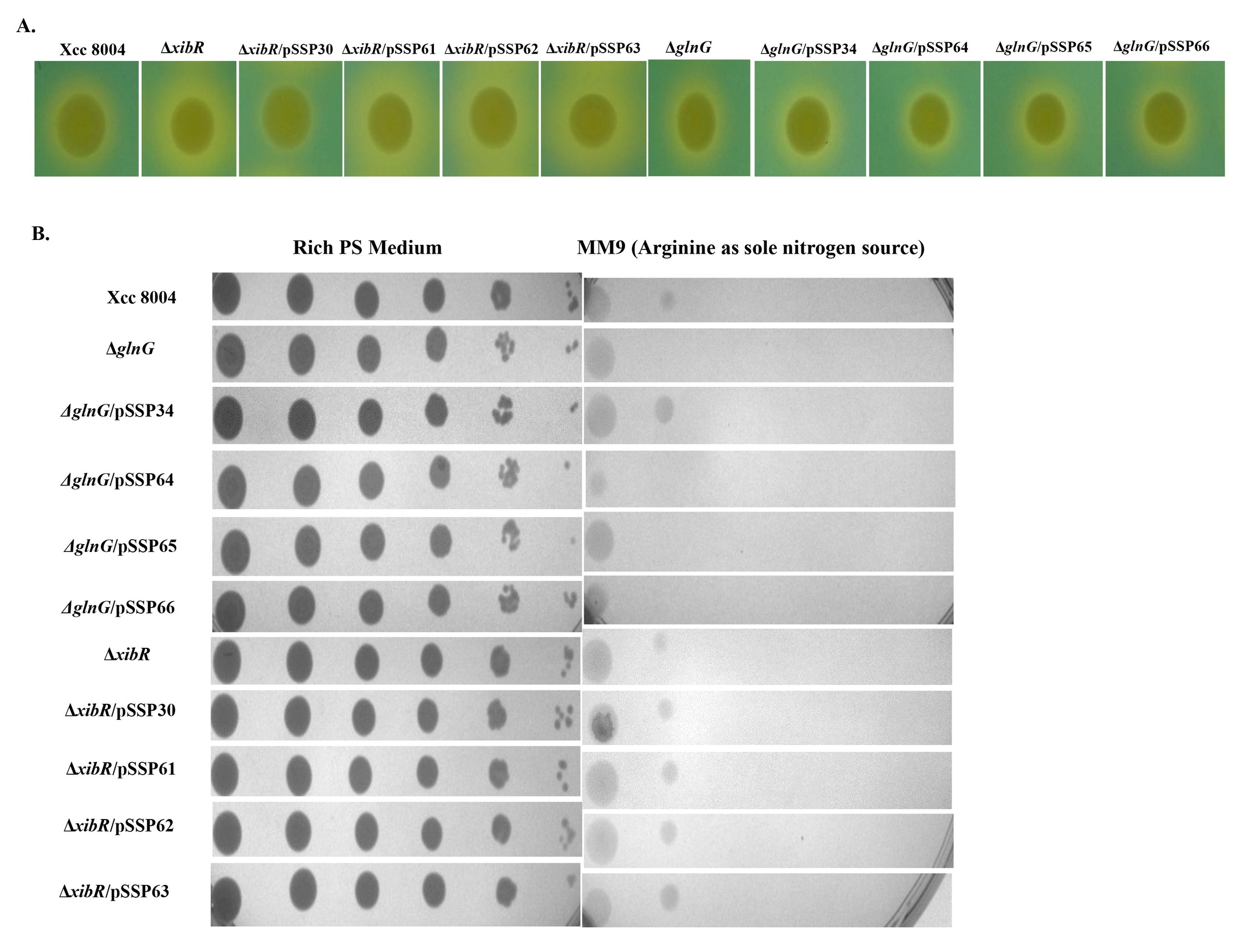

Supplement: S5 Fig — (A) Siderophore production on PSA-CAS-DP plates by different Xcc strains: Xcc strains: Xcc 8004 (wild-type), ΔxibR (xibR deletion mutant), ΔglnG (glnG deletion mutant), ΔxibR/pSSP30 (ΔxibR mutant harboring the plasmid containing the wild-type xibR allele; XibR), ΔglnG/pSSP34 (ΔglnG mutant harboring the plasmid containing wild-type glnG or ntrC allele; NtrC), ΔxibR (pSS61; XibR Swp Rec), ΔxibR (pSS62; XibR Swp σ54), ΔxibR (pSS63; XibR SwpHTH), ΔglnG (pSS64; NtrC Swp Rec), ΔglnG (pSS65; NtrC Swp σ54) and ΔglnG (pSS66; NtrC SwpHTH). (B) Serial dilution spotting assay of different Xcc strains on modified MM9 minimal medium plates containing arginine as a sole nitrogen source. (TIF) [file ppat.1006019.s022.tif]

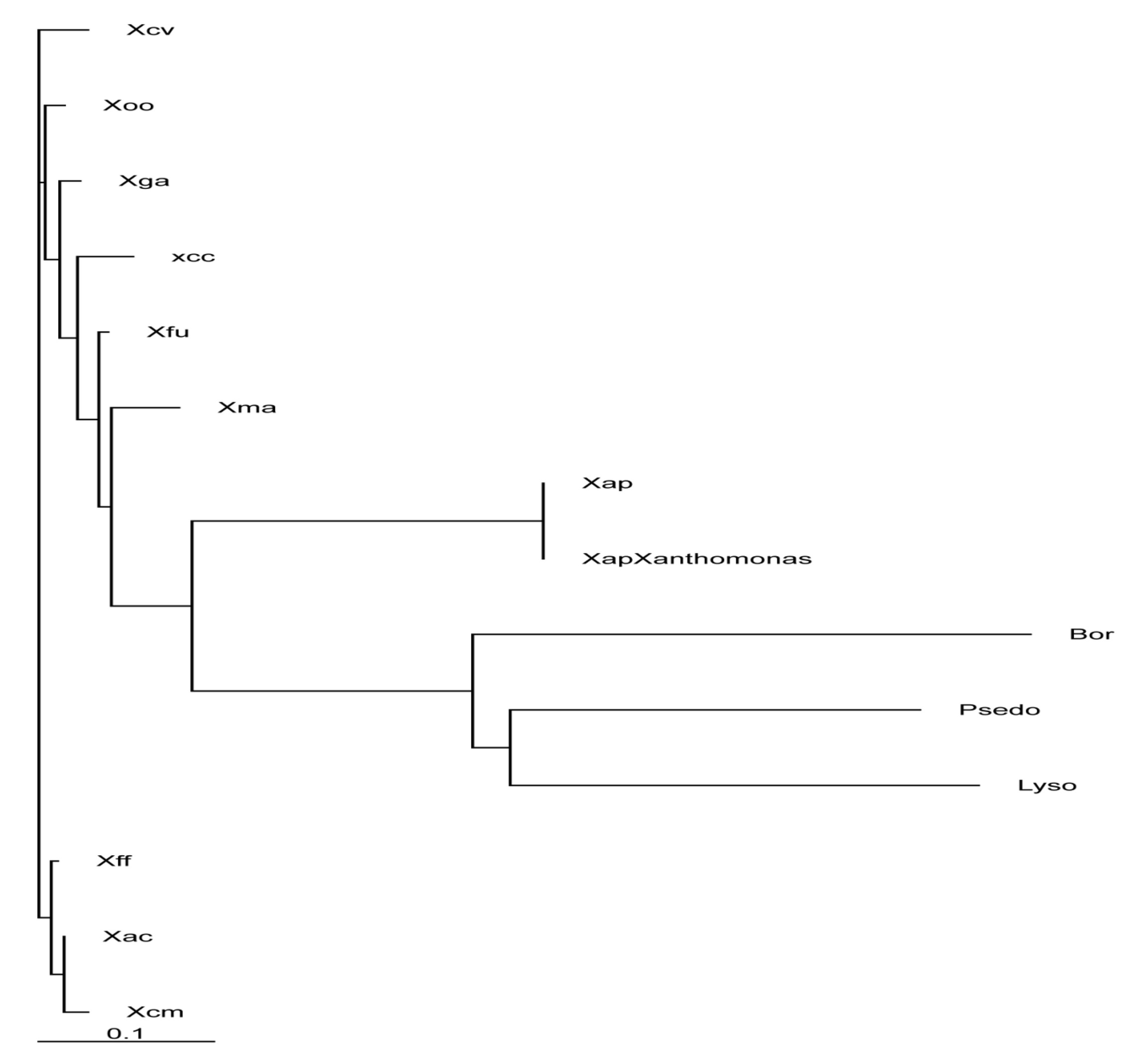

Supplement: S6 Fig — Phylogenetic dendrogram of XibR homolog’s in NCBI database was constructed by using the UPGMA method after amino acid sequence alignment with ClustalW and phylip 3.67 (mobyle.pasteur.fr/cgi-bin/portal.). Xanthomonas campestris pv. campestris str. 8004 (Xcc; AAY50800); Xanthomonas fuscans subsp. fuscans (Xff; CDF63051); Xanthomonas axonopodis pv. citri str. 306 (Xac; AAM38576); Xanthomonas gardneri (Xga; WP_046933196); Xanthomonas arboricola pv. pruni MAFF 301420 (Xap; GAE55687); Xanthomonas oryzae pv. oryzae KACC 10331 (Xoo; AAW73895); Xanthomonas vesicatoria (Xcv; WP_005988114); Xanthomonas maliensis (Xma; WP_022971710); Pseudoxanthomonas dokdonensis (Psedo; KRG68042); Lysobacter sp. URHA0019 (Lyso; WP_027082117); Bordetella bronchiseptica (Bor; WP_003811339). Scale 0.1 represents 10% differences between two sequences. (TIF) [file ppat.1006019.s023.tif]

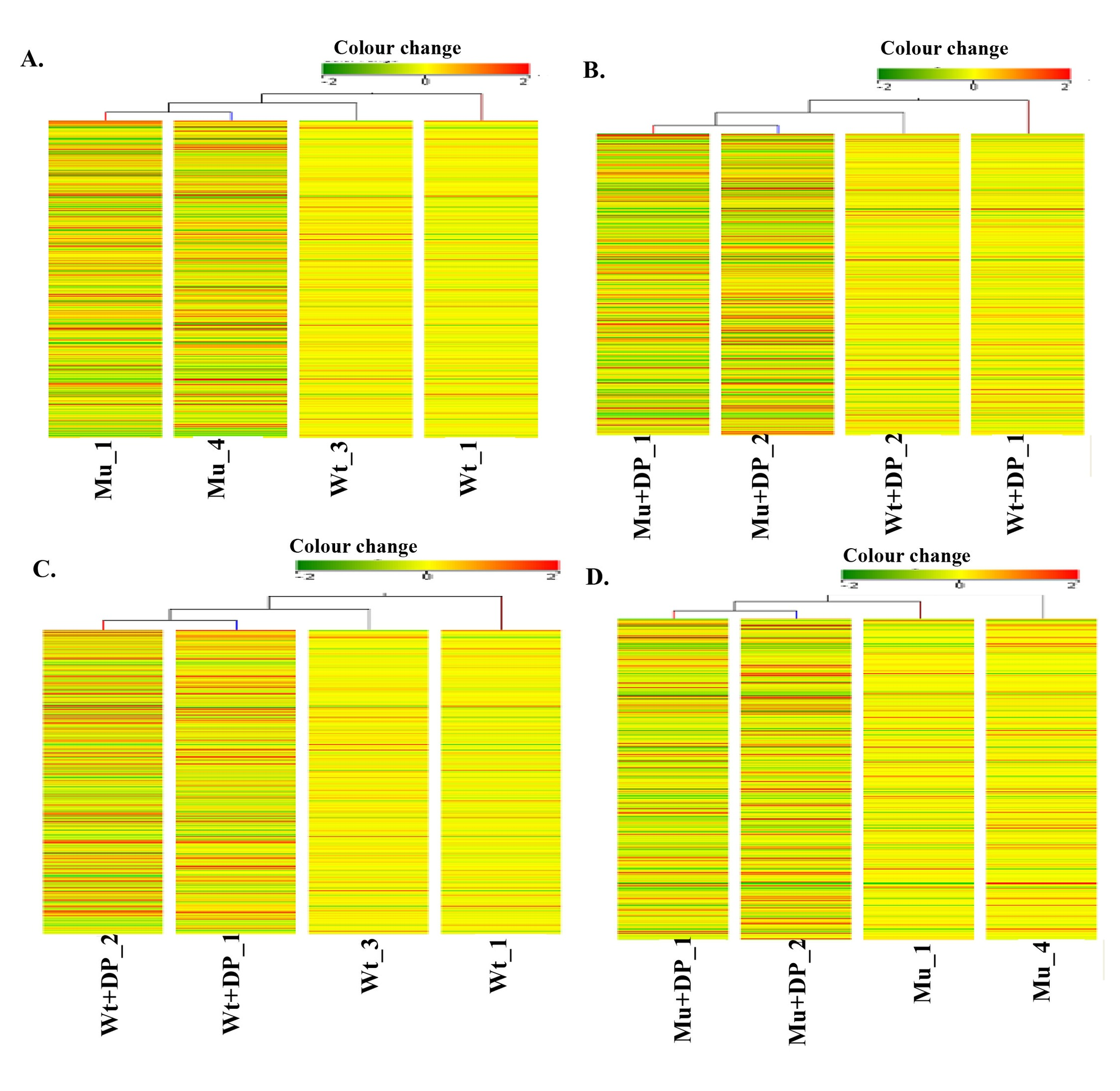

Supplement: S7 Fig — Heat map was generated using GeneSpringGX Software using the geomean fold (Log2) expression values of (A) ΔxibR mutant versus wild-type Xcc 8004 grown in PS medium (iron-replete condition)under rich medium; (B) ΔxibR mutant versus wild-type Xcc 8004 under low-iron condition (PS + DP); (C) wild-type Xcc 8004 grown under low-iron condition versus that grown under iron-replete condition; and (D) ΔxibR mutant grown under low-iron condition versus that grown under iron-replete condition. Color scale indicates log2 –fold change of expression (from green for downregulated to red for upregulated). (TIF) [file ppat.1006019.s024.tif]

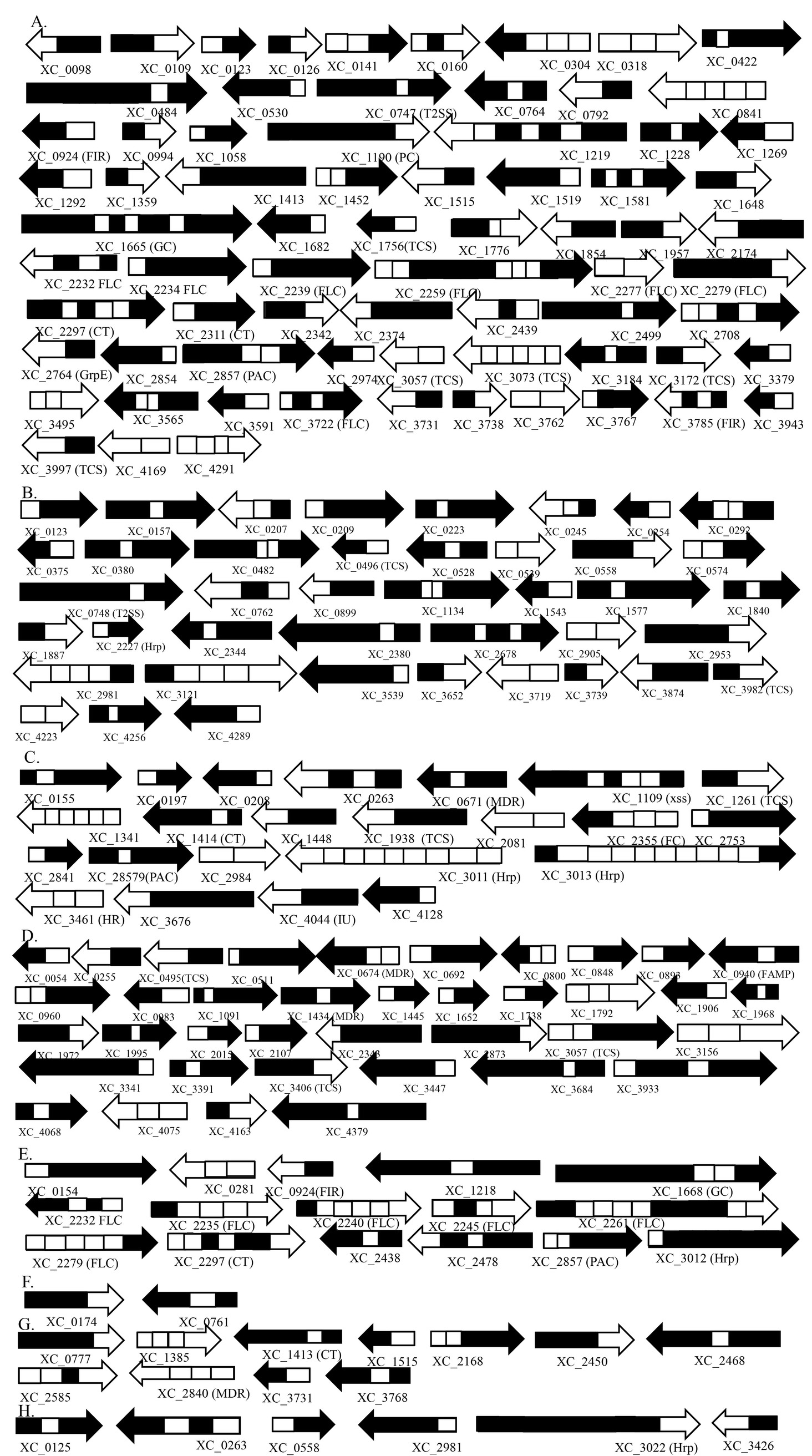

Supplement: S8 Fig — Predicted operon's which are either positively regulated (A) or repressed (B) by XibR but are not affected by low-iron condition. Operons which are either up-regulated (C) or down-regulated (D) under low-iron condition but are not affected by XibR. Operons which are either positively (E) or negatively (F) regulated by both XibR and low-iron. (G) Operons which are positively regulated by XibR and repressed by low-iron. (H) Operons which are repressed by XibR and induced under low-iron condition. Arrow indicates the direction of transcription of each predicted operon. The genes not differentially expressed in microarray were depicted as black boxes. (TIF) [file ppat.1006019.s025.tif]

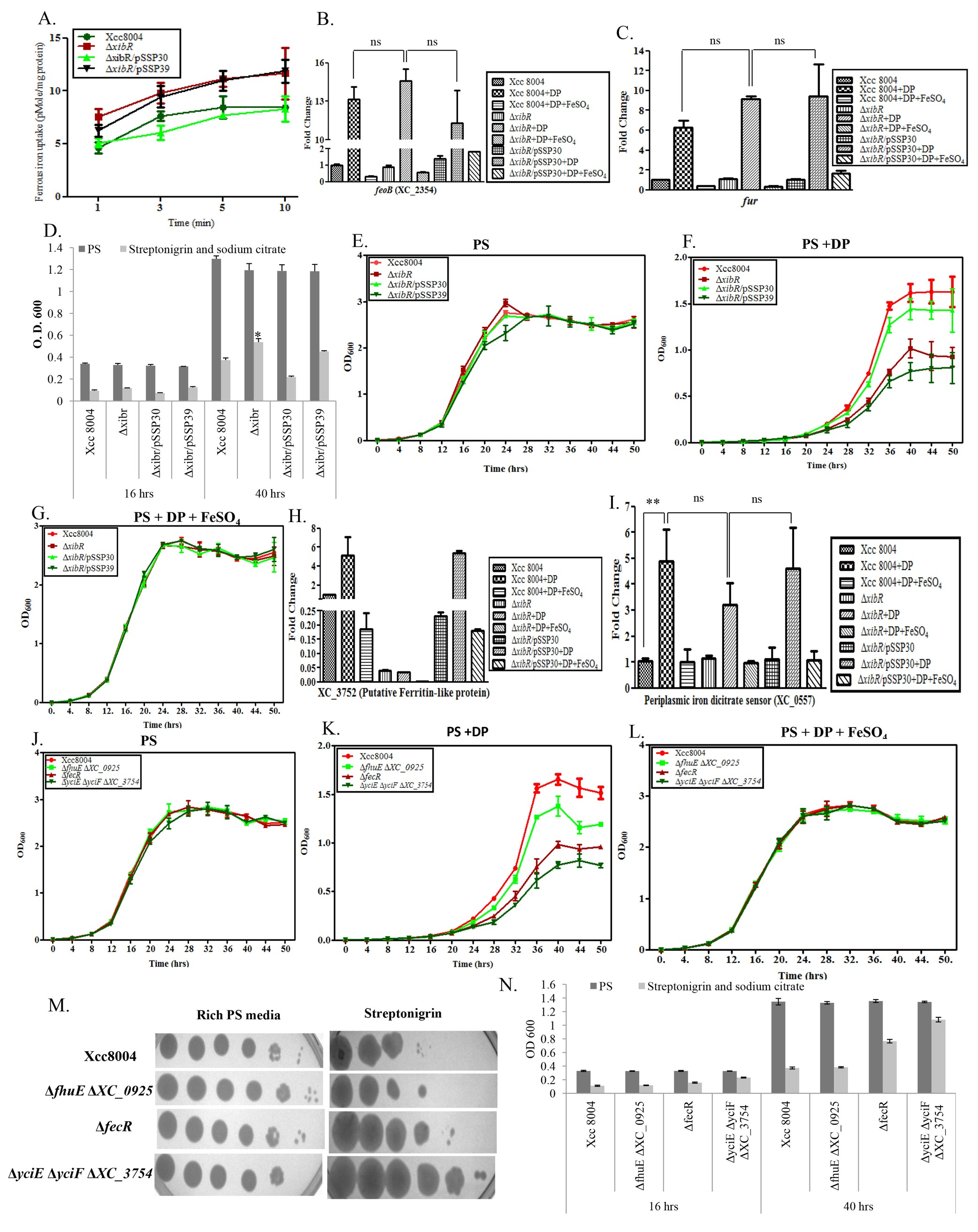

Supplement: S9 Fig — (A) ΔxibR mutant do not exhibit any defect in Fe2+ uptake. Incorporation of radiolabelled Fe2+ by Xcc 8004, ΔxibR, and strains harbouring the plasmid containing either the wild-type xibR allele (pSSP30) or a point mutant of xibR in the putative conserved aspartate residue phosphorylation site (D55AXibR; pSSP39). 55FeCl3 was reduced to 55Fe2+ in 1M sodium ascorbate. Uptake assay was performed in the presence of sodium ascorbate to maintain the FeCl3 in the reduced form. Data are shown as mean ± S.E. (n = 3). (B and C) Relative quantification of expression of the ferrous iron transporter (feoB) and ferric uptake regulator (fur) of Xcc by real-time qRT-PCR. RNA was isolated from Xcc 8004, ΔxibR and strain harboring the plasmid containing the wild-type xibR allele (pSSP30) grown under PS, PS + 100 μM DP and PS + 100 μM DP + 100 μM FeSO4. 16S ribosomal RNA was used as an endogenous control to normalize the RNA for cellular abundance. Data are shown as mean ± S.E. (n = 3), ns = not significant. (D) Absorbance at 600 nm of Xcc strains grown in PS broth with or without 0.5 μg/ml SNG and 0.01M sodium citrate after 16 and 42 h of growth are shown. Data are shown as mean ± S.E. (n = 3). (E-G). The growth of Xcc 8004, ΔxibR, ΔxibR/pSSP30 and ΔxibR/pSSP39 strains in rich PS medium (E), low-iron medium (PS + intracellular ferrous iron chelator 150 μM 2′2′-bipyridyl) (F), and low-iron medium supplemented with iron (PS + 150 μM 2′2′-bipyridyl + 100 μM FeSO4) (G). Growth was monitored by determining the OD600. Data are shown as mean ± S.E. (n = 3). (H and I) Relative quantification of expression of the putative ferritin-like protein (XC_3752) and periplasmic iron dicitrate sensor (XC_0557) of Xcc by real-time qRT-PCR. RNA was isolated from Xcc 8004, ΔxibR and strain harboring the plasmid containing the wild-type xibR allele (pSSP30) grown in rich PS media, PS + 100 μM DP and PS + 100 μM DP + 100 μM FeSO4. 16S ribosomal RNA was used as an endogenous control to normalize the RNA for c [file ppat.1006019.s026.tif]

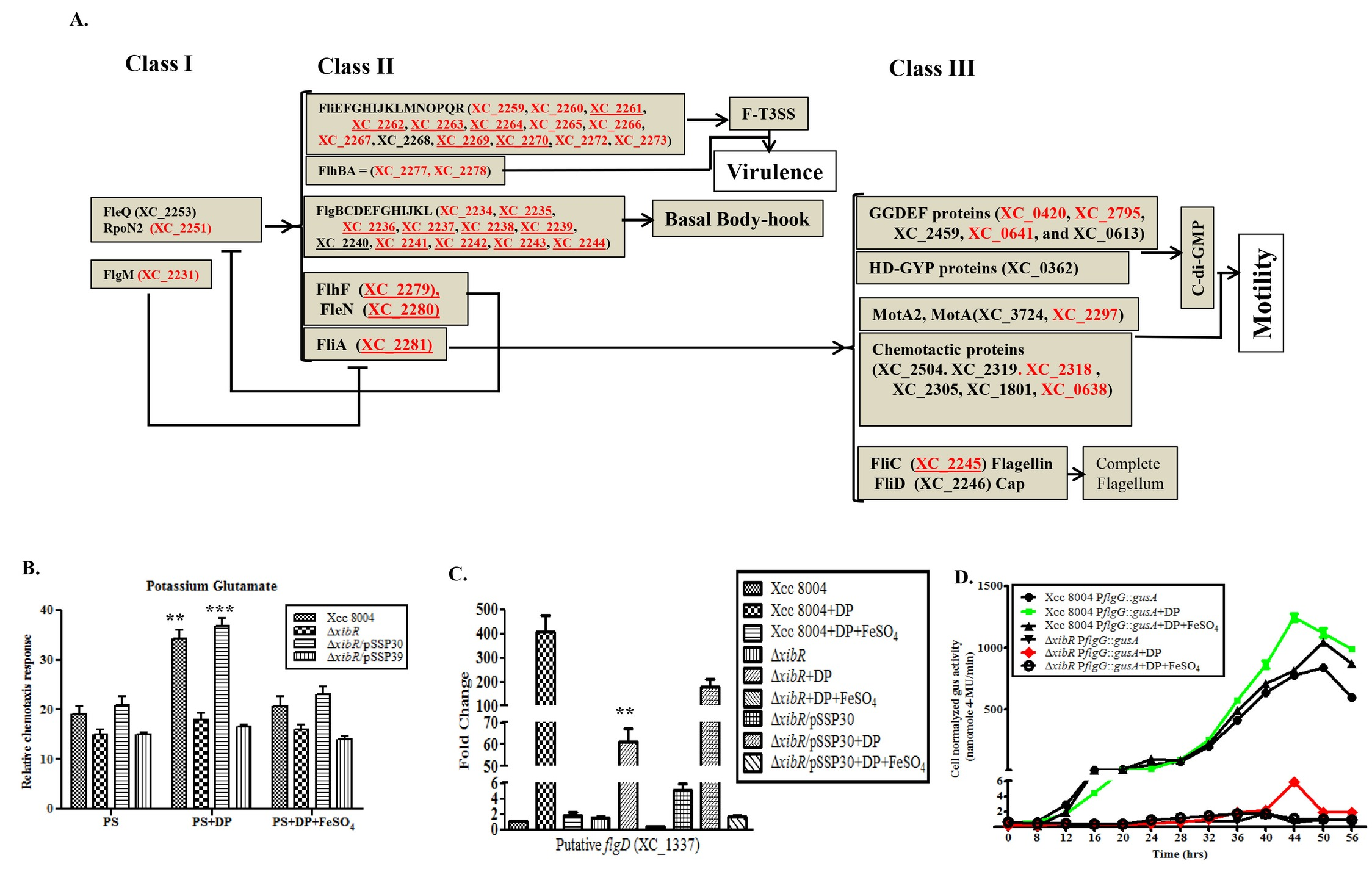

Supplement: S10 Fig — (A) Schematic representation of the model of the flagellar transcriptional cascade in Xcc. Expression and assembly of flagellar components takes place in a temporal fashion, in which the Class I protein σ54 and FleQ regulates the expression of class II genes, which are required for site selection and basal body formation. Class III genes encode proteins required for flagellar filament, cap proteins and motility regulatory proteins. Locus tags of flagellar genes encoding proteins are shown in bracket. Based on expression analysis by microarray, genes (locus tags) which are positively regulated by XibR are shown in red color. Genes (locus tags) which are positively regulated by both XibR and low-iron condition are shown as underline. Genes which are not differentially expressed in microarray were depicted in black color. (B) Quantitative chemotaxis capillary assay with different Xcc strains grown under PS, PS + 100 μM DP and PS + 100 μM DP + 100 μM FeSO4. Cells were incubated at 28°C with capillaries containing potassium glutamate (4.9 mM) and PBS. Relative chemotaxis response was determined by migrated bacterial cells in capillary containing potassium glutamate over the capillary containing PBS. Data are shown as mean ± S.E. (n = 3). The experiment was repeated two times. (C) Relative quantification of the expression of flgD by real-time qRT-PCR. Different strains of Xcc; Xcc 8004, ΔxibR and strain harbouring the plasmid containing the wild-type xibR allele (pSSP30), were grown to OD600 1.2 in PS, PS + 100 μM DP and PS + 100 μM DP + 100 μM FeSO4. 16S ribosomal RNA was used as an endogenous control to normalize the RNA for cellular abundance. Data are shown as mean ± S.E. (n = 3). (D) Expression analysis of flgG operon in wild-type (Xcc 8004 PflgG::gusA) and ΔxibR (ΔxibRPflgG::gusA) grown under PS, PS + 100 μM DP and PS + 100 μM DP + 100 μM FeSO4 while monitoring the β-glucuronidase (GUS) activity. Data are shown as mean ± S.D. (n = 3). * Indicating p-value < 0.05, ** [file ppat.1006019.s027.tif]

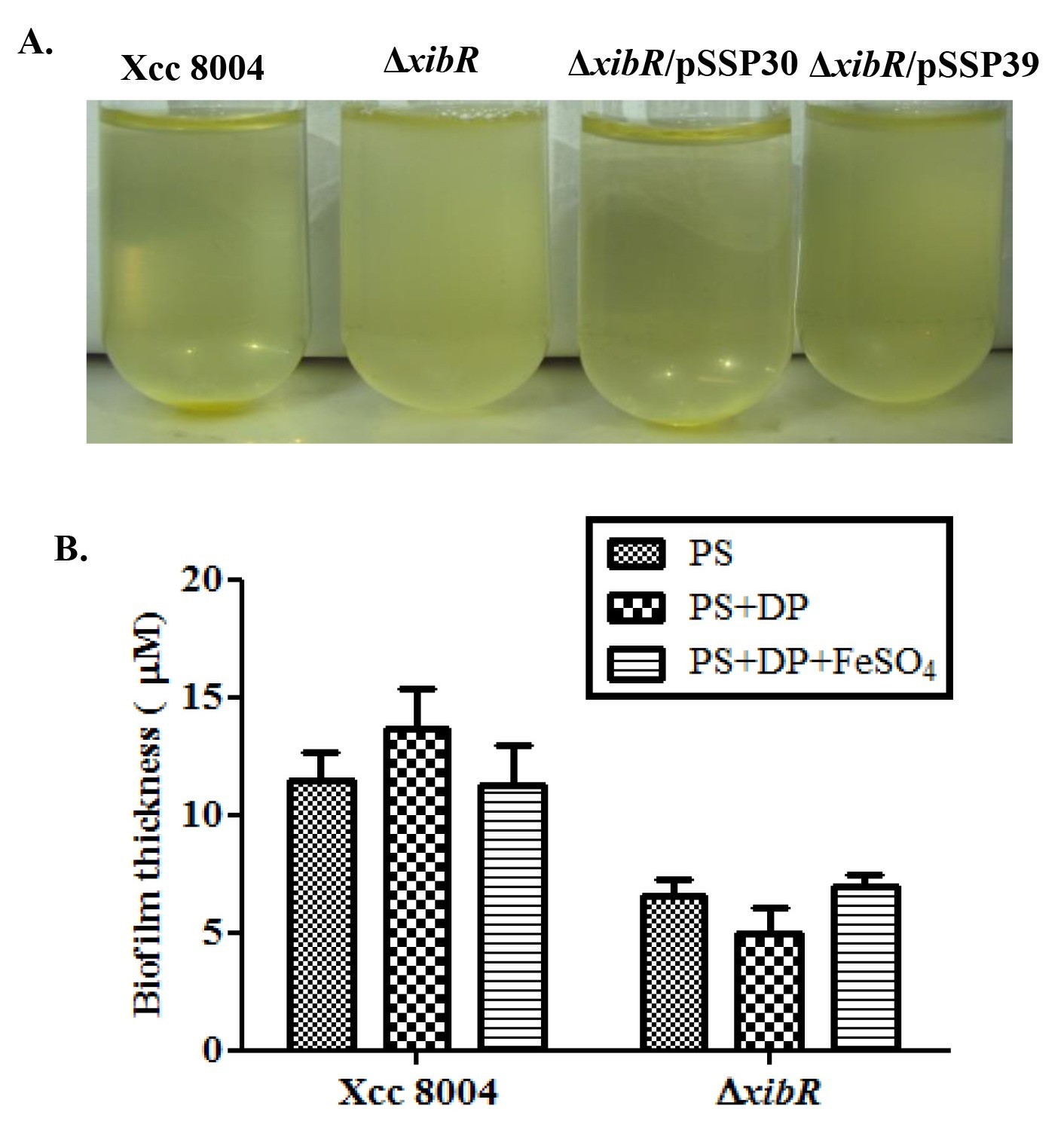

Supplement: S11 Fig — (A) Saturated cultures were grown in rich PS medium and the tubes were kept at room temperature for 4 hours for the observation of aggregation phenotype. Wild-type Xcc 8004 and ΔxibR/pSSP30 exhibit disperse phenotype than ΔxibR and ΔxibR/pSSP39. (B) Average biofilm thickness of different strains of Xcc formed on the glass slide at the air-media interphase. Different Xcc strains were inoculated in PS, low-iron (PS + 100 μM DP) and iron supplemented (PS + 100 μM DP + 100 μM FeSO4) media at a concentration of 106 cells/ml and grown for 24 h. For quantification of the thickness, five independent biofilms were scanned with CLSM at ten randomly selected positions and thickness was determined through height of the biofilm. Data are shown as mean ± S.E. (n = 3). (TIF) [file ppat.1006019.s028.tif]

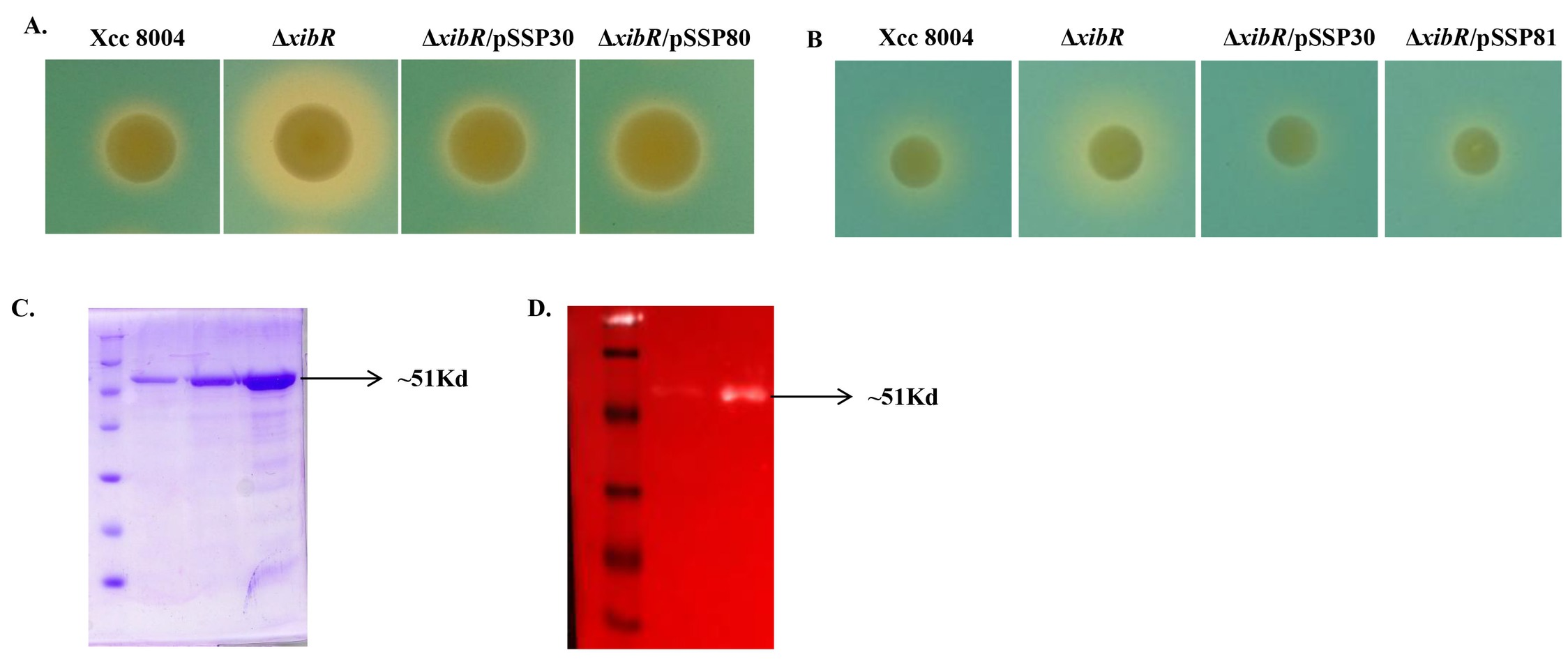

Supplement: S12 Fig — (A) wild-type Xcc 8004, ΔxibR, ΔxibR/pSSP30 and ΔxibR/pSSP80 (B) Strains wild-type Xcc 8004, ΔxibR, ΔxibR/pSSP30 and ΔxibR/pSSP81 (C) SDS-PAGE for purified XibR with C-terminal His-tag; lane 1 is Unstained Protein MW Marker (ThermoFisher Scientific, Waltham, MA, USA), lane 2, 3, and 4 are different fractions of purified XibR. (D) Western blot for XibR with C-terminal His-tag using anti-His antibody. Lane 1 = Prestained Protein MW Marker (ThermoFisher Scientific, Waltham, MA, USA); lane 2 = un-induced XibR in bl21 (DE3); and lane 3 = induced XibR in bl21 (DE3). (TIF) [file ppat.1006019.s029.tif]

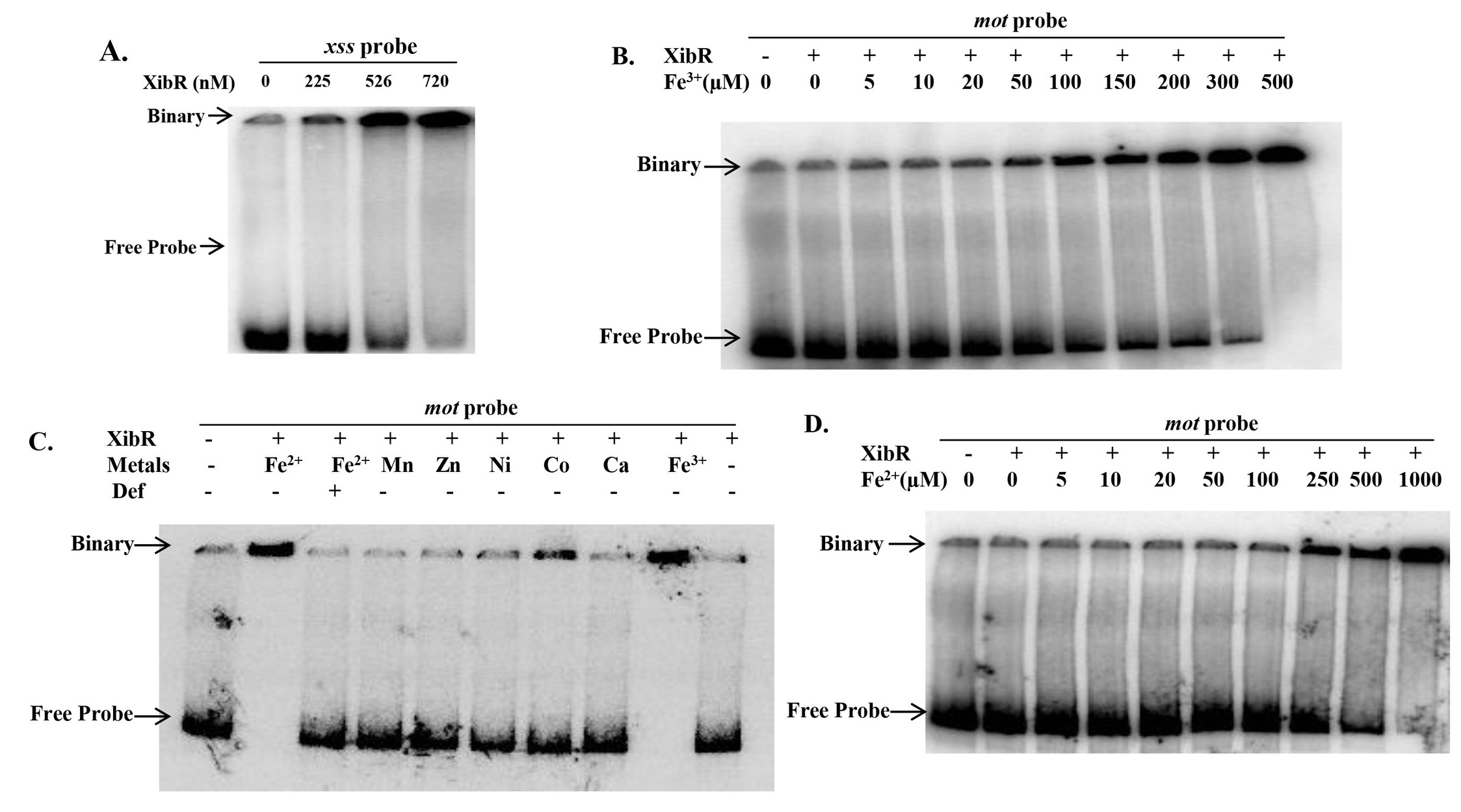

Supplement: S13 Fig — Electrophoretic mobility shift assay (EMSA) showing binding of XibR to a 32P-labeled motA probe with increasing concentration of either ferric (B) or ferrous (D) form of iron. (A) EMSA showing binding of XibR to a 32P-labeled xss (-188 to +205) probe. More DNA-protein binary complex was observed with the increase in the concentration of XibR protein. (C) EMSA showing binding of XibR to a 32P-labeled motA probe in the presence of other divalent metal ions and ferric iron. Presence of deferoxamine mesylate with FeSO4 decreased binding of XibR to the motA probe (lane 3). (TIF) [file ppat.1006019.s030.tif]

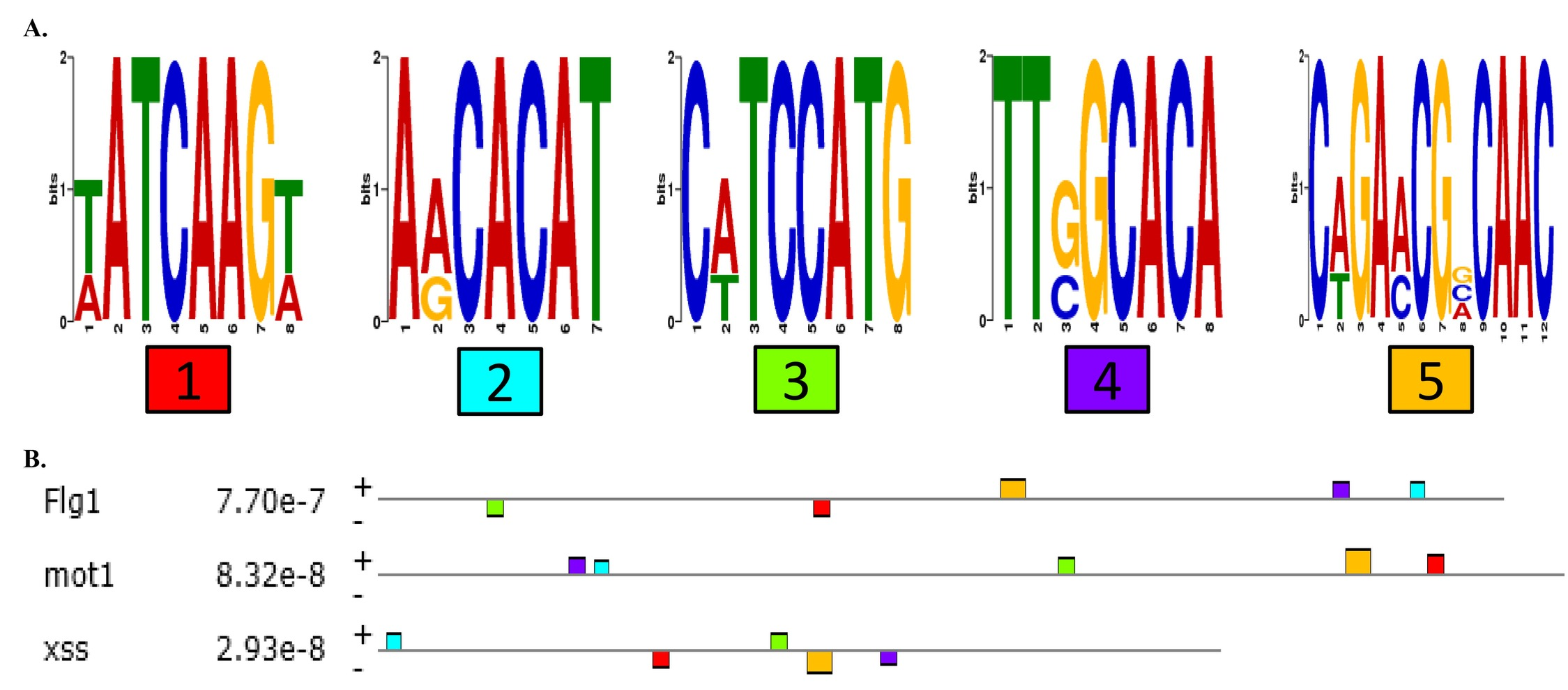

Supplement: S14 Fig — (A) Sequence logos for the five consensus motifs identified by MEME. (B) Schematic representation of relative position of conserved motifs (shown in red, blue, green, magenta and orange color boxes) on the flg, mot and xss promoter sequences. (TIF) [file ppat.1006019.s031.tif]

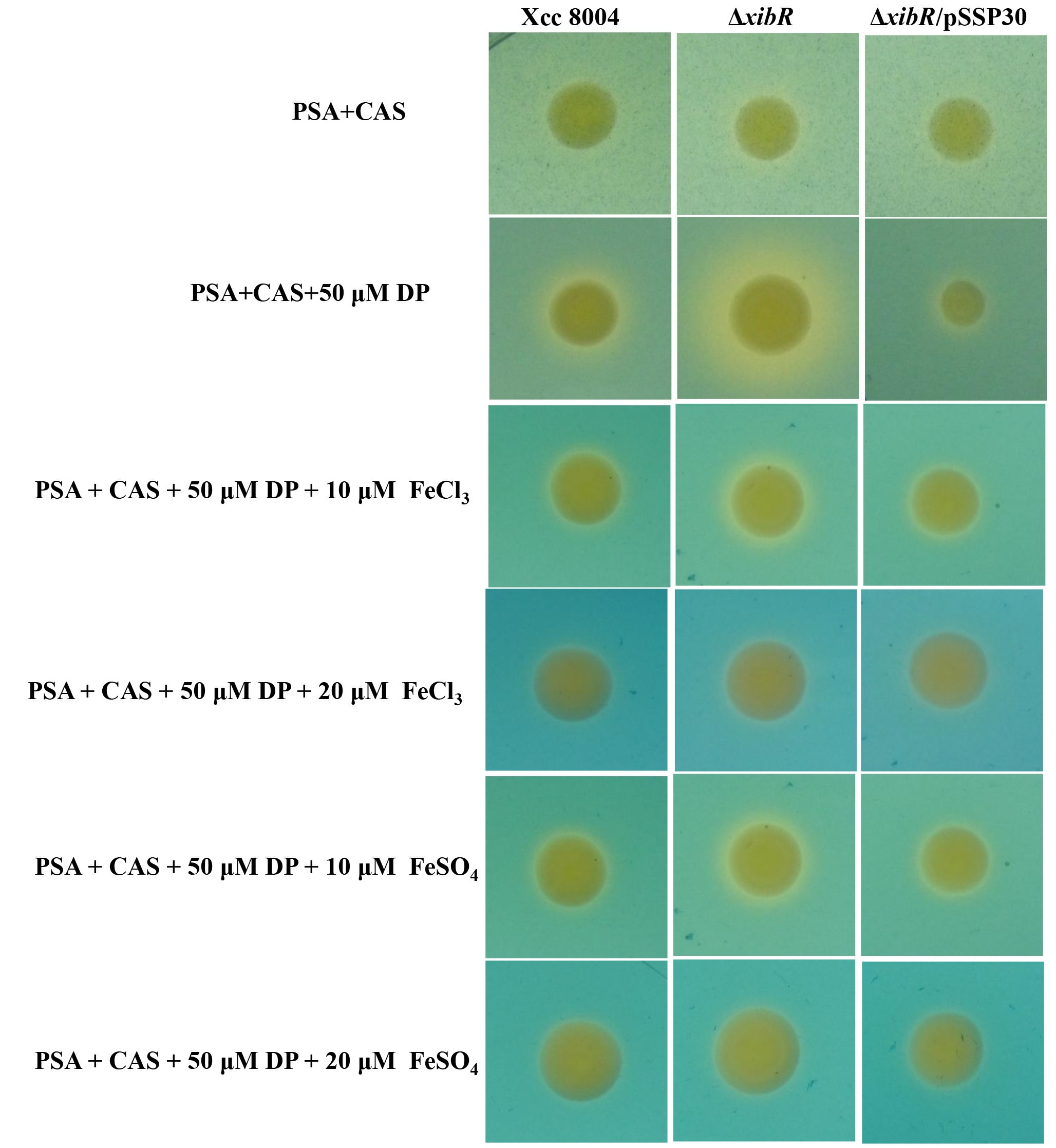

Supplement: S15 Fig — Different strains of Xcc were grown on PSA-CAS medium containing without or with 50 μM 2,2′-dipyridyl (DP). For iron supplementation, either FeCl3 or FeSO4 were added in PSA-CAS + DP medium at a concentration of 10 and 20 μM. (TIF) [file ppat.1006019.s032.tif]
